# Qualitative analysis of genomic mutations and antibiotic susceptibility testing of *Pseudomonas aeruginosa* isolates from chronic lung infections

**DOI:** 10.1371/journal.pone.0341613

**Published:** 2026-03-06

**Authors:** Ad C. Fluit, Jumamurat R. Bayjanov, María Díez Aguilar, Rafael Cantón, Deidre F. Gilpin, Michael M. Tunney, Miquel B. Ekkelenkamp

**Affiliations:** 1 Department of Medical Microbiology, University Medical Center Utrecht, Utrecht, The Netherlands; 2 Servicio de Microbiología, Hospital Universitario Ramón y Cajal and Instituto Ramón y Cajal de Investigación Sanitaria (IRYCIS), Madrid, Spain; 3 CIBER de Enfermedades Infecciosas (CIBERINFEC). Instituto de Salud Carlos III, Madrid, Spain; 4 Current affiliation: Servicio de Microbiología, Hospital Universitario La Princesa, Madrid, Spain; 5 Queen’s University Belfast, School of Pharmacy, Belfast, United Kingdom; University of Buea, CAMEROON

## Abstract

*P. aeruginosa* is intrinsically resistant to many antibiotics and may acquire resistance to others. The aim was to reconcile phenotypic resistance of isolates obtained from patients with chronic respiratory infection with the results of WGS. A total of 497 isolates were recovered from 4 countries between 2002 and 2016 from patients chronic pulmonary conditions, especially cystic fibrosis. Minimum inhibitory concentrations were determined previously by broth microdilution method. Sequencing was performed with Illumina technology and data were analyzed using ResFinder, PubMLST, and the CARD database. In this collection, resistance varied from 4.0% for colistin to 58.8% for ciprofloxacin. Acquired antibiotic resistance genes were found in 17.1% of the isolates, involving six different genes, but could not explain the majority of resistance. Single amino acid changes often did not lead to Minimal Inhibitory Concentrations (MICs) above the EUCAST susceptibility breakpoint. Determining the contribution of each amino acid change to clinical resistance was complicated by the large number of described changes found, the often low frequency of these changes and the high variability of the proteins involved. In particular, the diversity among OXA-ß-lactamases was large; despite over 900 OXA-types in the database, more than half of the variants in the isolate set were undescribed. Resistant isolates frequently had two or more amino acid changes. Four amino acid changes possibly related to β-lactam resistance were more common: two in AmpC (V239A and V356I) and two in PBP3 (R153S and R504C), three of which occurred more often in resistant isolates. Ciprofloxacin resistance could be linked to alterations in GyrA (in particular T82I and D87N) and to loss of or changes in MexZ. AmpD, NfxB, and PmrA, which are associated with resistance, were not detected in similar percentages of resistant and susceptible isolate. It can be concluded that frequently multiple mechanisms make a partial contribution to antibiotic resistance in this set of isolates.

## Introduction

*Pseudomonas aeruginosa* is an opportunistic pathogen that may cause chronic and life-threatening infections in patients with (chronic) lung conditions, such as cystic fibrosis (CF). Treatment of such infections is complex, as *P. aeruginosa* is intrinsically resistant to many antibiotics and may acquire resistance to (all) others. Resistance development often occurs during exposure to antimicrobials.

The genetics of antibiotic resistance in *P. aeruginosa* are highly complex. Resistance may be caused by acquisition of resistance genes, such as carbapenemases and aminoglycoside-modifying enzymes, but also by resistance mutations, variation in regulatory sequences and proteins, and combinations of these [1–3]. Resistance development under antibiotic therapy is often the result of upregulation of intrinsic ß-lactamases, in particular AmpC, or efflux pumps. Furthermore, deletions of porins, in particular OprD, may decrease susceptibility to different antibiotics.

*P. aeruginosa* strains harbour an intrinsic AmpC-ß-lactamase, normally expressed at low levels. Amino acid changes in the AmpC enzyme may result in altered ß-lactam specificity or reduced susceptibility to ß-lactamase inhibitors [4–7]. 1,6-anhydromuropeptides, the breakdown products that result from cell wall modifications, are inducers of AmpC production, by binding to AmpR, which regulates many processes in *P. aeruginosa* [8,9]. Furthermore, AmpC production may be upregulated by inactivation of the enzyme AmpD [10,11], and by lower expression of penicillin-binding protein 4 (PBP4) or mutations in its coding gene *dacB* [12–15].

*P. aeruginosa* has four main efflux pumps that may be involved in antibiotic resistance to different antibiotic classes: MexAB-OprM, MexCD-OprJ, MexEF-OprN and Mex XY-OprM. MexAB-OprM is the main efflux pump involved in antibiotic resistance; its overexpression may mediate resistance to ciprofloxacin, cefepime, ceftazidime, ceftazidime/avibactam, ciprofloxacin, piperacillin/tazobactam, meropenem and aztreonam. It is constitutively expressed in lab strains [16–18]. Its expression is controlled by multiple factors, including MexR, NalD, ArmA and NalC, which in part interact with each other [19–27]. MexCD-OprJ expression is controlled by the repressors NfxB and EsrC [28–31]. This efflux pump may expel ciprofloxacin, amikacin, cefepime, piperacillin and meropenem. It responds to agents imposing stress, but the natural inducer is not yet known [30,32,33]. MexEF-OprN, which excretes ciprofloxacin and meropenem, is controlled by MexT via the regulator MexS, which inactivates MexT. Wild-type strains consistently had a non-functional MexT which results in detectable expression of MexEF-OprN. Either mutations or the deletion of a 98 bp insert change the inactive form of MexT to the active form [34,35]. MexS decreased expression of OprD. Finally, MexXY is associated with OprM to form an efflux pump that can make strains resistant to gentamicin, tobramycin, amikacin, ciprofloxacin, cefepime, and meropenem. It is produced at very low levels in lab strains, but when upregulated contributes to antibiotic resistance [36]. Up-regulation can be induced by the presence of antibiotics [21,37–41]. MexXY is controlled by MexZ, and also regulated by the ParRS two component system [42,43].

Many amino acid changes associated with antibiotic resistance have been described (S1 Table). However, the complexity of the mechanisms involved often makes it difficult to assign their exact contribution to decreased antibiotic susceptibility of isolates, or to predict the phenotypic resistance pattern of an isolate based on DNA sequencing results. In this study, the whole genome sequences of 497 *P. aeruginosa* isolates obtained from patients with CF or other chronic pulmonary conditions were analyzed for known genetic resistance determinants to 12 commonly used antibiotics; these determinants were then compared to results of phenotypic resistance testing. Hereby, the aim was to establish correlations and to assess to what extent bacterial genetics predict resistance in this population.

## Materials and methods

### The study

The strains were collected for the iABC-project, in which novel antibiotics for CF and bronchiectasis were being developed. A total of 497 isolates were included in the study. These isolates were recovered between 2002 and 2016 from respiratory samples of CF (n = 412) and diverse chronic pulmonary conditions, in particular bronchiectasis (n = 85). The isolates were collected in four countries: Australia (n = 23), United Kingdom (n = 94), Spain (n = 139), and the Netherlands (n = 241). Most isolates were unique for the patient concerned, but from a subset of patients two or three isolates with different morphologies were included. Furthermore, there were two patients from whom 5 and 6 isolates with changing morphotypes were collected, over periods of five and four years respectively. All isolates were confirmed as *P. aeruginosa* by matrix-assisted laser desorption/ionization time of flight mass spectrometry (MALDI-TOF/MS, Bruker) prior to performing whole genome sequencing (WGS) [44].

This study did not involve any human participants, human tissue or patient data, and therefore did not require ethical approval as isolates were obtained before 2016, when Institutional Review Board approval or waivers could not be obtained. Patient data were not used. Nevertheless, isolates were collected in compliance with the Declaration of Helsinki ICH-GCP, the Declaration of Taipei regarding Health Databases and Biobanks, and with local and European regulations for collection and handling of patient data. Since the study concerned retrospectively collected anonymized patient data and bacterial isolates, informed consent at the individual patient level was not required for this study. In addition, the Spanish and UK isolates were collected in accordance with their local ethics guidelines and described before [44]. In the Netherlands, use and analysis of bacterial isolates with anonymized patient data does not require approval from Institutional Review Boards/Ethics Committees [https://wetten.overheid.nl/BWBR0009408/2021-07-01].

Experimental protocols did not need approval by a named institutional and/or licensing committee because analysis of bacterial strains was not subject to approval.

Informed consent was not required by local laws because only bacterial strains were used.

### Antimicrobial susceptibility testing

Minimum inhibitory concentrations (MICs) were determined previously by the standard ISO broth microdilution method with frozen panels (Trek Diagnostic Systems, Westlake, OH) using EUCAST methodology [45,44]. Of note, EUCAST resistance breakpoints are generally set at the epidemiological cut-off (ECOFF) that distinguishes wild-type strains (in terms of antibiotic susceptibility) from non-wildtype strains [45]. Both the susceptible (S) and the susceptible with increased exposure (I) categories were analyzed as being susceptible, with the exception of meropenem, where these categories exist next to one another and were therefore analyzed separately.

### Whole genome sequencing

Bacterial DNA was purified using the QIAcube with the DNeasy Blood & Tissue kit with the enzymatic lysis protocol (Qiagen, Carlsbad, CA) and used to prepare a library for sequencing with the MiSeq or Nextseq (Illumina, San Diego, CA) platforms, using the Nextera XT library prep kit (Illumina). Contigs were assembled with SPAdes genome assembler v.3.6.2 [46]. The assembled contigs were analyzed for the presence of resistance genes by ResFinder from the Center for Genomic Epidemiology (DTU, Copenhagen, Denmark) [47,48]. *bla*_OXA_-types were confirmed using the CARD database [49]. Multi-locus Sequence Typing (MLST) was performed using PubMLST using the scheme for *P. aeruginosa* [50]. Novel alleles and sequence types (ST) were submitted to the database.

Thirty-five proteins in which mutations and deletions are frequently associated with antibiotic resistance were analyzed. These included intrinsic ß-lactamases, efflux pumps, two-component systems, and proteins involved in household functions: AmpC-ß-lactamases, OXA-ß-lactamases, AmpR, AmpD, PBP3, PBP4, MexAB, MexCD, MexEF, MexR, MexS, MexT, MexZ, NalC, NalD, OprD, OpRJ, OprM, OprN, PhoQ, PmrA-PmrB, NfxB, gyrase A, LptD, OpmD, gyrase B, and elongation factor G (*fusA1*). The amino acid variation in each protein was assessed by comparison to strain PAO1 as a reference (accession number GCF_000006765.1).

WGS results were compared between resistant and susceptible isolates, and reported in a descriptive manner, without statistical analysis.

### Availability of data and materials

The sequence data have been submitted to GenBank (BioProject ID: PRJNA530912 available at https://dataview.ncbi.nlm.nih.gov/object/PRJNA530912). For clarity the identifications used in this manuscript refer to last 6 unique digits of the strain and sample name in the database.

## Results and discussion

### Susceptibility of the isolates

Applying the EUCAST 2024 breakpoints, susceptibility of the isolates was as follows: amikacin (S) 72.2%, tobramycin (S) 74.6%, ciprofloxacin (I) 41.2%, colistin (S) 96.0%, imipenem (I) 69.8%, meropenem (S + I) 78.5% + 10.7%, ceftazidime (I) 79.7%, ceftazidime/avibactam (S) 94.2%, aztreonam (I) 76.1%, ceftolozane/tazobactam (S) 95.8%, piperacillin/tazobactam (I) 80.5%, and cefepime (I) 76.3%.

### Genome characteristics

Sequencing and assembly of the whole genomes of the isolates resulted in an average of 156 contigs larger than 1000 bp, and an average coverage of 59x, indicating high quality sequences. The genome size was approximately 6.46 MB, with an average GC content of 66.32%. The isolates belonged to 206 different STs including 27 novel types. Forty-two novel alleles were found: 6 for *ascA*, 9 for *aroE*, 7 for *trpE*, 9 for *mutL*, 4 for *nuoD*, 6 for *ppsA*, and 1 for *guaA*. One isolate lacked *nuoD* (555916) and 2 had a deletion in *trpE* (isolates 538187 and 538188). The STs with the highest number of isolates were ST406 (n = 30), ST146 (n = 28), ST27 (n = 18), ST508 (n = 12), ST17 and ST253 (n = 11), and ST155 and ST395 (n = 10 for both). Overall, 120 STs were represented by a single isolate and all other STs were represented by 2–9 isolates (S2 Table and S3 Table). The large number of new STs and alleles suggest that a large part of the variation in the population of *P. aeruginosa* is not yet covered by the PubMLST database.

Despite good coverage of the genome, and finding less than 1% incomplete genes, for some isolates, the genes for some proteins were found absent. The absence of some proteins may be the result of sequencing and/or assembly artefacts, e.g., due to low coverage, but this is not likely, as coverage was good and also the total number of incomplete genes was below 1% (Table 1). However, for MexX 43 incomplete genes were found. Further analysis showed that parts of these genes were frequently present on 2 contigs. Also, with Sanger sequencing it was not possible to obtain a complete gene sequence, which suggests that besides the gene for MexX a similar sequence is present.

**Table 1:** Variability of commonly present proteins involved in resistance in 497 *Pseudomonas aeruginosa* isolates, recovered from chronic pulmonary infections.

protein	length (number of aa)	number of variants	ratio variants\length	number aa positions changed	% positions changed	isolates with truncated proteins	isolates without the protein
AmpC	397	79	0.20	106	26.7	0	0
AmpDß-lactamase	188	17	0.09	25	13.9	0	399
AmpR	300	37	0.12	41	13.0	1	1
EF-G*	646	85	0.13	74	11.5	0	0
GyrA	923	80	0.09	53	5.7	0	0
GyrB	806	44	0.05	39	4.8	0	0
LptD	924	95	0.10	166	18.0	5	0
MexA	383	65	0.17	36	9.4	21	12
MexB	1046	105	0.10	64	6.1	25	4
MexC	387	41	0.11	44	11.4	3	45
MexD	1043	65	0.06	115	11	3	1
MexE	414	51	0.12	44	10.6	7	9
MexF	1062	67	0.06	60	5.6	0	3
MexR	147	36	0.24	19	12.9	0	2
MexS	339	80	0.24	68	20.0	2	14
MexT	310	59	0.19	41	13.2	5	5
MexX	1045	143	0.14	142	14.4	7	8
MexY	396	47	0.12	57	14.4	3	9
MexZ	186	153	0.82	69	37.1	1	68
NalC	213	36	0.17	38	17.8	0	0
NalD	212	28	0.13	21	9.9	2	0
NfxB	187	37	0.20	54	28.9	2	199
OpmD	487	71	0.15	98	20.1	1	3
OprD	441	59	0.13	54	12.2	16	11
OprJ	479	52	0.11	79	16.5	1	4
OprM	485	39	0.10	36	7.4	5	4
OprN	472	53	0.11	52	11.0	1	10
OXA-ß-lactamase	262	48	0.18	63	24.0	0	0
ParC	754	57	0.08	55	7.3	0	0
ParE	629	40	0.06	40	6.4	0	0
PBP3	579	59	0.10	62	10.7	0	1
PBP4	476	57	0.12	32	6.7	26	1
PhoQ	590	32	0.05	40	6.8	3	0
PmrA	221	15	0.07	14	6.3	0	124
PmrB	477	85	0.18	89	18.7	6	0

* Elongation factor G

### Variability of commonly present proteins involved in antibiotic resistance

For the 35 analyzed proteins, the number of amino sequence variants per protein ranged from 15 (PmrA) to 153 (MexZ). However, the length (number of amino acids) of the proteins also varied and larger proteins allow more amino acid changes. When corrected for length by calculating the ratio of the number of variants to the length, the ratio varied nearly sixteen-fold (0.05 to 0.82) for GyrB and PhoQ versus MexZ, respectively (Table 1). The number of amino acids changed varied from 14 (PmrA) to 166 (LptD) when corrected for the length the percentages of amino acid positions changed ranged from 4.8 to 37.1 (GyrB and MexZ, respectively) (Table 1). In conclusion: the number of amino acid changes varies depending on the protein, but is not dependent on the protein length, indicating a larger sequence flexibility for some proteins, which makes the identification of relevant amino acid changes complex.

Most of the described amino acid changes were found in low numbers of isolates. Exceptions were the mutations in AmpC leading to V239A (n = 15) and V356I (n = 44), which were found in a large diversity of STs, excluding clonal expansion and possibly indicating that these mutations were driven by environmental conditions and/or antibiotic exposure. V239A was correlated with resistance to multiple ß-lactam antibiotics. The V356I change in AmpC was found partly in clustered isolates: five isolates belonged to ST1203 and eight to ST1225. In the latter group, 3 isolates were from one patient and two from another.

Two other exceptions were the mutations in the gene coding for PBP3, leading to R153S (n = 22) and R504C (n = 34). These were universally more common in isolates with higher MICs for ß-lactam antibiotics. In the case of meropenem, the R153S change was most often found in the increased exposure category, i.e., more than in the resistant and the susceptible categories: the mutation would lead to a higher MIC vs wild-type, but not to an MIC above the clinical breakpoint for resistance (R). However, all isolates with a R153S change also had a R504C change; with one exception these isolates belonged to a large cluster of ST146, the Liverpool Epidemic Strain, all of which were isolated from different patients in Northern Ireland. Seven of the isolates with only a R504 change were also ST146 [51]. This makes their importance for ß-lactam resistance questionable: the frequency of corresponding mutations was more likely the result of clonal expansion, than of induction due to antibiotic exposure.

Differences in the putative start of open reading frames resulting in longer proteins than previously described were observed. These proteins started with the usual methionine. Nearly all (n = 477, 97%) open reading frames for PhoQ were 590 amino acids long. However, based on The Pseudomonas Genome Database, Owusu-Anim and Kwon and BLAST to GenBank, this protein is 448 amino acids [52,53]. The sequence in the Pseudomonas Genome Database starts at position 143 of sequences used here. This position is a valine (V), whereas in the database it starts with a methionine (M). Also, in the original publication describing the presence of PhoP-PhoQ in *P. aeruginosa* a V is used as first amino acid, but tracing back references the evidence for this is unclear [54]. At position 9 of PhoQ, an M is present in both strain PAO1 and the most common sequence in this study and may be used as a start. It is therefore possible that the translation starts at a different position from the one currently reported in the database used.

A similar observation was made for PmrB. Nearly all the PmrB open reading frames in this collection were longer than the 477 amino acid sequence in the Pseudomonas Genome Database: 527 (n = 399, 80.3%), 733 (n = 33, 6.6%), or 981 (n = 38, 7.6%) amino acids [53]. Only 1 isolate had a 477 amino acid sequence and 8 were shorter, 10 were between 477 and 549 amino acids, 3724 amino acids, 3 longer or equal to 841 amino acids, and for two isolates, PmrB was not detected [53].

Some proteins appeared to be non-essential, as they were absent in many isolates. Notably, AmpD, NfxB, and PmrA were not detected in 399, 217, and 124 isolates respectively (Table 1). MexZ was absent in all 21 ST146 isolates. For 15 of the 35 proteins studied, none of the amino acid changes associated with resistance were found in the collection used here.

### Acquired antibiotic resistance genes

Acquired antibiotic resistance genes were found in 17.1% (n = 85) of the isolates, involving six different genes: *crpP*, *aph(3”)-IIb*, *catB7*, *aadA6*, *aadA7*, and *sul1* as well as *fosA* which is present in some isolates, but is chromosomally located (Table 2) [55]. The most common pattern was the presence of *crpP* (ciprofloxacin resistance), *fosA* (fosfomycin resistance), catB7 (chloramphenicol resistance and *aph(3”)-IIb* (streptomycin resistance). The aminoglycoside adenylyltransferases *aadA6* (n = 6) and *aadA7* (n = 1), which confer resistance to streptomycin, were always found together with *sul1* (sulfonamide resistance), which indicates their presence in a class-I integron [56]. *aph(3”)-IIb* was found in 84 of the isolates [57]. *CatB7* was present in 80 isolates, *fosA* in all 85 isolates, and *crpP* in 50 isolates. All 50 isolates carrying the *crpP* gene were resistant to ciprofloxacin according to the EUCAST breakpoints; 29 of these isolates were in the EUCAST increased exposure category (1 mg/L) and 21 in the resistant category (≥2 mg/L). Other acquired resistance genes were not detected. The majority of antibiotic resistance observed in the isolates could therefore not be explained by acquisition of resistance genes.

**Table 2:** Acquired resistance genes.

crpP	fosA	catB7	aph(3“)-IIb	other R gene	other R gene	number/% of isolates
–	+	–	+	–	–	3/0.6
–	+	+	+	–	–	31/6.2
+	+	–	+	–	–	1/0.2
+	+	+	–	–	–	1/0.2
+	+	+	+	–	–	41/8.2
+	+	+	+	–	–	1/0.2
–	+	–	+	aadA6	sul1	1/0.2
+	+	+	+	aadA6	sul1	5/1.0
+	+	+	+	aadA7	sul1	1/0.2

### Aminoglycoside resistance

Overall, 25.4% (n = 126) of the isolates were resistant tobramycin and 27.8% (n = 138) to amikacin. Aminoglycoside resistance may be caused by amino acid changes in EF-G (elongation factor G), PhoQ, MexZ and MexXY-OprM. The amino acid changes found in this collection are described in Table 3. EF-G amino acid changes Y555C, A555E, and D588G were mostly found in aminoglycoside-resistant isolates, but this was in very low numbers, and the mutations could also occur in susceptible isolates. The amino acid change Y85F in PhoQ, which has been described to result in aminoglycoside resistance, was present in 4 isolates, of which 2 were susceptible to both tobramycin and amikacin, and one was susceptible to amikacin only. The V260G was present in a single isolate, which was resistant to aminoglycosides.

**Table 3:** Amino acid changes in proteins involved in aminoglycoside resistance in resistant isolates compared to susceptible isolates.

		amikacin	amikacin	tobramycin	tobramycin
**protein**	**change**	**R** **n = 138, 27.8%** **(number/%)**	**S** **n = 359, 72.2%** **(number/%)**	**R** **n = 126, 25.4** **(number/%)**	**S** **n = 371, 74.6** **(number/%)**
EF-G*	M461V	1/0.7	1/0.3	1/0.8	1/0.3
	Y552C	4/2.9	2/0.6	4/3.2	2/0.5
	A555E	4/2.9	0/0.0	3/2.4	1/0.3
	D588G	4/2.9	0/0.0	3/2.4	1/0.3
	T671A	2/1.4	1/0.3	2/1.6	1/0.3
MexZ	G46S	1/0.7	0/0.0	1/0.8	0/0.0
	G50D	1/0.7	0/0.0	1/0.8	0/0.0
	Y52N	2/1.4	0/0.0	2/1.6	0/0.0
PhoQ	Y85F	1/0.7	3/0.8	2/1.6	2/0.5
	V260G	1/0.7	0/0.0	1/0.8	0/0.0

* Elongation factor G

Changes at 9 amino acid positions in MexZ have been described to result in aminoglycoside resistance, 3 of which were found in the collection used here, all in resistant isolates. MexZ was absent in 31 (18.5%) of the resistant isolates, compared to 21 (7.0%) of the susceptible isolates. In the collection used here, 153 variants of MexZ were present, many of which were the result of insertions, deletions and changes in the open reading frame (Table 1). The low numbers of isolates for each variant made it impossible to draw conclusions with respect to their impact on aminoglycoside resistance, with the exception of a protein variant with a start 7 amino acids earlier and out-of-frame until E20. This variant was present in 13.7% of the resistant isolates and 2.1% of the susceptible isolates, suggesting a possible contribution to resistance.

In 29 isolates with aminoglycoside resistance, no previously associated amino acid change was present, nor was any insertion or deletion.

### Ciprofloxacin resistance

A total of 292 isolates (58.8%) were resistant to ciprofloxacin; Table 4 describes the associated amino acid changes found. Ciprofloxacin resistance can be mediated by amino acid changes in the targeted enzymes DNA gyrase (GyrA and GyrB) and topoisomerase (ParC and ParE), with changes in GyrA being the most common mechanism in *P. aeruginosa* [58]. *In vitro* studies indicate that mutations in *gyrA* are a prerequisite for development of ciprofloxacin resistance, with mutations in *parC/E*, *nfxB* and *rnfC* further enhancing MICs [59].

**Table 4:** Amino acid changes in proteins involved in ciprofloxacin resistance in resistant isolates compared to susceptible isolates.

		Change present in (number/ %)	
protein	change	R n = 292, 58.8% (number/%)	S* n = 205, 41.2% (number/%)
GyrA	T83A	7/2.4	0/0.0
	T83I	91/31.2	11/5.4
	D87G	3/1.0	5/2.4
	D87H	2/0.7	0/0.0
	D87N	33/11.3	1/0.5
	D87V	2/0.7	0/0.0
	D87Y	6/2.1	0/0.0
GyrB	S466F	63/21.6	12/5.9
	E468D	8/2.7	0/0.0
ParC	S87L.	9/3.1	0/0.0
	S87W.	2/0.7	0/0.0
ParE	S457G	2/0.7	0/0.0
	A473V	6/2.1	1/0.5
MexS	C245G	2/0.7	0/0.0
	F253L	3/1.0	0/0.0
MexZ	A38V	2/0.7	1/0.5
	G50D	1/0.3	0/0.0
	Y52N	2/0.7	0/0.0
	G195E	2/0.7	0/0.0
NalC	E153D	1/0.3	0/0.0
	A186T	23/7.8	18/8.8
NalD	T43I	1/0.3	0/0.0
	T188A	1/0.3	11/5.4
Any of the above		207/70.8	44/21.5

Overall, 149 isolates had amino acid changes in GyrA associated with fluoroquinolone resistance; 89% of these isolates had the resistant phenotype. Two amino acid changes in particular were associated with a resistant phenotype, namely T82I (found in 31.2% resistant vs 5.4% of susceptible isolates) and D87N (11.3% vs 0.5%) (Table 4). For GyrB, 2 of the 8 described amino acid changes were found; 85.5% of the corresponding isolates had a resistant phenotype. 11 isolates had a described resistance amino acid change in ParC, all at position 87; these isolates were all ciprofloxacin resistant. Eight of the nine (89%) isolates with a described amino acid change in ParE were resistant to ciprofloxacin. 30 isolates had a change in more than one of the prior four amino acid genes; these isolates were all resistant. One isolate had described amino acid changes in all 4 genes. Mutations in these genes could explain resistance in 207 of 292 (70.9%) ciprofloxacin resistant isolates.

Alternative mechanisms for resistance are changes in the efflux pumps or their regulators, leading to enhanced efflux. This concerns the pump MexAB-OprM with its regulators NalC, NalD, and MexR, and the pumps MexXYZ-OprM and MexCD-OprJ with their regulator NfxB. Such amino acid changes were indeed found in the regulators NalC, NalD, MexR, MexS, and MexZ, but generally in low numbers; some of these changes were even more frequently present in susceptible isolates, such as NalD T188A (0.3% of resistant vs 5.4% of susceptible) (Table 4). The deletion of the four amino acids TPVE after L123Q in MexR may be associated with ciprofloxacin resistance as it was present in 9 (3.1%) resistant isolates and absent in susceptible isolates. Absence of MexZ was 3 times more frequent among resistant isolates compared to susceptible isolates (15.1% vs 4.9%). Also, a protein variant with a start 7 amino acids earlier and out-of-frame until E20 of MexZ was more frequently present among resistant isolates (9.2% vs 1.5%).

To assess whether a combination of multiple amino acid changes in the previous genes is more likely to predict resistance, the number of isolates with multiple described amino acid changes in all proteins involved in ciprofloxacin resistance and the number of isolates with only a single amino acid change was determined. The ratio of the number of isolates with multiple described amino acid changes in any of the proteins studied versus a single amino acid change was 44 vs 167 (ratio 0.26) for ciprofloxacin-resistant isolates and 6 vs 42 (ratio 0.14) for the susceptible group. This suggests that multiple amino changes may be required to obtain resistance.

### Colistin resistance

Twenty isolates (4.0%) were resistant to colistin. The two-component regulatory systems PhoP-PhoQ and PmrA-PmrB are involved in polymyxin B and colistin (polymyxin E) resistance [60]. The genes for this two-component system form an operon together with *oprH*, which encodes an outer membrane porin protein. The system regulates genes involved in bacterial virulence, transmembrane transport, lipopolysaccharide (LPS) modification, and resistance against antibiotics including antimicrobial peptides [61–63]. PhoQ acts as sensor and PhoP as the response regulatory protein [64]. A defect in *phoQ* results in constitutive expression of PhoP-regulated genes [65]. Polymyxin binds to LPS, but addition of 4-amino-4-deoxy-L-arabinose through enzymes of the *arnHIJKLM* operon reduces negative charge and thereby binding of polymyxin [66]. This operon is regulated by PhoP. The PhoP-PhoQ system also regulates *pagP* that contributes palmitoylation of LPS, which may lead to resistance [67]. However, only two isolates in the collection used here had a described amino acid change in PhoQ and both were susceptible.

PmrAB is also a 2-component system that directly regulates the *arnHIJKLM* operon [68,69]. The systems respond to Mg^2+^ limiting conditions which results in overexpression of the *arnHIJKLM* operon and decreased susceptibility to polymyxins. Nine isolates had a described amino acid change in PmrB, of which only one was resistant to colistin (n = 4: A248T; n = 2: M292I; n = 1: D45N, D47A, and G188S) [52,68–71]. In 118 isolates, PmrA could not be detected, but only 5 of these were colistin-resistant.

Expression of the *arnHIJKLM* operon is also regulated by the two-component systems BqsR-BqsS, CprR-CprS, and ParR-ParS, but their role in resistance seems more limited [68,72,73].

### Carbapenem resistance

Resistance correlated well between imipenem and meropenem: 51 of the 54 meropenem-resistant isolates were also resistant to imipenem, but 51 of the 391 meropenem-susceptible isolates were imipenem-resistant.

Carbapenem resistance may be mediated by amino acid changes in OXA- and AmpC-ß-lactamases including the regulators AmpR and AmpD and over-expression of AmpC. The third chromosomal ß-lactamase described in *P. aeruginosa* is PIB-1, a Zn^2+^-dependent imipenase. It is considered part of the core genome, but its importance in clinical settings is not clear [74,75]. OprD, a porin, plays a role in imipenem resistance. Meropenem resistance can be mediated via efflux by MexAB-OprM, which is regulated by NalC, NalD, and MexR, by efflux through MexXY-OprM with MexZ as regulator (repressor), and by efflux through MexCD-OprJ which has NfxB as the main regulator.

OXA-family ß-lactamases can contribute to carbapenem resistance, but in most cases additional mechanisms are required to achieve an MIC above the clinical breakpoint [76,77]. The presence of OXA-types 4, 794, 795 or 824 has been reported to result in carbapenem resistance, but none of these types were present in the collection used here and comparison of the sequences of these ß-lactamases with those in the collection used here showed large differences (approximately 24% identity with OXA-4 and approximately 30% identity with OXA-794, OXA-795, and OXA-824) [78]. Some OXA family genes are part of the core genome of *P. aeruginosa*. Forty-eight different OXA-types were present in the collection used here, but only 14 of these were present in the Comprehensive Antibiotic Resistance Database (CARD) [49]. Of the undescribed types, 21 had a single amino acid change compared to already described types, 1 type had a deletion, 1 was partially out-of-frame and the other 11 types had multiple amino acid changes (S4 Table). OXA-type ß-lactamases present in the collection used here did not appear to result in carbapenem resistance. OXA-50 with additional F6L and F168L amino acid changes were more frequently present among meropenem-resistant isolates (27.8% vs 4.1%) as were OXA-486 (16.7% vs 11.3%) and OXA-487 (14.8% vs 6.3%), suggesting they may contribute to carbapenem resistance (S4 table). OXA-types among imipenem-susceptible and resistant isolates showed no additional major differences.

In AmpC, the V239A amino acid change was found more frequently in resistant compared to susceptible isolates (n = 11/6.6% vs n = 4/1.2% and n = 1/1.9% vs n = 7/13.0% for imipenem and meropenem, respectively) (Table 5). However, the amino acid change V536I was more common in imipenem-susceptible isolates (10.4% vs 4.8%); this change was absent in meropenem-susceptible isolates, but present in 10.5% and 5.6% of the increased exposure category and meropenem-resistant isolates, respectively (Table 5).

**Table 5:** Amino acid changes in proteins involved in imipenem en meropenem resistance in resistant isolates compared to susceptible isolates.

		iminpenem	imipenem	meropenem	meropenem	meropenem
protein	change	R n = 150, 30.2% (number/%)	S n = 347, 69.8% (number/%	R n = 54, 10.9% (number/%)	S n = 391, 78.7% (number/%)	I n = 53, 10.7% (number/%)
AmpC	H215Y	1/0.6	0/0.0	1/1.9	0/0.0	0/0.0
	V239A	11/6.6	4/1.2	7/13.0	7/1.8	1/1.9
	G242S	2/1.2	0/0.0	1/1.9	0/0.0	1/1.9
	D245G	1/0.6	0/0.0	1/1.9	0/0.0	0/0.0
	N347S	2/1.2	0/0.0	2/3.7	0/0.0	0/0.0
	V356I	8/4.8	36/10.4	3/5.6	41/10.5	0/0.0
	N373I	2/1.2	0/0.0	1/1.9	0/0.0	1/1.9
AmpD	A136V	1/0.6	1/0.3	0/0.0	2/0.5	0/0.0
	G148A	4/2.4	6/1.7	2/3.7	7/1.8	1/1.9
	absent	120/80.0	279/80.4	43/79.6	356/91.0	0/0.0
MexB	none	n.a.*	n.a.	-/-	-/-	-/-
MexS	F253L	n.a.	n.a.	0/0.0	0/0.0	1/1.9
MexZ	G50D	n.a.	n.a.	1/1.9	0/0.0	0/0.0
	Y52	n.a.	n.a.	0/0.0	1/0.3	1/1.9
	G145E	n.a.	n.a.	1/1.9	1/0.3	0/0.0
	alternative start**	n.a.	n.a.	14/25.6	10/2.6	0/0.0
	absent	n.a.	n.a.	6/12.3	48/10.8	0/0.0
NalC	A145V	n.a.	n.a.	2/3.7	16/4.1	0/0.0
	A186T	n.a.	n.a.	6/11.1	29/7.4	6/11.3
NalD	T188A	n.a.	n.a.	0/0.0	0/0.0	2/3.8
NfxB	none	n.a.	n.a.	0/0.0	0/0.0	0/0.0
PBP3	A28T	1/0.6	0/0.0	0/0.0	0/0.0	1/1.9
	G63S	4/2.4	0/0.0	3/5.6	0/0.0	1/1.9
	R153S	14/8.4	8/2.3	3/5.6	12/3.1	7/13.2
	N242S	1/0.6	0/0.0	1/1.9	0/0.0	0/0.0
	G243S	1/0.6	4/1.2	0/0.0	4/1.0	1/1.9
	A244T	2/1.2	0/0.0	2/3.7	0/0.0	0/0.0
	N283S	1/0.6	0/0.0	0/0.0	0/0.0	1/1.9
	A454V	1/0.6	0/0.0	1/1.9	0/0.0	0/0.0
	R504C	23/13.9	11/3.2	6/11.1	16/4.1	12/22.6
	F507L	2/1.2	1/0.3	2/3.7	1/0.3	0/0.0
	P527S	3/1.8	1/0.3	2/3.7	2/0.5	0/0.0
	F533L	1/0.6	2/0.6	3/5.6	0/0.0	1/1.9
PBP4	none	-/-	-/-	-/-	-/-	-/-
OprD	F170L	11/7.3	74/21.3	2/3.7	81/20.7	2/3.8
	G316P	3/5.6	0/0.0	3/5.6	0/0.0	0/0.0

* not analyzed; protein not reported to be involved in resistance

** start 7 amino acids earlier and out-of-frame until E20

Uptake of carbapenems by *P. aeruginosa* occurs mainly via the porin protein OprD, and premature stops of OprD are associated with carbapenem resistance [79–82]. Of the isolates in which the OprD gene was truncated (n = 18) or not detected (n = 1), 7 were meropenem-resistant and 14 were imipenem-resistant. The in frame deletion _312_GSGAGG_317_ in OprD has been reported to result in resistance [83]; all 6 isolates in the collection used here that missed this sequence due to premature stop-codons were imipenem-resistant, but only one was meropenem-resistant. The F170L amino acid change in OprD has been reported to contribute to carbapenem resistance, but it was more frequently present in susceptible than in resistant isolates (21.3% vs 7.3% and 3.8% vs 3.7% for imipenem and meropenem, respectively), but it was present in 20.7% of the meropenem increased exposure isolates (Table 4). Also, alterations of which shorten loop L7 (one of the external amino acid sequences that connect trans-membrane sequences of OprD), have been related to increased susceptibility to meropenem [83–86]. A completely different L7 sequence was observed in 464 isolates (93.4%) compared to strain PAO1: _372_VDSSSSYAGL_383_ vs _372_MSDNNVGYKNYG_383_, respectively. Absence of this region due to premature stop codons was not associated with resistance to either imipenem or meropenem.

Efflux pump MexB can excrete β-lactams including some β-lactamase inhibitors and carbapenems (but not imipenem), out of the bacterial cell. The protein variant of MexZ with a start 7 amino acids earlier and out-of-frame until E20 may contribute to meropenem resistance as 25.9% of the resistant isolates had this variant compared to 2.6% of the susceptible and increased exposure isolates (Table 5).

Although target mutations in PBPs are uncommon as resistance mechanisms in gram-negative bacteria, amino acid changes in PBP3 (*ftsI*) may contribute to ß-lactam resistance in *P. aeruginosa* [87]. Several amino acid changes associated with carbapenem resistance have been identified in PBP3. Of these, R504C was more frequently found in resistant isolates compared to susceptible isolates (imipenem 13.9% vs 3.2%), but this change was present in 11.1% of the meropenem-resistant isolates, 4.1% of the susceptible with increased exposures isolates, and 22.6% of the susceptible isolates (Table 5).

The number of isolates with multiple described amino acid changes in any of the proteins studied versus a single amino acid change was 15 vs 17 (ratio 0.88) for meropenem-resistant isolates and 44 vs 120 (ratio 0.37) for the susceptible group. For imipenem these numbers were 28 vs 45 isolates (ratio 0.62) and 21 vs 98 isolates (ratio 0.21), respectively. This suggests that often multiple amino changes are required to obtain resistance.

### Resistance to ceftazidime, ceftazidime/avibactam, and aztreonam

Resistance to aztreonam, ceftazidime, and ceftazidime/avibactam can result from amino acid changes in the OXA and AmpC ß-lactamases, from alterations in the AmpC regulators AmpR and AmpD, from changes in the penicillin binding proteins PBP3 and PBP4, and from changes in efflux pump MexAB-OprM and its regulators, NalC, NalD, MexR.

The V239A substitution in AmpC was more frequently present in resistant isolates versus susceptible isolates (ceftazidime: 8.9% vs 1.5%; ceftazidime-avibactam: 20.1% vs 1.9%; aztreonam: 7.6% vs 1.6%) (Table 6). In contrast, the V356I amino acid change was found more often in ceftazidime- and ceftazidime/avibactam-susceptible isolates (9.3% and 9.0% respectively) than resistant isolates (6.9% and 6.9% respectively) (Table 6). In 80.3% of the isolates, the AmpD gene could not be detected; this percentage was only slightly higher in isolates resistant to ceftazidime and/or aztreonam than in susceptible isolates. Additionally, in the remaining 98 isolates, 1 AmpD sequence was out-of-frame after A85, likely resulting in a non-functional protein, and 10 isolates had the G148A amino acid change, implicated in AmpC upregulation (Table 6).

**Table 6:** Amino acid changes in proteins involved in ceftazidime, ceftazidime-avibactam, and aztreonam resistance in resistant isolates compared to susceptible isolates.

Protein	Change	Ceftazidime		Ceftazidime-avibactam		Aztreonam	
		R n = 101, 20.3% (number/%)	I n = 396, 79.7% (number/%)	R N = 29, 5.8% (number/%)	S n = 468, 94,2% (number/%)	R n = 119, 23.9% (number/%)	I n = 378, 76.1% (number/%)
AmpC	H215Y	1/1.0	0/0.0	0/0.0	1/0.2	1/0.8	0/0.0
	V239A	9/8.9	6/1.5	6/20.1	9/1.9	9/7.6	6/1.6
	G242S	1/1.0	1/0.3	0/0.0	2/0.4	2/1.7	0/0.0
	D245G	1/1.0	0/0.0	1/3.4	0/0.0	1/0.8	0/0.0
	N347S	2/2.0	0/0.0	0/0.0	2/0.4	2/1.7	0/0.0
	V356I	7/6.9	37/9.3	2/6.9	42/9.0	12/10.1	32/8.5
	N373I	1/1.0	1/0.3	0/0.0	2/0.4	0/0.0	2/0.5
AmpD	G148A	2/2.0	8/2.0	1/3.4	9/1.9	2/1.7	8/2.1
	absent	90/93.8	309/78.0	23/79.3	376/80.3	102/85.7	297/77.7
MexB	none	-/-	-/-	-/-	-/-	-/-	-/-
MexS	C245L	n.a.	n.a.	n.a.	n.a.	2/1.7	1/0.3
	F233L	1/1.0	1/0.3	n.a.	n.a.	0/0.0	2/0.5
NalC	A186T	11/10.9	30/7.6	4/13.8	37/7.9	13/10.9	28/7.4
NalD	T34I	n.a.	n.a.	n.a.	n.a.	1/0.8	0/0.0
	T188A	n.a.	n.a.	n.a.	n.a.	1/0.8	1/0.3
PBP3	A28T	1/1.0	0/0.00	0/0.0	1/0.2	1/0.8	0/0.0
	G63S	4/4.0	0/0.0	1/3.4	3/0.6	3/2.5	1/0.3
	R153S	13/12.9	9/2.3	3/10.3	19/4.1	8/6.7	14/3.7
	N242S	1/1.0	0/0.00	0/0.00	1/0.2	1/0.8	0/0.0
	G243S	2/2.0	3/0.8	1/3.4	4/0.9	2/1.7	3/0.8
	A244T	2/2.0	0/0.0	1/3.4	1/0.2	2/1.7	0/0.0
	N283S	1/1.0	0/0.0	0/0.00	1/0.2	1/0.8	0/0.0
	R504C	18/17.8	16/4.0	7/24.1	27/5.8	13/10.9	21/5.6
	F507L	3/3.0	0/0.00	0/0.00	3/0.6	3/2.5	0/0.0
	P527S	1/1.0	3/0.8	1/3.4	3/0.6	4/3.4	0/0.0
	F533L	2/2.0	1/0.3	1/3.4	2/0.4	2/1.7	1/0.3
PBP4	none	-/-	-/-	-/-	-/-	-/-	-/-

The PBP3 amino acid changes R153S and R504C were more frequently found in resistant than in susceptible isolates (Table 6), but it should be noted that, with 1 exception, all isolates belonged to ST146. All 4 isolates with the P527S change, belonging to four different STs, were resistant to aztreonam (Table 6). None of the described PBPB4 amino acid changes associated with resistance to ceftazidime, ceftazidime-avibactam or aztreonam was found.

The A186T amino acid change in NalC was more common in resistant than in susceptible isolates with 10.9% vs 7.6%, 13.8% vs 7.9%, and 10.9% vs 7.4% for ceftazidime, ceftazidime/avibactam, and aztreonam, respectively (Table 6).

Multiple amino acid changes in proteins involved in resistance to ceftazidime, ceftazidime/avibactam, and aztreonam were more common in resistant than in susceptible isolates. The number of multiple to 1 amino acid change was 24 vs 30 (ratio 0.80) and 19 vs 88 (ratio 0.22) for resistant vs susceptible isolates for ceftazidime. For aztreonam these numbers were 9 vs 13 (ratio 0.69) and 21 vs 78 (ratio 0.27), respectively, and for ceftazidime/avibactam-resistant and susceptible isolates 9 vs 9 (ratio 1.0) and 36 vs 110 (ratio 0.33).

### Ceftolozane/tazobactam resistance

Amino acid changes in AmpC and its regulators, and in PBP4 can result in ceftolozane/tazobactam resistance. In AmpC (regulation) only the V239A amino acid change was clearly associated with resistance (28.5% of resistant isolates vs 1.9% of susceptible isolates). Absence of AmpD did not seem to contribute (Table 7). Amino acid changes in PBP3 were more often found in resistant isolates, in particular the mutations leading to the G243S and R504C alterations (Table 7). It should be noted that 25% of the resistant isolates had multiple described amino acid changes in the investigated proteins involved in ceftolozane/tazobactam resistance, but only 2.6% of the susceptible isolates. Only 7 isolates had an insertion/deletion in one of the proteins involved in resistance, which precludes definite conclusions about their contribution.

**Table 7:** Amino acid (aa) changes in proteins involved in cefepime resistance in resistant isolates compared to susceptible isolates.

protein	change	cefepime		piperacillin/ tazobactam		ceftolozane/ tazobactam	
		R n = 118/23.7% (number/%)	I n = 379/76.3% (number/%)	R n = 97/19.5% (number/%)	I n = 400/80.5% (number/%)	R n = 21/4.2% (number/%)	S n = 476/95,8% (number/%)
AmpC	H215Y	1/0.8	0/0.0	1/1.0	0/0.0	0/0.0	1/0.2
	V239A	10/8.5	5/1.3	8/8.2	7/1.75	6/28.5	9/1.9
	G242S	2/1.7	0/0.0	1/1.0	1/0.3	0/0.0	2/0.4
	D245G	1/0.8	0/0.0	1/1.0	0/0.0	1/4.8	0/0.0
	N347S	2/1.7	0/0.0	2/2.1	0/0.0	0/0.0	2/0.4
	V356I	9/7.6	35/9.2	8/8.2	36/9.0	2/9.5	42/8.8
	N373I	2/1.7	0/0.0	0/0.0	2/0.5	0/0.0	2/0.4
AmpD	G148A	4/3.4	6/1.6	3/3.1	7/1.8	1/4.2	9/1.9
	absent	98/83.1	301/79.4	78/79.6	321/80.3	20/95.2	399/83.8
MexZ	G50D	1/0.8	0/0.0	n.a.*	n.a.	n.a.	n.a.
	Y52N	2/1.7	0/0.0	n.a.	n.a.	n.a.	n.a.
	A144V	1/0.8	0/0.0	n.a.	n.a.	n.a.	n.a.
	G195E	1/0.8	1/0.3	n.a.	n.a.	n.a.	n.a.
	alternative start**	14/11.9	16/4.2	n.a.	n.a.	n.a.	n.a.
	absent	24/20.3	30/7.9	n.a.	n.a.	n.a.	n.a.
NalC	A145V	4/3,4	15/4.0	3/3.1	16/4.0	n.a.	n.a.
	A186T	10/8.5	31/8.2	11/11.2	30/7.5	n.a.	n.a.
NalD	T188A	1/0.8	1/0.3	n.a.	n.a.	n.a.	n.a.
NfXB	H109D	1/0.8	2/0.5	n.a.	n.a.	n.a.	n.a.
PBP3	A28T	1/0.8	0/0.0	1/1.0	0/0.0	0/0.0	1/0.2
	G63S	2/1.7	2/0.5	2/2.0	2/0.5	1/4.2	3/0.6
	R153S	11/9.3	11/2.9	9/9.2	13/3.3	2/8.3	20/4.2
	N242S	1/0.8	0/0.0	1/1.0	0/0.0	0/0.0	1/0.2
	G243S	3/2.5	2/0.5	2/2.0	3/0.8	2/8.3	3/0.6
	A244T	2/1.7	0/0.0	2/2.0	0/0.0	1/4.2	1/0.2
	N283S	1/0.8	0/0.0	1/1.0	0/0.0	0/0.0	1/0.2
	R504C	19/16.1	15/4.0	13/13.3	21/5.3	4/16.7	30/6.3
	F507L	2/1.7	1/0.3	1/1.0	2/0.5	0/0.0	3/0.6
	P527S	3/2.5	1/0.3	2/2.0	2/0.5	0/0.0	4/0.8
	F333L	3/2.5	0/0.0	2/2.0	1/0.3	2/8.3	1/0.2
PBP4	–	-/-	-/-	-/-	-/-	-/-	-/-

### Piperacillin/tazobactam

Piperacillin/tazobactam resistance may be caused by amino acid changes in AmpC, OXA-ß-lactamases, MexAB-OprM, MexCD-OprJ and their regulators, and also by changes in PBP3 and PBP4. The V239A amino acid change in AmpC and the R153S and R504C amino acid changes in PBP3 were found more often in resistant isolates. Other changes were too infrequent to show any patterns (Table 7). Absence of AmpD was not associated with resistance. Of the 14 described amino acid changes in AmpC/ AmpC regulation detected in the collection used here, only 6 were found in piperacillin/tazobactam-resistant isolates, these numbers were 1 of 3 for AmpD, 11 of 16 for PBP3, 0 of 1 for PBP4 and NfxB, 2 of 5 for NalC, and 0 of 2 for NalD (Table 7).

Most OXA-ß-lactamases types present among resistant isolates were also present among susceptible isolates. Insertions and deletions in proteins involved in piperacillin/tazobactam resistance could not be related to resistance; although some of these were only present in resistant isolates, it involved 1 or 2 isolates (S3 table).

Isolates with multiple amino acid changes compared to single changes were not more common among resistant isolates (ratio 0.42 (n = 14 vs 33) and 0.45 (n = 33 vs 74) for resistant and susceptible isolates).

### Cefepime

Resistance to cefepime is caused by the same mechanisms responsible for resistance to piperacillin/tazobactam, but in addition MexXY can play a role. A total of 27/43 described amino acid changes reported to be associated with cefepime resistance were present among the resistant isolates, which were mostly also present in susceptible isolates. V239A in AmpC was 6.5 times more often present in resistant isolates than susceptible isolates; lower ratios were observed for the other changes. The absence of AmpD did not seem to influence resistance (83.1% of the resistant and 79.4% of the susceptible isolates).

In PBP3, the R153S and R504C amino acid changes were more frequently present among resistant isolates (9.3% vs 2.9% and 16.1% vs 4.0%, respectively). Some amino acid changes were only present in resistant isolates, but this was in very low numbers (Table 7).

The number of isolates with 1 versus >1 described amino acid change was higher for resistant isolates compared to susceptible isolates: 28 vs 35 (ratio 0.80) and 31 vs 72 (ratio 0.45), respectively. These results suggest that single amino acid changes may often not suffice for cefepime resistance.

The percentages of the OXA-ß-lactamase types were similar for resistant and susceptible isolates with the exception of OXA-905 which was more often found in resistant isolates (9.3% vs 1.9%). This suggests that the presence of these ß-lactamases in general does not cause cefepime resistance in this population.

MexZ, a repressor of MexXY-OprM, was absent in 20.3% of the resistant isolates compared to 7.9% for the susceptible isolates. A MexZ protein variant with a start 7 amino acids earlier and out-of-frame until E20 was also more common among resistant isolates than susceptible isolates (9.3% vs 1.9%). None of the other insertions/deletions was clearly associated with resistance; some insertion/deletions appeared to be only present in resistant isolates, but in too low numbers to draw conclusions. Also, the percentage of isolates with insertions/deletions was similar for the various proteins.

In the present study, the aim was to reconcile the phenotypic resistance of 497 *P. aeruginosa* isolates obtained from patients with chronic respiratory infection with the results of WGS. The analysis included mutations, deletions and insertions of the core genome that have been previously reported to confer resistance, and acquired resistance mechanisms. Patients with chronic lung disease seem to acquire their colonizing *P. aeruginosa* strains mostly from the environment [88], although outbreaks have been reported to occur, in particular in persons with CF [89]. This is reflected in the genetic diversity of the isolates in this study, which included 207 different sequence types. Once the bacterium has colonized the airways, genetic adaptation to this niche occurs, including adaptation to antibiotic exposure by genetic mutations and deletions. This may explain why acquired resistance genes were only found in 17.1% of the isolates in this collection, and that only 51 isolates (10.2%) had acquired resistance genes for antibiotic classes that are clinically relevant in the treatment of *P. aeruginosa*, namely aminoglycosides and/or fluoroquinolones. Resistance to the different antipseudomonal antibiotics varied from 4.0% for colistin to 58.8% for ciprofloxacin, which may reflect the frequency with which these different antibiotics are used in this patient group.

In clinical practice, resistance in *P. aeruginosa* is mostly mediated by adaptation of strains, and not by acquired resistance genes, although outbreaks of *P. aeruginosa* strains harbouring acquired resistance mechanisms (mostly carbapenemases) are regularly reported in nosocomial settings [90]. This was also the case in the collection of isolates analyzed in the present study. Only 85 (17.1%) had acquired genes, and these were mostly directed at antibiotics that aren’t routinely used to treat *P. aeruginosa*, such as streptomycin, fosfomycin and chloramphenicol. Persistence of these genes might be caused by co-selection for other antibiotic resistances; although these were not found in the collection used here, co-selection due to an unidentified factor, or the presence of toxin-antitoxin systems on the mobile elements on which the resistances are most likely encoded. The presence of the ciprofloxacin resistance gene *crpP* could explain resistance in 50 of a total 292 resistant isolates in this collection (17.1%), but no acquired genes were detected that cause resistance to tobramycin, amikacin, cephalosporins, carbapenems, penicillins, monobactams or polymyxins. Therefore, antibiotic resistance to the drugs tested had to be explained almost exclusively by chromosomal mutations, insertions and deletions. Of note, EUCAST resistance breakpoints are generally set at the epidemiological cut-off (ECOFF) that distinguishes wild-type strains (in terms of antibiotic susceptibility) from non-wildtype strains [45]. It is the question whether true wildtype genotypes and phenotypes still exist in a setting of chronic infection with frequent antibiotic exposure.

Single amino acid changes often did not lead to resistance, or at least not to an MIC above the EUCAST susceptibility breakpoint for the tested antibiotics. Determining the contribution of each amino acid change to clinical resistance was complicated by the large number of described changes found and the often low frequency of these changes. Resistant isolates frequently had two or more amino acid changes, and were more likely to accumulate multiple of these changes than susceptible isolates. Also, proteins involved in antibiotic resistance are highly variable, which makes studying associations difficult.

The variation in protein sequences is illustrated by the fact that between 15 and 153 variants for each protein (PmrA and MexZ, respectively) were identified and the number of amino acid positions with changes ranged from 14 to 166 (PmrA and LptD, respectively) (Table 1). In particular, the diversity among OXA-ß-lactamases was large; despite more than 900 OXA-types in the database, more than half of the variants were undescribed [45]. These could either represent targeted adaptations or be the result of random mutations. Whether this variation is specific for isolates from the airways or whether it also occurs in other niches remains to be determined. On the other hand, a very low variability was found for GyrB, an enzyme that directly interacts with fluoroquinolones; this suggests that the structure of GyrB is highly constrained by its function.

The level of AmpC expression, regulated by both AmpR and AmpD, has been shown to determine resistance to many β-lactam antibiotics [82,91,92]. Linking mRNA expression of enzymes such as AmpC to phenotypic susceptibility under different conditions, could potentially allow a better understanding of their role in resistance; however, as shown in this study, multiple mechanisms may make a partial contribution and variations in mRNA expression may be limited. Other two-component systems, such as BqsR-BqsS, CprR-CprS, and ParR-ParS [68,91,92], were not analyzed in this study, as their roles appear limited, although perhaps depending on the niche in which the bacteria are grown. Finally, changes in the nucleotide sequences of the promoter regions may contribute to resistance, but this is still a largely unexplored field.

The limitations described above notwithstanding, there were a number of mutations which occurred relatively frequently in the collection used here. For mutations possibly related to β-lactam resistance, these were two amino acid mutations in AmpC (V239A and V356I) and two in PBP3 (R153S and R504C), three of which occurred more often in resistant isolates. Ciprofloxacin resistance could be linked more clearly to alterations in GyrA (in particular the mutations T82I and D87N) and to loss of or changes in MexZ.

Remarkably, the absence of some genes whose absence is associated with resistance, was demonstrated in a very significant number of isolates: AmpD, NfxB, and PmrA were not detected in 399 (80.2%), 217 (43.7%), and 124 (24.9%) of the isolates respectively. There was, however, no clear association with resistance, as susceptible isolates had lost these genes in similar percentages. This suggests that the loss of these genes may be involved in adaptation to the environment of chronic lung infection (also) in other ways than through antibiotic pressure. The lung is a very different niche from the environmental habitats where *P. aeruginosa* normally resides, including a very different group of micro-organisms with which to compete, and this will certainly drive bacterial evolution, just as antibiotic therapy does.

## Conclusion

For some micro-organisms, such as *Staphylococcus aureus* and *Mycobacterium tuberculosis*, WGS has been shown to reliably predict *in vitro* susceptibility, and there it may in some ways even be superior to phenotypic testing [44,93,94]. However, the understanding of the genetics of resistance has not yet evolved enough in *P. aeruginosa*, and extensive *in-vitro* testing of a very broad range of targeted mutations would be required to elucidate the effect of each separate genetic variation. Based on the results of this study, it can be concluded that often multiple mechanisms make a partial contribution to antibiotic resistance of *Pseudomonas aeruginosa* isolates. Much research is still needed to support accurate WGS-based predictions of *P. aeruginosa* susceptibility in isolates from chronic pulmonary infections.

## Supporting information

S1 Table Amino acid substitutions involved in antibiotic resistance described in literature. (PDF)

S2 Table ST & MIC results for the antibiotics tested per sequence. (PDF)

S3 Table Number of isolates/ST. (PDF)

S4 Table OXA-type ß-lactamases. (PDF)
