# Supplementary material for: Qualitative analysis of genomic mutations and antibiotic susceptibility testing of Pseudomonas aeruginosa isolates from chronic lung infections
Source: PLoS One. 2026 Mar 6;21(3):e0341613. doi: 10.1371/journal.pone.0341613 (PMC12965580; doi:10.1371/journal.pone.0341613)
Supplement: S1 Table — (PDF) [file pone.0341613.s001.pdf]

S1 table. Amino acid substitutions involved in antibiotic resistance described in literature.

gene/enzyme	change	resistance to/effect	remarks	ref
AmpC	H215Y	ß-lactam resistance	association	Colque et al, 2020
AmpC	V239A	ceftazidime-avibactam & ceftolozane-tazobactam/increased aztreonam resistance	-	Barnes et al, 2021/Skoglund et al, 2018/McLean et al, 2019/Berrazeg et al, 2015
AmpC	G242R/S	ceftazidime-avibactam & ceftolozane-tazobactam/increased aztreonam resistance	-	Barnes et al, 2021/Skoglund et al, 2018/McLean et al, 2019/Berrazeg et al, 2015
AmpC	D245G/N	increased MIC ceftazidime-avibactam & ceftolozane-tazobactam & cefepime; reduced MIC piperacil	-	Arca-Suárez et al, 2020/Ruedas-López et al, 2022
AmpC	E247G/K	ceftazidime-avibactam & ceftolozane-tazobactam	-/*in vitro* ; changes in >40 other genes	Barnes et al, 2021/Skoglund et al, 2018/Cabot et al, 2014/Berrazeg et al, 2015
AmpC	N347S/I	increased aztreonam resistance/increased ß-lactam resistance	-	McLean et al, 2019
AmpC	V356I	ceftozolane-tazobactam resistance	*in vitro* ; changes in >40 other genes	Cabot et al, 2014
AmpC	N373I	increased MIC ceftazidime-avibactam & ceftolozane-tazobactam	-	Fournier et al, 2021
AmpC	F147L+G248S	increased MIC ceftazidime-avibactam & ceftolozane-tazobactam & cefepime	-	Ruedas-López et al, 2022
AmpC	A105T+R114H+L	increased aztreonam resistance	*in vitro* induction	McLean et al, 2023
AmpC	A105T+R114H+C	increased aztreonam resistance	*in vitro* induction	McLean et al, 2021
AmpC	S15A,T16A,N49Y	increased aztreonam resistance	*in vitro* induction	McLean et al, 2020
AmpC	S15A,T16A,N49Y	increased aztreonam resistance	*in vitro* induction	McLean et al, 2019
AmpC	T21A,A105T,R11	increased aztreonam resistance	*in vitro* induction	McLean et al, 2022
AmpD	R11E	increased MICs ß-lactams		Langaee et al, 2000
AmpD	A136	AmpC derepression	based on structural analysis	Kos et al, 2016b; Liepinsh et al, 2003
AmpD	G148	AmpC derepression	based on structural analysis	Kos et al, 2016b; Liepinsh et al, 2003
EF-G*	M461V	tobramycin resistance	induced *in vitro*	Ramsay et al, 2021
EF-G	Y552C	amikacin resistance	via statistical analysis	Nageeb et al, 2022
EF-G	A555E	4-8-fold increase MIC amikacin, gentamicin, tobramycin	-	Bolard et al, 2018
EF-G	D588G	amikacin resistance	via statistical analysis	Nageeb et al, 2022
EF-G	T671A	4-8-fold increase MIC amikacin, gentamicin, tobramycin	-	Bolard et al, 2018
EF-G	V93A+I186	4-8-fold increase MIC amikacin, gentamicin, tobramycin	-	Bolard et al, 2018
GyrA	G67S	norfloxacin	-	Kugelberg et al, 2005
GyrA	T83I/A	fluoroquinolones	-	Piddock, 1999; Kugelberg et al, 2005; Pasca et al, 2012; Gomis-Font et al, 2022
GyrA	D87X	fluoroquinolones	-	Piddock, 1999
GyrA	A615P	fluoroquinolones	via statistical analysis	Marvig et al, 2013
GyrB	D315N	fluoroquinolones	via statistical analysis	Marvig et al, 2013
GyrB	T323S	norfloxacin	-	Kugelberg et al, 2005
GyrB	A372V/L	fluoroquinolones	induced *in vitro*	Feng et al, 2019
GyrB	I424L	fluoroquinolones	induced *in vitro*	Feng et al, 2020
GyrB	L464I	fluoroquinolones	induced *in vitro*	Feng et al, 2021
GyrB	S466F	not defined	via statistical analysis	Marvig et al, 2013; Torrens et al, 2022
GyrB	Q467R	fluoroquinolones	association	Rees et al, 2019;Kugelberg et al, 2005
GyrB	E468D	fluoroquinolones	via statistical analysis	Pasca et al, 2012; Marvig et al, 2013;Kugelberg et al, 2005
MexB	T329A	upregulation MexB 7 MexY; increased MIC ß-lactams	induced *in vitro* ; in combination with other mutations	Barceló et al, 2021
MexS	A166P	increased mexE expression	-	Richardot et al, 2016
MexS	C245G	increased mexE expression	-	Richardot et al, 2016
MexS	F253L	increased mexE expression	-	Richardot et al, 2016
MexS	V73A+L270Q	increased mexE expression	-	Richardot et al, 2016
MexZ	A38V	upregulation MexXY-OprM	-	Guénard et al, 2014
MexZ	G46S/V	upregulation MexXY-OprM	-	Guénard et al, 2014; Jahandideh et al, 600
MexZ	G50D	upregulation MexXY-OprM	-	Guénard et al, 2014
MexZ	T52C	up-regulation MexY	-	Suresh et al, 2018
MexZ	C113Y	reduced susceptibility ciprofloxacin	induced *in vitro*	Ramsay et al, 2021

MexZ	R139W	upregulation MexXY	-	Baum et al, 2009
MexZ	A144V	upregulation MexXY-OprM	-	Guénard et al, 2014
MexZ	G172D	upregulation MexXY-OprM	-	Guénard et al, 2014
MexZ	F192T	upregulation MexXY-OprM	-	Guénard et al, 2014
MexZ	G195E	upregulation MexXY-OprM	-	Guénard et al, 2014; Cabot et al, 2012
NalC	G71E	levofloxacin resistance	via statistical analysis	Nageeb et al, 2021
NalC	E153Q	gentamicin+levofloxacin resistance	via statistical analysis	Nageeb et al, 2021
NalC	A186T	levofloxacin resistance	via statistical analysis	Nageeb et al, 2021
NalC	G71E+S209R	carbapenem resistance	-	Tafti et al, 2020
NalC	G71E+A145V+S2	up-regulation MexA	association study	Horna et al, 2018
NalC	M151T	expression MexAB-OprN	association study	Colque et al, 2020
NalD	T43I	upregulation MexB & MexY; increased MIC ß-lactams	induced *in vitro*	Barceló et al, 2021
NalD	T188A	increased mexA expression	-	Quale et al, 2006/Braz et al, 2016
NfxB	H87R	increased OprJ expression	-	Poole et al, 1996
NfxB	H109Y	overexpression mexCD	association	Pasca et al, 2012
OprD	F170L	carbapenem resistance	in combination with other mutations	Shu et al, 2017
OprD	S278P	increased MIC cephalosporin/ß-lactamase inhibitors	possibly involved	Ruedas-López et al, 2022
OprD	deletion 312-GS(	imipenem resistance	-	Kos et al, 2016a
OprD	alteration 373-VI	increased susceptibility to meropenem	-	Kos et al, 2016b; Epp et al, 2001; Ocampo-Sosa et al, 2012; El Amin et al, 2005
ParC	S80L	fluoroquinolones	-	Higgins et al, 2003; Kugelberg et al, 2005; Park et al, 2020
ParC	S87L/W	ciprofloxacin	-	Pasca et al, 2012; Rehman et al, 2021; Torrens et al, 2022; Gomis-Font et al, 2022
ParC	P116R	fluoroquinolones	-	Higgins et al, 2003; Park et al, 2020
ParE	A425V	norfloxacin	-	Kugelberg et al, 2005
ParE	S457G	not defined	via statistical analysis	Torrens et al, 2022
ParE	E459G/R	not defined	via statistical analysis	Pasca et al, 2012; Torrens et al, 2022
ParE	E460G	ciprofloxacin	-	Rehman et al, 2021
ParE	A473V	ciprofloxacin	-	Pasca et al, 2012
ParE	L501F	norfloxacin	-	Kugelberg et al, 2005
PBP3	A28T	increased aztreonam resistance	via statistical analysis	McLean et al, 2019
PBP3	G63S	increased aztreonam resistance	via statistical analysis	McLean et al, 2019
PBP3	R153S	increased aztreonam resistance	via statistical analysis	McLean et al, 2019
PBP3	G242S	increased aztreonam resistance	via statistical analysis	McLean et al, 2019
PBP3	A244T	increased aztreonam resistance	via statistical analysis	McLean et al, 2019
PBP3	T267A	increased aztreonam resistance	via statistical analysis	McLean et al, 2019
PBP3	A454V	ß-lactam resistance	association	Clark et al, 2019
PBP3	E466L	increased aztreonam resistance	via statistical analysis	McLean et al, 2019
PBP3	K490M	increased aztreonam resistance	via statistical analysis	McLean et al, 2019
PBP3	R504C	ß-lactam resistance	association	Clark et al, 2019
PBP3	F507V/L	increased aztreonam resistance	via statistical analysis/association	McLean et al, 2019;Clark et al, 2019
PBP3	P527S	increased aztreonam resistance	via statistical analysis	McLean et al, 2019
PBP3	F533L	increased aztreonam resistance/meropenem resistance	via statistical analysis/association study	McLean et al, 2019; Colque et al, 2020
PBP3	V537L	increased aztreonam resistance	via statistical analysis	McLean et al, 2019
PBP4	G427D	ceftozolane-tazobactam resistance	*in vitro* ; changes in >40 other genes	Cabot et al, 2014
PhoQ	Y85F	amikacin resistance	via statistical analysis	Nageeb et al, 2021
PhoQ	V260G	colistin (hetero)resistance	-	Barow and Kwon, 2010;Lin et al, 2019
PmrA	L71A	gentamicin resistance	via statistical analysis	Nageeb et al, 2021

PmrB	D45E	colistin heteroresistance	-	Lin et al, 2019
PmrB	D47E	increased MIC polymyxin B	*in vitro*	Ben Jeddou et al, 2020
PmrB	A67T	colistin heteroresistance	-	Lee et al, 2016
PmrB	T132P	increased MIC polymyxin B	*in vitro*	Ben Jeddou et al, 2020
PmrB	G188D	colistin resistance	-	Moskovitz et al, 2012
PmrB	A247T	polymyxin B	-	Owusu-Anim and Kwon, 2012/Lee and Ko, 2014/Moskowitz et al, 2012
PmrB	A248V	colistin resistance	-	Moskovitz et al, 2012
PmrB	M292T	polymyxin B	-	Owusu-Anim and Kwon, 2012/Lee and Ko, 2014/Moskowitz et al, 2012
PmrB	T292C	increased MIC polymyxin B		Abraham and Kwon, 2009
PmrB	Y345H	colistin resistance/polymyxin B	-	Lin et al, 2019;Owusu-Anim and Kwon, 2012/Lee and Ko, 2014/Moskowitz et al, 2012
PmrB	L14P+P456S	colistin resistance	-	Moskovitz et al, 2012
PmrB	V15I+P216S	colistin resistance	-	Lin et al, 2019
PmrB	V15I+G68S	colistin heteroresistance	-	Lin et al, 2019
PmrB	A54V+A248T	colistin resistance	-	Moskovitz et al, 2012
PmrB	R57H+A248T	colistin resistance	-	Moskovitz et al, 2012
PmrB	R79H+R259H	colistin resistance	-	Moskovitz et al, 2012
PmrB	R135Q+M292I	colistin resistance	-	Moskovitz et al, 2012
PmrB	deletion D45	colistin resistance	-	Moskovitz et al, 2012
OXA-14	-	increased MIC ceftazidime-avibactam & ceftolozane-tazobactam; decreased MIC meropenem	-	Arca-Suárez et al, 2021
OXA-794	-	increased MIC ceftazidime-avibactam & ceftolozane-tazobactam; decreased MIC meropenem	-	Arca-Suárez et al, 2021
OXA-795	-	increased MIC ceftazidime-avibactam & ceftolozane-tazobactam; decreased MIC meropenem	-	Arca-Suárez et al, 2021
OXA-824	-	increased MIC ceftazidime-avibactam & ceftolozane-tazobactam; decreased MIC meropenem	-	Arca-Suárez et al, 2021

*Elongation factor G
