# Supplementary material for: Qualitative analysis of genomic mutations and antibiotic susceptibility testing of Pseudomonas aeruginosa isolates from chronic lung infections
Source: PLoS One. 2026 Mar 6;21(3):e0341613. doi: 10.1371/journal.pone.0341613 (PMC12965580; doi:10.1371/journal.pone.0341613)
Supplement: S2 Table — (PDF) [file pone.0341613.s002.pdf]

**S2 table.** Sequence Type (ST), disease, adult/pediatric, country, and minimal inhibory concentrations (MIC) in mg/L.

| sequence ID | ST | Disease | Adult / Pediatric | country | murepavadin | cefepime | ceftazidime | ceftazidime/avibactam | ceftolozane-tazobactam | piperacillin/tazobactam | meropenem | imipenem | aztreonam | ciprofloxacin | gentamicin | tobramycin | amikacin | colistin |
|---|---|---|---|---|---|---|---|---|---|---|---|---|---|---|---|---|---|---|
| PSA-BAB-IMI-102140 | 242 | CF | Adult | Spain | 0.06 | 2 | 0.5 | 0.5 | 0.25 | ≤0.25 | ≤0.06 | 0.25 | ≤0.25 | 1 | 2 | 0.25 | 4 | 1 |
| PSA-BAB-IMI-102141 | 508 | CF | Adult | Spain | 0.25 | 16 | 4 | 4 | 1 | 2 | 0.5 | 1 | 4 | 8 | 4 | 1 | 8 | 0.5 |
| PSA-BAB-IMI-102142 | 357 | BE/COPD | Adult | Spain | 0.12 | 4 | ≤0.25 | 0.5 | 0.12 | 1 | ≤0.06 | 1 | 4 | 8 | 8 | 16 | 8 | 0.5 |
| PSA-BAB-IMI-102143 | 27 | CF | Pediatric | Spain | 0.25 | 2 | 1 | 0.5 | 0.5 | 0.5 | ≤0.06 | 0.5 | 0.5 | 0.5 | 4 | 1 | 16 | 1 |
| PSA-BAB-IMI-102177 | 575 | CF | Adult | Spain | 0.25 | 4 | 0.5 | 1 | 1 | ≤0.25 | 0.5 | 8 | ≤0.25 | 0.25 | 4 | ≤0.12 | 16 | 0.5 |
| PSA-BAB-IMI-102178 | 569 | CF | Adult | Spain | 0.25 | 2 | 1 | 1 | 0.5 | 2 | ≤0.06 | 1 | 0.5 | 4 | 0.5 | ≤0.12 | 1 | 0.5 |
| PSA-BAB-IMI-102179 | 575 | CF | Adult | Spain | 0.06 | 64 | 128 | >256 | 32 | 256 | 32 | 64 | 64 | 2 | 4 | 2 | 16 | 0.5 |
| PSA-BAB-IMI-102180 | 254 | CF | Adult | Spain | 0.12 | 2 | 0.5 | 2 | 0.25 | 4 | 0.12 | 1 | 2 | 0.5 | 4 | 1 | 16 | 0.5 |
| PSA-BAB-IMI-102181 | 508 | CF | Adult | Spain | 0.06 | 4 | 2 | 2 | 0.5 | 4 | 1 | 8 | 8 | 4 | 0.25 | 2 | 2 | 0.5 |
| PSA-BAB-IMI-102182 | 27 | CF | | Spain | 0.06 | 4 | 1 | 2 | 0.5 | 8 | 0.12 | 1 | 8 | 2 | 4 | 1 | 16 | 1 |
| PSA-BAB-IMI-102183 | 27 | CF | | Spain | 0.03 | 4 | 1 | 1 | 0.5 | 0.5 | ≤0.06 | 1 | 0.5 | 0.25 | 4 | 0.5 | 8 | 0.5 |
| PSA-BAB-IMI-102184 | 508 | CF | | Spain | 0.06 | 4 | 1 | 4 | 0.5 | 2 | ≤0.06 | 1 | 0.5 | 0.5 | 1 | 0.25 | 2 | 1 |
| PSA-BAB-IMI-102185 | 360 | CF | Adult | Spain | 1 | 8 | 4 | 0.5 | 2 | 8 | 2 | 4 | 8 | 1 | 8 | 2 | 16 | 1 |
| PSA-BAB-IMI-102186 | 500 | CF | Adult | Spain | 0.25 | 8 | 4 | 4 | 1 | 4 | 0.25 | 2 | 16 | 0.5 | 8 | 2 | 16 | 1 |
| PSA-BAB-IMI-102187 | 245 | CF | Adult | Spain | 0.06 | 0.25 | ≤0.25 | ≤0.25 | 0.12 | ≤0.25 | ≤0.06 | 0.25 | 0.5 | 0.5 | 0.25 | ≤0.12 | 0.25 | 0.5 |
| PSA-BAB-IMI-102188 | 1883 | CF | Adult | Spain | 0.12 | 8 | 0.5 | 2 | 2 | 4 | ≤0.06 | 1 | 16 | 0.25 | 8 | 2 | 8 | 1 |
| PSA-BAB-IMI-102189 | 313 | CF | Adult | Spain | 1 | 64 | 64 | 8 | 8 | 8 | 16 | 8 | 32 | 1 | >128 | 16 | 128 | 2 |
| PSA-BAB-IMI-102190 | 395 | CF | Adult | Spain | 0.12 | 2 | 2 | ≤0.25 | 1 | ≤0.25 | 0.12 | 0.25 | 1 | 0.5 | 2 | 2 | >128 | 0.5 |
| PSA-BAB-IMI-102191 | 1109 | CF | Adult | Spain | 0.5 | 8 | 1 | 4 | 1 | 4 | 0.12 | 1 | 2 | 0.5 | >128 | 32 | >128 | 0.5 |
| PSA-BAB-IMI-102192 | 189 | CF | Pediatric | Spain | 0.06 | 8 | 4 | 4 | 1 | 16 | 8 | 16 | 32 | 1 | 4 | 1 | 8 | 1 |
| PSA-BAB-IMI-102193 | 508 | CF | Adult | Spain | 0.12 | 4 | 2 | 0.5 | 0.12 | 0.5 | 1 | 8 | 1 | 1 | 4 | 1 | 8 | 0.5 |
| PSA-BAB-IMI-102194 | 508 | CF | Adult | Spain | 0.12 | 16 | 64 | ≤0.25 | 4 | 16 | 16 | 2 | 128 | 8 | >128 | 64 | >128 | 1 |
| PSA-BAB-IMI-102195 | 508 | CF | Adult | Spain | 0.12 | 8 | 64 | 2 | 1 | ≤0.25 | 4 | 32 | >256 | 2 | 4 | 2 | 16 | 0.5 |
| PSA-BAB-IMI-102196 | 244 | BE/COPD | Adult | Spain | 0.12 | 0.5 | 1 | 1 | 0.25 | 2 | 0.5 | 2 | 4 | 0.25 | 1 | 0.5 | 2 | 1 |
| PSA-BAB-IMI-102197 | 179 | BE/COPD | Adult | Spain | 0.03 | 4 | 4 | 4 | 1 | 16 | 1 | 2 | 64 | 0.5 | 0.5 | 0.5 | 2 | 1 |
| PSA-BAB-IMI-102198 | 508 | CF | Adult | the Netherlands | 0.5 | 32 | >256 | 4 | 4 | >256 | 16 | 32 | 128 | 16 | 8 | 4 | 16 | 1 |
| PSA-BAB-IMI-102199 | 406 | CF | Pediatric | the Netherlands | 1 | 8 | 4 | 1 | 1 | 0.5 | 16 | 32 | 4 | 4 | 64 | 64 | 64 | 2 |
| PSA-BAB-IMI-102200 | 491 | CF | Pediatric | the Netherlands | 2 | 8 | 4 | 1 | 0.5 | 2 | 0.5 | 4 | 2 | 8 | 8 | 2 | 16 | 8 |
| PSA-BAB-IMI-102276 | 885 | CF | Pediatric | Spain | 0.06 | 16 | 2 | 2 | 1 | 4 | 0.25 | 1 | 8 | 0.5 | >128 | >128 | 8 | 1 |
| PSA-BAB-IMI-102277 | 385 | CF | Adult | Spain | 0.03 | ≤0.12 | 0.5 | 0.5 | 0.25 | 0.5 | ≤0.06 | 0.5 | 0.5 | ≤0.03 | 0.25 | 0.25 | 0.12 | 0.5 |
| PSA-BAB-IMI-102278 | 274 | CF | Adult | Spain | 0.12 | 8 | 4 | 1 | 1 | 1 | 4 | 32 | 2 | 0.5 | 2 | 0.5 | 8 | 1 |
| PSA-BAB-IMI-102279 | 116 | CF | Adult | Spain | 0.25 | 16 | 2 | 2 | 2 | 1 | 1 | 16 | 1 | 2 | 16 | 2 | 32 | 1 |
| PSA-BAB-IMI-102280 | 2409 | CF | Pediatric | Spain | 0.12 | 4 | 1 | 1 | 0.5 | 2 | ≤0.06 | 0.5 | 1 | 1 | 4 | 1 | 8 | 0.5 |
| PSA-BAB-IMI-102281 | 253 | CF | Adult | Spain | 0.25 | 4 | 1 | 1 | 1 | 1 | 0.12 | 1 | 0.5 | 1 | >128 | >128 | 64 | 1 |
| PSA-BAB-IMI-102282 | 316 | CF | Pediatric | Spain | 0.06 | 0.5 | ≤0.25 | 0.5 | 0.25 | 1 | ≤0.06 | 0.25 | 2 | 0.06 | 0.5 | ≤0.12 | 1 | 1 |
| PSA-BAB-IMI-102283 | 508 | CF | Adult | Spain | 0.12 | 4 | 16 | 1 | 1 | 4 | 0.12 | 1 | 32 | 0.5 | 8 | 4 | 32 | 1 |
| PSA-BAB-IMI-102284 | 676 | CF | Adult | Spain | 0.12 | 4 | 1 | 1 | 1 | 1 | ≤0.06 | 0.5 | 1 | 0.5 | 4 | 1 | 16 | 0.5 |
| PSA-BAB-IMI-102285 | 1883 | CF | Adult | Spain | 1 | >128 | >256 | 128 | >256 | >256 | 32 | 16 | >256 | 0.5 | 32 | 4 | 64 | 1 |
| PSA-BAB-IMI-102288 | 1912 | CF | Adult | Spain | 0.25 | 2 | 1 | 1 | 0.5 | 0.5 | 0.25 | 2 | 1 | 0.5 | 4 | 1 | 8 | 1 |
| PSA-BAB-IMI-102290 | 164 | CF | Pediatric | Spain | 0.12 | 4 | 2 | 2 | 1 | 4 | 1 | 16 | 8 | 4 | 4 | 0.5 | >128 | 0.5 |
| PSA-BAB-IMI-102291 | 253 | CF | Adult | Spain | 0.03 | 2 | ≤0.25 | ≤0.25 | 0.12 | 2 | ≤0.06 | ≤0.12 | ≤0.25 | 0.25 | 0.5 | ≤0.12 | 1 | 1 |
| PSA-BAB-IMI-102292 | 27 | CF | Pediatric | Spain | 0.03 | 8 | 8 | 8 | 2 | 16 | 4 | 8 | 128 | 4 | 2 | 0.5 | 2 | 0.5 |
| PSA-BAB-IMI-102293 | 1046 | CF | Adult | Spain | 0.12 | 16 | 8 | 1 | 2 | 32 | 0.25 | 1 | 8 | 0.5 | 64 | 16 | >128 | 1 |
| PSA-BAB-IMI-102295 | 198 | CF | Adult | Spain | 0.5 | 8 | 4 | 4 | 1 | 32 | 8 | 16 | 16 | 2 | 4 | 1 | 8 | 0.5 |
| PSA-BAB-IMI-102296 | 569 | CF | Adult | Spain | 0.12 | 2 | 1 | 1 | 1 | 4 | 0.25 | 1 | 1 | 4 | 1 | 0.25 | 2 | 1 |
| PSA-BAB-IMI-102297 | 198 | CF | Adult | Spain | 0.5 | 8 | 4 | 8 | 1 | 16 | 8 | 8 | 16 | 2 | 4 | 0.5 | 16 | 0.5 |
| PSA-BAB-IMI-102298 | 155 | CF | Adult | Spain | 0.06 | 1 | ≤0.25 | 2 | 0.5 | 2 | 1 | 0.25 | ≤0.25 | 0.25 | 0.5 | 0.5 | 1 | 1 |
| PSA-BAB-IMI-102299 | 3166 | CF | Adult | Spain | 0.03 | 0.25 | 2 | 0.5 | 1 | ≤0.25 | ≤0.06 | ≤0.12 | ≤0.25 | ≤0.03 | 1 | 8 | 4 | 1 |
| PSA-BAB-IMI-102300 | 238 | CF | Adult | Spain | 8 | >128 | 64 | 16 | 1 | >256 | 2 | 8 | >256 | 4 | >128 | >128 | 128 | >16 |
| PSA-BAB-IMI-102301 | 1884 | CF | Adult | Spain | 1 | 64 | >256 | 256 | 64 | >256 | 32 | 32 | >256 | 8 | 8 | 2 | 32 | 1 |
| PSA-BAB-IMI-102303 | 508 | CF | | Spain | 0.06 | 2 | 2 | 2 | 0.5 | 4 | 0.25 | 1 | 8 | 0.12 | 1 | 0.5 | 1 | 1 |

| | | | | | | | | | | | | | | | | | | |
|---|---|---|---|---|---|---|---|---|---|---|---|---|---|---|---|---|---|---|
| PSA-BAB-IMI-102304 | 508 | CF | | Spain | 0.12 | 16 | 1 | 32 | 1 | 1 | ≤0.06 | 1 | 1 | 0.25 | 4 | 1 | 16 | 0.5 |
| PSA-BAB-IMI-102305 | 508 | CF | | Spain | 0.12 | 4 | 2 | 2 | 1 | 1 | ≤0.06 | 1 | 1 | 0.25 | 4 | 1 | 8 | 0.5 |
| PSA-BAB-IMI-102306 | 198 | CF | Adult | Spain | ≤0.016 | 0.25 | 1 | ≤0.25 | 1 | ≤0.25 | 0.25 | 1 | 1 | 0.12 | ≤0.12 | 0.25 | 0.12 | 0.5 |
| PSA-BAB-IMI-102307 | 198 | CF | Adult | Spain | 0.5 | 2 | 1 | 1 | 0.5 | ≤0.25 | ≤0.06 | 0.5 | 0.5 | 0.5 | 64 | 8 | >128 | 1 |
| PSA-BAB-IMI-102308 | 27 | CF | | Spain | 0.03 | 8 | 2 | 2 | 1 | 8 | 1 | 16 | 16 | 1 | 4 | 1 | 16 | 0.5 |
| PSA-BAB-IMI-102309 | 27 | CF | | Spain | 0.25 | 32 | 4 | 32 | 0.5 | 32 | 2 | 16 | 16 | 1 | 4 | 0.5 | 4 | 0.5 |
| PSA-BAB-IMI-102310 | 252 | CF | | Spain | 0.03 | 2 | 4 | 2 | 0.5 | 4 | 0.12 | 1 | 16 | 0.12 | 1 | 0.5 | 2 | 0.5 |
| PSA-BAB-IMI-102312 | 702 | CF | | Spain | 0.06 | 8 | 2 | 32 | 1 | 256 | ≤0.06 | 1 | 16 | 0.5 | 8 | 0.5 | 8 | 2 |
| PSA-BAB-IMI-102313 | 605 | BE/COPD | Adult | Spain | 0.25 | 16 | 1 | 2 | 1 | 1 | ≤0.06 | 0.5 | 0.5 | 1 | 2 | 0.5 | 16 | 1 |
| PSA-BAB-IMI-102314 | 179 | BE/COPD | Adult | Spain | 0.03 | 2 | 2 | 4 | 0.5 | 8 | ≤0.06 | 0.5 | 8 | 0.06 | 2 | 0.5 | 8 | 0.5 |
| PSA-BAB-IMI-102315 | 262 | CF | Pediatric | Spain | 0.12 | 2 | 2 | 4 | 0.5 | 8 | 0.12 | 1 | 16 | 0.12 | 4 | 1 | 8 | 1 |
| PSA-BAB-IMI-102316 | 3181 | CF | Adult | Spain | 0.25 | 2 | 0.5 | 0.5 | 0.5 | ≤0.25 | 0.25 | 4 | 0.5 | 1 | 32 | 4 | 32 | 0.5 |
| PSA-BAB-IMI-102317 | 3165 | BE/COPD | Adult | Spain | 0.06 | 2 | 2 | 2 | 1 | 8 | ≤0.06 | 2 | 16 | 0.12 | 4 | 1 | 8 | 1 |
| PSA-BAB-IMI-102318 | 253 | BE/COPD | Adult | Spain | 2 | 4 | 1 | 1 | 1 | 0.5 | ≤0.06 | 1 | 4 | 1 | 8 | 2 | 16 | 1 |
| PSA-BAB-IMI-102319 | 645 | BE | Adult | the Netherlands | 0.12 | 0.25 | 1 | 1 | 0.5 | ≤0.25 | ≤0.06 | 2 | 0.5 | ≤0.03 | 0.5 | 0.25 | 2 | 1 |
| PSA-BAB-IMI-102320 | 406 | CF | Adult | the Netherlands | 0.5 | 16 | 8 | 8 | 4 | 4 | 64 | 64 | 16 | 4 | 64 | 16 | 128 | 0.5 |
| PSA-BAB-IMI-102322 | 506 | CF | Adult | the Netherlands | 0.12 | 4 | 8 | 2 | 1 | 8 | 2 | 16 | 32 | 0.5 | 8 | 1 | 16 | 1 |
| PSA-BAB-IMI-102323 | 406 | CF | Adult | the Netherlands | 0.5 | 64 | 128 | 8 | 4 | >256 | 32 | 128 | 128 | 2 | 128 | 8 | >128 | 1 |
| PSA-BAB-IMI-102325 | 1225 | CF | Adult | the Netherlands | 1 | 2 | 1 | 1 | 0.25 | 2 | ≤0.06 | 2 | 1 | 2 | 8 | 2 | 32 | 0.5 |
| PSA-BAB-IMI-102326 | 406 | CF | Adult | the Netherlands | 0.12 | 8 | 2 | 2 | 1 | 1 | 8 | 16 | 4 | 16 | 16 | 2 | 32 | 0.5 |
| PSA-BAB-IMI-102367 | 496 | CF | Adult | Spain | 0.12 | 0.5 | 4 | 2 | 1 | 2 | 1 | 2 | 16 | 4 | 2 | 2 | 8 | 1 |
| PSA-BAB-IMI-102368 | 3181 | CF | Adult | Spain | 0.5 | 4 | 1 | 1 | 0.5 | 0.5 | 0.5 | 4 | 0.5 | 1 | 32 | 4 | 64 | 1 |
| PSA-BAB-IMI-102370 | 175 | BE/COPD | Adult | Spain | 0.03 | 0.25 | ≤0.25 | 1 | 0.25 | 1 | ≤0.06 | 2 | 0.5 | 16 | >128 | 16 | 2 | 1 |
| PSA-BAB-IMI-102371 | 1329 | BE/COPD | Adult | Spain | 0.06 | 2 | 8 | 4 | 0.5 | 8 | ≤0.06 | 2 | 16 | 0.25 | 1 | 0.5 | 2 | 1 |
| PSA-BAB-IMI-102374 | 701 | BE/COPD | Adult | Spain | 0.12 | 2 | 2 | 4 | 1 | 2 | ≤0.06 | 1 | 2 | 0.06 | 2 | 1 | 8 | 0.5 |
| PSA-BAB-IMI-102447 | 235 | CF | Pediatric | Spain | 0.12 | 4 | 32 | 2 | 2 | 2 | 1 | 1 | 2 | 0.5 | >128 | 128 | 32 | 1 |
| PSA-BAB-IMI-102448 | 550 | CF | Adult | Spain | 4 | 64 | 2 | 4 | 2 | 32 | 32 | 32 | >256 | 4 | 8 | 4 | 16 | 0.5 |
| PSA-BAB-IMI-102449 | 640 | CF | Pediatric | Spain | 0.12 | 4 | 2 | 4 | 1 | 16 | 0.25 | 1 | 16 | 0.25 | 2 | 1 | 2 | 1 |
| PSA-BAB-IMI-102450 | 270 | CF | Adult | Spain | 0.03 | 0.25 | 4 | 1 | 0.5 | 2 | ≤0.06 | 0.5 | 8 | >32 | 2 | 0.5 | 4 | 0.5 |
| PSA-BAB-IMI-102451 | 2363 | CF | Adult | Spain | 0.12 | 4 | 2 | 2 | 2 | 4 | ≤0.06 | 0.25 | 4 | 4 | 4 | 32 | 8 | 1 |
| PSA-BAB-IMI-102452 | 313 | CF | Adult | Spain | 0.06 | 8 | 2 | 2 | 1 | 4 | ≤0.06 | 1 | 8 | 0.12 | 4 | 1 | 16 | 1 |
| PSA-BAB-IMI-102453 | 395 | CF | Adult | Spain | 0.03 | 4 | 0.5 | 1 | 0.25 | 4 | 0.12 | 0.5 | ≤0.25 | 2 | 0.25 | ≤0.12 | 4 | 0.5 |
| PSA-BAB-IMI-102461 | 27 | CF | | Spain | 0.12 | 2 | 0.5 | 2 | 0.25 | 4 | ≤0.06 | 0.25 | ≤0.25 | 0.5 | 4 | 1 | 16 | 0.5 |
| PSA-BAB-IMI-102462 | 27 | CF | | Spain | 0.25 | 8 | 1 | 2 | 1 | 8 | 0.12 | 0.5 | 8 | 0.25 | 16 | 2 | 32 | 0.5 |
| PSA-BAB-IMI-102463 | 252 | CF | | Spain | 0.06 | 8 | 2 | 8 | 0.5 | 4 | 0.12 | 2 | 8 | 0.25 | 2 | 0.5 | 4 | 1 |
| PSA-BAB-IMI-102465 | 1000 | BE/COPD | Adult | Spain | 1 | 0.5 | ≤0.25 | ≤0.25 | ≤0.016 | ≤0.25 | ≤0.06 | ≤0.12 | 2 | 1 | 0.5 | ≤0.12 | 0.12 | 0.5 |
| PSA-BAB-IMI-102467 | 1225 | CF | Adult | the Netherlands | 0.5 | 4 | 1 | 0.5 | 0.5 | 0.5 | ≤0.06 | 2 | 0.5 | 8 | 8 | 2 | 16 | 0.5 |
| PSA-BAB-IMI-102468 | 1225 | CF | Adult | the Netherlands | 8 | 16 | 128 | 1 | 2 | >256 | 0.25 | 2 | 128 | 8 | >128 | 32 | >128 | 0.5 |
| PSA-BAB-IMI-102471 | 1635 | BE | Adult | Australia | 0.25 | 4 | 1 | 1 | 1 | 1 | 0.5 | 2 | 4 | 1 | 8 | 2 | 16 | 1 |
| PSA-BAB-IMI-102472 | 918 | BE | Adult | Australia | ≤0.016 | 1 | 8 | ≤0.25 | 0.25 | 1 | 0.5 | 2 | 32 | 4 | 2 | 2 | 2 | 1 |
| PSA-BAB-IMI-102475 | 1662 | BE | Adult | Australia | 0.06 | 32 | >256 | 1 | 32 | >256 | 2 | 8 | >256 | 4 | 16 | 8 | 32 | 0.5 |
| PSA-BAB-IMI-102476 | 406 | CF | Adult | the Netherlands | 16 | 4 | 1 | 0.5 | 0.5 | 0.5 | 4 | 64 | 4 | 4 | 8 | 2 | 16 | 2 |
| PSA-BAB-IMI-102477 | 406 | CF | Adult | the Netherlands | 0.25 | 4 | 4 | 2 | 1 | 2 | 16 | 32 | 2 | 32 | 32 | 16 | 64 | 1 |
| PSA-BAB-IMI-102478 | 406 | CF | Adult | the Netherlands | 0.12 | 8 | 32 | 2 | 1 | 4 | 0.5 | 4 | 32 | 8 | 8 | 16 | 16 | 1 |
| PSA-BAB-IMI-102479 | 1342 | CF | Adult | the Netherlands | 0.06 | 4 | 8 | 4 | 1 | 16 | 2 | 32 | 64 | 0.5 | 8 | 1 | 8 | 1 |
| PSA-BAB-IMI-102481 | 537 | CF | Pediatric | the Netherlands | 0.12 | 16 | 4 | 4 | 2 | 16 | 1 | 2 | 8 | 4 | 1 | 0.5 | 2 | 1 |
| PSA-BAB-IMI-102482 | 348 | CF | Adult | the Netherlands | 0.5 | 8 | 2 | 2 | 1 | 1 | 0.12 | 1 | >256 | 8 | >128 | 128 | >128 | 8 |
| PSA-BAB-IMI-102483 | 1238 | CF | Adult | the Netherlands | 0.06 | 8 | 1 | 2 | 0.5 | 4 | ≤0.06 | 0.25 | 4 | 2 | 1 | 0.25 | 2 | 1 |
| PSA-BAB-IMI-102484 | 1238 | CF | Adult | the Netherlands | 0.06 | 16 | 1 | 2 | 0.5 | 8 | ≤0.06 | 0.25 | 2 | 2 | 1 | 0.25 | 2 | 0.5 |
| PSA-BAB-IMI-102485 | 267 | CF | Adult | the Netherlands | 0.03 | 2 | 4 | 1 | 1 | 2 | 2 | ≤0.12 | 2 | 8 | 1 | 2 | 4 | 1 |
| PSA-BAB-IMI-102486 | 267 | CF | Adult | the Netherlands | 0.12 | 1 | 1 | 2 | 0.25 | 2 | ≤0.06 | ≤0.12 | 8 | 8 | 0.25 | ≤0.12 | 4 | 0.5 |
| PSA-BAB-IMI-102487 | 3176 | CF | Adult | the Netherlands | 0.06 | 16 | 4 | 4 | 1 | 8 | 0.12 | 0.25 | 16 | 16 | 2 | 0.5 | 8 | 1 |
| PSA-BAB-IMI-102488 | 775 | CF | Adult | the Netherlands | 0.12 | 2 | 1 | 2 | 0.5 | 8 | 2 | 2 | 8 | 8 | 1 | 0.25 | 2 | 1 |
| PSA-BAB-IMI-102489 | 41 | CF | Adult | the Netherlands | 0.06 | 16 | 8 | 4 | 1 | 128 | 0.12 | 0.5 | 8 | 0.5 | 2 | 0.25 | 2 | 1 |
| PSA-BAB-IMI-102490 | 3177 | CF | Adult | the Netherlands | 0.03 | 16 | 2 | 2 | 0.5 | 8 | 16 | 8 | 8 | 2 | 0.25 | ≤0.12 | 0.5 | 1 |
| PSA-BAB-IMI-102491 | 549 | CF | Adult | the Netherlands | 2 | 8 | 1 | 0.5 | 0.5 | 4 | 1 | 4 | 2 | 0.5 | 8 | 1 | 16 | 0.5 |
| PSA-BAB-IMI-102492 | 1228 | CF | Adult | the Netherlands | 0.06 | 1 | 1 | 2 | 0.25 | 2 | 0.12 | 0.25 | 8 | ≤0.03 | 1 | 0.5 | 2 | 1 |

| | | | | | | | | | | | | | | | | | | |
|---|---|---|---|---|---|---|---|---|---|---|---|---|---|---|---|---|---|---|
| PSA-BAB-IMI-102493 | 1231 | CF | Adult | the Netherlands | 16 | 8 | 2 | 4 | 1 | 16 | 8 | 32 | 8 | 4 | 16 | 4 | 32 | 1 |
| PSA-BAB-IMI-102494 | 505 | CF | Adult | the Netherlands | 0.12 | 4 | ≤0.25 | 0.5 | 0.25 | 0.5 | 0.12 | 2 | ≤0.25 | 2 | 4 | 1 | 16 | 1 |
| PSA-BAB-IMI-102495 | 406 | CF | Adult | the Netherlands | 8 | 16 | 64 | 1 | 4 | 1 | 16 | 64 | 256 | 4 | 8 | 1 | 16 | 1 |
| PSA-BAB-IMI-102496 | 1231 | CF | Adult | the Netherlands | 0.25 | 8 | 32 | 2 | 0.5 | 16 | 0.25 | 0.5 | 32 | 8 | 2 | 1 | 8 | 1 |
| PSA-BAB-IMI-102497 | 406 | CF | Adult | the Netherlands | 0.12 | 8 | 2 | 2 | 2 | 16 | 32 | 64 | 4 | 2 | 4 | 1 | 16 | 1 |
| PSA-BAB-IMI-102511 | 179 | BE | Adult | Australia | 0.06 | 0.5 | 4 | 1 | 0.5 | 8 | 0.25 | 8 | 8 | 0.12 | 0.5 | 0.5 | 2 | 2 |
| PSA-BAB-IMI-102512 | 406 | CF | Adult | the Netherlands | 0.12 | 16 | 64 | 8 | 2 | 128 | 8 | 32 | 4 | 4 | 32 | 2 | 16 | 1 |
| PSA-BAB-IMI-102513 | 3006 | CF | Adult | the Netherlands | 0.12 | 2 | 0.5 | 1 | 0.5 | 2 | 0.12 | 2 | 1 | 1 | 1 | 0.25 | 2 | 1 |
| PSA-BAB-IMI-102514 | 3006 | CF | Adult | the Netherlands | 0.06 | 2 | 0.5 | 1 | 0.25 | 2 | 0.12 | 1 | 1 | 0.5 | 1 | 0.25 | 2 | 1 |
| PSA-BAB-IMI-102515 | 406 | CF | Adult | the Netherlands | 4 | 32 | 64 | 4 | 4 | 2 | 32 | 64 | 128 | 4 | 64 | 8 | 64 | 1 |
| PSA-BAB-IMI-102516 | 406 | CF | Adult | the Netherlands | 1 | 16 | 256 | ≤0.25 | 8 | ≤0.25 | 16 | 32 | 64 | 8 | 128 | 32 | >128 | 0.5 |
| PSA-BAB-IMI-102517 | 170 | CF | Adult | the Netherlands | 2 | 1 | 2 | ≤0.25 | 2 | ≤0.25 | ≤0.06 | 1 | 0.5 | 4 | 64 | 16 | 128 | 1 |
| PSA-BAB-IMI-102518 | 170 | CF | Adult | the Netherlands | 0.06 | 8 | 32 | 0.5 | 1 | 0.5 | 16 | 32 | 64 | 1 | 2 | 0.5 | 4 | 0.5 |
| PSA-BAB-IMI-102519 | 506 | CF | Adult | the Netherlands | 8 | 8 | 16 | 2 | 2 | 16 | 16 | 64 | 16 | >32 | 1 | 1 | 2 | 1 |
| PSA-BAB-IMI-102520 | 620 | CF | Adult | the Netherlands | 0.06 | 128 | 64 | 2 | 2 | 0.5 | 2 | 0.5 | 32 | 32 | 4 | 2 | 16 | 1 |
| PSA-BAB-IMI-102521 | 506 | CF | Adult | the Netherlands | 0.12 | 8 | 2 | 2 | 0.5 | 4 | ≤0.06 | 2 | 1 | 16 | 1 | 0.25 | 2 | 0.5 |
| PSA-BAB-IMI-102522 | 506 | CF | Adult | the Netherlands | 4 | 8 | 16 | 4 | 2 | 64 | 4 | 32 | 4 | >32 | 2 | 1 | 0.25 | 0.5 |
| PSA-BAB-IMI-102523 | 480 | CF | Adult | the Netherlands | 0.06 | 2 | 1 | 1 | 0.25 | 0.5 | ≤0.06 | 1 | 0.5 | 1 | >128 | 128 | >128 | 1 |
| PSA-BAB-IMI-102524 | 480 | CF | Adult | the Netherlands | 0.06 | 4 | 0.5 | 1 | 0.25 | 0.5 | ≤0.06 | 0.5 | ≤0.25 | 1 | 128 | 32 | >128 | 1 |
| PSA-BAB-IMI-102525 | 1050 | CF | Adult | the Netherlands | 0.06 | 1 | 1 | 1 | 0.5 | ≤0.25 | 0.12 | 2 | 8 | 0.25 | 2 | 0.5 | 4 | 1 |
| PSA-BAB-IMI-102526 | 406 | CF | Adult | the Netherlands | 16 | 2 | 1 | 0.5 | 0.5 | ≤0.25 | 1 | 16 | 1 | 2 | 16 | 4 | 32 | 1 |
| PSA-BAB-IMI-102527 | 2211 | CF | Pediatric | the Netherlands | 4 | 16 | 2 | 4 | 0.5 | 8 | 0.25 | 0.5 | 4 | 1 | 8 | 2 | 16 | 1 |
| PSA-BAB-IMI-102528 | 2211 | CF | Pediatric | the Netherlands | 16 | 8 | 2 | 2 | 2 | 4 | 2 | 16 | 8 | 0.5 | 8 | 2 | 16 | 1 |
| PSA-BAB-IMI-102529 | 406 | CF | Pediatric | the Netherlands | 0.25 | 16 | 4 | 4 | 1 | 4 | 8 | 32 | 8 | 2 | 32 | 8 | 64 | 1 |
| PSA-BAB-IMI-102530 | 379 | CF | Pediatric | the Netherlands | 0.12 | 8 | 2 | 4 | 1 | 8 | 0.5 | 2 | 16 | 1 | 4 | 1 | 16 | 1 |
| PSA-BAB-IMI-102531 | 319 | CF | Pediatric | the Netherlands | 0.06 | 2 | 1 | 2 | 0.5 | 32 | 0.12 | 1 | 16 | 0.12 | 4 | 1 | 4 | 4 |
| PSA-BAB-IMI-102532 | 108 | CF | Pediatric | the Netherlands | 0.25 | 8 | 2 | 4 | 1 | 2 | ≤0.06 | 1 | 1 | 0.25 | 8 | 1 | 16 | 1 |
| PSA-BAB-IMI-102533 | xx | CF | Pediatric | the Netherlands | 0.12 | 16 | 16 | 1 | 1 | 128 | 2 | 4 | 4 | 1 | 4 | 1 | 4 | 0.5 |
| PSA-BAB-IMI-102534 | xx | CF | Pediatric | the Netherlands | 0.12 | 32 | 32 | 4 | 1 | 256 | 2 | 8 | 16 | 2 | 8 | 1 | 16 | 0.5 |
| PSA-BAB-IMI-102535 | 497 | CF | Pediatric | the Netherlands | 1 | 16 | ≤0.25 | 0.5 | 2 | 2 | 8 | 8 | ≤0.25 | 1 | >128 | >128 | >128 | 8 |
| PSA-BAB-IMI-102536 | 348 | BE | Adult | the Netherlands | 0.03 | ≤0.12 | ≤0.25 | ≤0.25 | 0.12 | ≤0.25 | ≤0.06 | 1 | ≤0.25 | 0.06 | 0.25 | ≤0.12 | 0.25 | 0.5 |
| PSA-BAB-IMI-102537 | 3168 | BE/COPD | Adult | Spain | 0.06 | 1 | 2 | 4 | 0.5 | 4 | ≤0.06 | 2 | 8 | 0.25 | 1 | 0.5 | 2 | 1 |
| PSA-BAB-IMI-102538 | 1194 | BE/COPD | Adult | Spain | 0.06 | 2 | 1 | 1 | 1 | 32 | 0.12 | 1 | 1 | ≤0.03 | 1 | 0.5 | 2 | 1 |
| PSA-BAB-IMI-102539 | 1392 | BE/COPD | Adult | Spain | 0.25 | 4 | 1 | 0.5 | 0.06 | 0.5 | ≤0.06 | 2 | 0.5 | 4 | 2 | 0.5 | 4 | 0.5 |
| PSA-BAB-IMI-102540 | 2744 | BE/COPD | Adult | Spain | 0.06 | 4 | 4 | 4 | 0.5 | 16 | 0.12 | 2 | 32 | 0.5 | 2 | 0.5 | 1 | 1 |
| PSA-BAB-IMI-102541 | 1194 | BE/COPD | Adult | Spain | 0.03 | 0.5 | 1 | 1 | 0.12 | ≤0.25 | 0.5 | 32 | 1 | 4 | 1 | 1 | 2 | 1 |
| PSA-BAB-IMI-102542 | 1058 | BE/COPD | Adult | Spain | 0.06 | 2 | 2 | 4 | 0.5 | 4 | ≤0.06 | 0.5 | 16 | 0.12 | 2 | 0.5 | 4 | 1 |
| PSA-BAB-IMI-102543 | 257 | BE/COPD | Adult | Spain | 0.06 | 2 | 1 | 2 | 0.5 | 0.5 | ≤0.06 | 2 | 0.5 | 0.06 | 2 | 0.5 | 4 | 1 |
| PSA-BAB-IMI-102544 | 3158 | BE/COPD | Adult | Spain | 0.03 | 2 | 2 | 4 | 0.5 | 8 | ≤0.06 | 1 | 8 | 0.06 | 2 | 0.5 | 2 | 0.5 |
| PSA-BAB-IMI-102545 | 155 | BE/COPD | Adult | Spain | 0.06 | 0.25 | 1 | 1 | 0.25 | ≤0.25 | ≤0.06 | 1 | 0.5 | ≤0.03 | 2 | 0.5 | 4 | 1 |
| PSA-BAB-IMI-102546 | 514 | BE/COPD | Adult | Spain | 0.12 | 2 | 1 | 2 | 0.5 | 4 | 0.12 | 2 | 8 | 2 | 2 | 1 | 4 | 1 |
| PSA-BAB-IMI-102547 | 2049 | BE/COPD | Adult | Spain | 0.06 | ≤0.12 | 0.5 | 0.5 | 0.5 | 0.5 | ≤0.06 | 1 | 1 | ≤0.03 | 0.25 | 0.5 | 1 | 0.5 |
| PSA-BAB-IMI-102548 | 381 | BE/COPD | Adult | Spain | 0.12 | 16 | 4 | 8 | 0.5 | 16 | 0.25 | 2 | 16 | 2 | 0.5 | 0.25 | 1 | 0.5 |
| PSA-BAB-IMI-102549 | 645 | CF | Adult | the Netherlands | 0.03 | 2 | 1 | 2 | 0.5 | 0.5 | ≤0.06 | 1 | 8 | 0.25 | 2 | 1 | 8 | 1 |
| PSA-BAB-IMI-102550 | 497 | CF | Adult | the Netherlands | 2 | 128 | 256 | 16 | >256 | 64 | 2 | 1 | 256 | 16 | >128 | 128 | >128 | 2 |
| PSA-BAB-IMI-102551 | 27 | BE | Adult | the Netherlands | 0.06 | 32 | 16 | 64 | 4 | >256 | 0.12 | 1 | 8 | 4 | 2 | 0.25 | 8 | 0.5 |
| PSA-BAB-IMI-102552 | 386 | BE | Adult | the Netherlands | 0.06 | 2 | 2 | 4 | 0.5 | 8 | 0.12 | 1 | 8 | 0.12 | 2 | 0.5 | 4 | 2 |
| PSA-BAB-IMI-102553 | 296 | CF | Pediatric | the Netherlands | 0.25 | 16 | 8 | 8 | 1 | 32 | 4 | 8 | 128 | 0.5 | 16 | 2 | 32 | 1 |
| PSA-BAB-IMI-102554 | 406 | CF | Adult | the Netherlands | 0.12 | 64 | 256 | 16 | 8 | >256 | 32 | 64 | >256 | 8 | 8 | 2 | 16 | 1 |
| PSA-BAB-IMI-102555 | 883 | BE/COPD | Adult | Spain | 0.12 | 0.5 | 0.5 | 0.5 | 0.12 | 0.5 | 0.25 | 1 | ≤0.25 | 0.12 | 8 | 1 | 8 | 0.5 |
| PSA-BAB-IMI-102601 | 508 | CF | | Spain | 0.06 | 8 | 2 | 8 | 0.5 | 8 | 0.12 | 1 | 16 | 0.12 | 1 | 0.5 | 2 | 0.5 |
| PSA-BAB-IMI-102603 | 17 | CF | Pediatric | the Netherlands | 0.06 | 4 | 2 | 4 | 2 | 4 | 0.12 | 1 | 8 | 2 | 2 | 0.5 | 8 | 0.5 |
| PSA-BAB-IMI-102604 | 253 | CF | Pediatric | the Netherlands | 0.12 | 2 | 0.5 | 1 | 0.12 | 1 | ≤0.06 | ≤0.12 | 0.5 | 4 | 4 | 0.5 | >128 | 0.5 |
| PSA-BAB-IMI-102605 | 17 | CF | Pediatric | the Netherlands | 0.06 | 1 | 0.5 | 1 | 0.5 | 2 | 2 | 8 | 2 | 2 | 2 | 1 | 8 | 1 |
| PSA-BAB-IMI-102606 | 253 | CF | Pediatric | the Netherlands | 0.12 | 1 | 0.5 | 2 | 2 | 2 | 0.12 | 0.5 | 1 | 0.5 | >128 | 128 | >128 | 0.5 |
| PSA-BAB-IMI-102699 | 274 | BE | Adult | Australia | 0.12 | 16 | 32 | 4 | 2 | 128 | 0.5 | 1 | 32 | 2 | 8 | 2 | 16 | 2 |
| PSA-BAB-IMI-102700 | 775 | BE | Adult | Australia | 0.25 | 32 | 16 | 2 | 1 | 128 | 0.25 | 4 | 16 | 1 | 32 | 4 | 64 | 0.5 |

| | | | | | | | | | | | | | | | | | | |
|---|---|---|---|---|---|---|---|---|---|---|---|---|---|---|---|---|---|---|
| PSA-BAB-IMI-102701 | 41 | BE | Adult | Australia | 0.12 | 4 | 4 | 2 | 2 | 16 | 0.5 | 4 | 4 | 0.25 | 8 | 1 | 16 | 1 |
| PSA-BAB-IMI-102702 | 274 | BE | Adult | Australia | 0.25 | 8 | 16 | 2 | 1 | 32 | 1 | 16 | 16 | 1 | 8 | 1 | 8 | 0.5 |
| PSA-BAB-IMI-102703 | 1886 | CF | Pediatric | Spain | 0.03 | 0.5 | ≤0.25 | ≤0.25 | 0.12 | 0.5 | ≤0.06 | ≤0.12 | 0.5 | 0.25 | ≤0.12 | 4 | 0.25 | 0.5 |
| PSA-BAB-IMI-102704 | 1641 | CF | Adult | Spain | 0.06 | 16 | 4 | 4 | 0.5 | 128 | 0.5 | 1 | 4 | 0.5 | 4 | 4 | 64 | 0.5 |
| PSA-BAB-IMI-102705 | 1894 | CF | Adult | Spain | 0.06 | 4 | 2 | 1 | 1 | ≤0.25 | ≤0.06 | 1 | 1 | 1 | 8 | 4 | 32 | 0.5 |
| PSA-BAB-IMI-102706 | 908 | CF | Adult | Spain | 16 | 4 | 1 | 1 | 1 | 4 | 0.5 | 0.25 | 8 | 0.12 | 2 | 0.5 | 4 | 1 |
| PSA-BAB-IMI-102707 | 1228 | CF | Adult | Spain | 0.12 | 8 | 2 | 2 | 1 | 1 | 0.12 | 8 | 1 | 8 | 2 | 1 | 8 | 1 |
| PSA-BAB-IMI-102708 | 3159 | CF | Adult | Spain | 2 | 128 | 4 | 2 | 0.5 | 4 | 2 | 16 | >256 | 1 | 64 | 1 | >128 | 1 |
| PSA-BAB-IMI-102709 | 395 | CF | Pediatric | Spain | 0.06 | 1 | 2 | 2 | 0.25 | 4 | 0.25 | 1 | 8 | 0.06 | 0.25 | 0.25 | 1 | 1 |
| PSA-BAB-IMI-102710 | 3183 | CF | Pediatric | Spain | 0.06 | 0.5 | 0.5 | ≤0.25 | 0.25 | ≤0.25 | ≤0.06 | 1 | ≤0.25 | 0.5 | >128 | 32 | 8 | 0.5 |
| PSA-BAB-IMI-102711 | 1748 | CF | Adult | Spain | 4 | >128 | 64 | 4 | 4 | >256 | 16 | 16 | >256 | 2 | 32 | 4 | 128 | 0.5 |
| PSA-BAB-IMI-102712 | 155 | CF | Adult | Spain | >16 | ≤0.12 | 0.5 | 0.5 | 0.5 | 0.5 | ≤0.06 | 0.25 | 0.5 | 1 | 64 | 64 | 16 | 1 |
| PSA-BAB-IMI-102713 | 760 | CF | Adult | Spain | 0.12 | 8 | 1 | 0.5 | 2 | 1 | 0.12 | 1 | 1 | 2 | 4 | 1 | 4 | 0.5 |
| PSA-BAB-IMI-102714 | 395 | CF | Adult | Spain | 0.12 | 2 | 4 | 4 | 1 | 8 | 1 | 1 | 16 | 8 | 2 | 32 | 4 | >16 |
| PSA-BAB-IMI-102715 | 3178 | CF | Adult | Spain | 0.12 | 16 | 1 | 2 | 1 | 4 | 4 | 4 | 4 | 0.25 | 4 | 1 | 16 | 1 |
| PSA-BAB-IMI-102768 | 395 | CF | Adult | the Netherlands | 0.06 | 2 | 1 | 1 | 0.5 | 2 | 0.12 | 1 | 0.5 | 0.5 | 1 | 0.25 | 2 | 0.5 |
| PSA-BAB-IMI-102769 | 1811 | CF | Adult | Spain | 0.12 | 2 | ≤0.25 | 2 | 1 | 2 | ≤0.06 | ≤0.12 | 1 | 0.5 | 4 | 0.25 | 8 | 0.5 |
| PSA-BAB-IMI-102770 | 1907 | CF | Adult | Spain | 4 | 2 | 2 | 1 | 0.12 | 4 | 0.25 | 0.5 | 16 | 1 | 8 | 2 | 16 | 4 |
| PSA-BAB-IMI-102771 | 664 | CF | Adult | Spain | 0.12 | 2 | 1 | 1 | ≤0.016 | 0.5 | 2 | 4 | 1 | ≤0.03 | 4 | 1 | 8 | 0.5 |
| PSA-BAB-IMI-102772 | 132 | CF | Adult | Spain | 0.12 | 8 | 2 | 2 | 0.5 | 64 | 0.12 | 1 | 4 | 0.5 | 8 | 0.25 | 8 | 1 |
| PSA-BAB-IMI-102773 | 3160 | CF | Adult | Spain | 0.12 | 16 | 2 | 8 | 0.5 | 8 | 0.25 | 1 | 2 | 0.25 | 16 | 4 | 64 | 0.5 |
| PSA-BAB-IMI-102774 | 871 | CF | Adult | Spain | 0.12 | 16 | 2 | 2 | 1 | 4 | 8 | 16 | 1 | 1 | 4 | 0.5 | 16 | 0.5 |
| PSA-BAB-IMI-102775 | 564 | CF | Adult | Spain | 0.25 | 4 | 1 | 1 | 0.5 | 0.5 | ≤0.06 | 0.5 | 0.5 | 0.5 | 8 | 2 | 16 | 0.5 |
| PSA-BAB-IMI-102777 | 274 | CF | Pediatric | Spain | 0.12 | 4 | 2 | 1 | 0.5 | 1 | 0.5 | 32 | 0.5 | 1 | 4 | 2 | 16 | 1 |
| PSA-BAB-IMI-102778 | 1876 | CF | Pediatric | Spain | 0.12 | 2 | 2 | 4 | 0.5 | 4 | 0.12 | 4 | 16 | 0.12 | 2 | 0.5 | 4 | 1 |
| PSA-BAB-IMI-102779 | 1874 | CF | Adult | Spain | 0.06 | 2 | 0.5 | ≤0.25 | 0.25 | ≤0.25 | ≤0.06 | 0.5 | 0.5 | 4 | 0.25 | ≤0.12 | 0.5 | 1 |
| PSA-BAB-IMI-102780 | 1874 | CF | Adult | Spain | 0.06 | 2 | 0.5 | 1 | 0.25 | 1 | ≤0.06 | 0.5 | 0.5 | 4 | 0.5 | ≤0.12 | 0.5 | 1 |
| PSA-BAB-IMI-102781 | 1874 | CF | Adult | Spain | 0.12 | 2 | 0.5 | 0.5 | 0.25 | 0.5 | ≤0.06 | 0.5 | 0.5 | 2 | 0.25 | ≤0.12 | 0.5 | 1 |
| PSA-BAB-IMI-102782 | 1884 | CF | Adult | Spain | 1 | 64 | >256 | 32 | 32 | >256 | 64 | 16 | >256 | ≤0.03 | 2 | 4 | 16 | 1 |
| PSA-BAB-IMI-102783 | 499 | CF | Adult | Spain | 0.25 | 4 | 2 | 1 | 1 | 2 | 0.25 | 1 | 1 | 8 | 4 | 2 | 8 | 0.5 |
| PSA-BAB-IMI-102784 | 1068 | CF | Adult | Spain | 0.12 | 4 | 2 | 2 | 1 | 8 | 0.12 | 0.5 | 16 | 8 | 8 | 2 | 16 | 1 |
| PSA-BAB-IMI-102785 | 1180 | CF | Adult | Spain | 0.5 | 64 | 64 | 8 | 1 | >256 | 8 | 32 | 64 | 8 | 64 | 16 | 128 | 1 |
| PSA-BAB-IMI-102786 | 560 | CF | Adult | Spain | 0.06 | 8 | 4 | 4 | 2 | 8 | 8 | 64 | 16 | 4 | 1 | 0.5 | 4 | 1 |
| PSA-BAB-IMI-102787 | 379 | CF | Pediatric | Spain | 0.06 | 2 | 1 | 2 | 0.5 | 8 | 0.25 | 0.5 | 2 | 0.06 | 1 | 0.5 | 2 | 1 |
| PSA-BAB-IMI-102788 | 617 | CF | Pediatric | Spain | 0.12 | 4 | 4 | 2 | 0.5 | 8 | 16 | 32 | 32 | 0.5 | 0.5 | 0.25 | 2 | 1 |
| PSA-BAB-IMI-102789 | 865 | CF | Adult | Spain | 0.5 | 0.5 | 0.5 | 1 | 0.5 | 1 | 0.5 | 2 | 1 | 0.25 | 0.5 | ≤0.12 | 2 | 0.5 |
| PSA-BAB-IMI-102790 | 296 | CF | Adult | Spain | 0.06 | 0.5 | 1 | 2 | 1 | 4 | 0.12 | 4 | 4 | 1 | 0.5 | 0.25 | 1 | 0.5 |
| PSA-BAB-IMI-102791 | 1872 | CF | Adult | Spain | ≤0.016 | 8 | 4 | 8 | 1 | 32 | 32 | 32 | 64 | >32 | 4 | 2 | 8 | 0.5 |
| PSA-BAB-IMI-102792 | 575 | CF | Adult | Spain | 0.06 | 32 | 256 | 4 | 16 | >256 | 16 | 128 | >256 | 2 | 8 | 2 | 16 | 2 |
| PSA-BAB-IMI-102793 | 175 | CF | Adult | Spain | 0.06 | 32 | >256 | 16 | >256 | >256 | >64 | >128 | >256 | 16 | 128 | 128 | 16 | 0.5 |
| PSA-BAB-IMI-102795 | 406 | CF | Pediatric | the Netherlands | >16 | 4 | 1 | 0.5 | 0.25 | 0.5 | 0.5 | 1 | 2 | 1 | 4 | 1 | 8 | 1 |
| PSA-BAB-IMI-102796 | 480 | CF | Pediatric | the Netherlands | 0.12 | 8 | 0.5 | 1 | 0.12 | 1 | ≤0.06 | 0.25 | 0.5 | 0.06 | 4 | 0.5 | 8 | 1 |
| PSA-BAB-IMI-102797 | 487 | CF | Pediatric | the Netherlands | 4 | 2 | ≤0.25 | 2 | 0.12 | 2 | ≤0.06 | 0.25 | ≤0.25 | 1 | 2 | 0.25 | 4 | 4 |
| PSA-BAB-IMI-102798 | 274 | CF | Pediatric | the Netherlands | 0.06 | 2 | 2 | 4 | 0.5 | 4 | 0.25 | 1 | 16 | 0.06 | 4 | 1 | 16 | 1 |
| PSA-BAB-IMI-102799 | 1312 | CF | Pediatric | the Netherlands | 0.03 | 16 | 2 | 8 | 0.5 | 64 | 16 | 32 | 16 | 0.5 | 64 | 8 | 64 | 0.5 |
| PSA-BAB-IMI-102800 | 299 | CF | Pediatric | the Netherlands | 0.12 | 4 | 32 | 4 | 1 | 64 | 0.25 | 0.5 | 16 | 0.12 | 4 | 2 | 8 | 0.5 |
| PSA-BAB-IMI-102801 | 385 | CF | Adult | Spain | 0.06 | 32 | 128 | 8 | 8 | 128 | 16 | 64 | 64 | 2 | 8 | 4 | 64 | 1 |
| PSA-BAB-IMI-102802 | 708 | CF | Adult | Spain | >16 | >128 | >256 | 32 | >256 | 256 | 32 | 32 | >256 | 0.5 | >128 | 128 | >128 | 1 |
| PSA-BAB-IMI-102803 | 1251 | CF | Adult | Spain | ≤0.016 | 4 | 2 | 4 | 0.5 | 8 | 1 | 4 | 8 | 16 | 0.5 | 0.5 | 4 | 0.5 |
| PSA-BAB-IMI-102804 | 1908 | CF | Adult | Spain | 0.5 | 4 | 4 | 1 | 0.25 | 0.5 | 2 | 8 | 0.5 | 4 | 16 | 2 | 64 | 0.5 |
| PSA-BAB-IMI-102805 | 1909 | CF | Adult | Spain | 0.5 | 4 | 2 | ≤0.25 | 1 | ≤0.25 | 0.25 | 0.5 | 16 | 0.06 | 16 | 0.5 | 32 | 1 |
| PSA-BAB-IMI-102859 | 299 | CF | Pediatric | the Netherlands | 0.25 | 4 | 8 | 4 | 1 | 64 | 0.5 | 2 | 16 | 0.12 | 8 | 2 | 16 | 1 |
| PSA-BAB-IMI-102861 | 175 | CF | Adult | the Netherlands | 2 | 8 | 1 | 2 | 1 | 1 | ≤0.06 | 0.5 | 1 | 0.25 | 8 | 2 | 16 | 0.5 |
| PSA-BAB-IMI-102862 | 170 | CF | Adult | the Netherlands | 0.12 | 32 | 2 | 4 | 2 | 256 | 0.25 | 2 | 32 | 4 | 32 | 0.5 | 32 | 0.5 |
| PSA-BAB-IMI-102863 | 155 | CF | Adult | the Netherlands | 0.03 | 1 | 2 | 2 | 0.5 | 4 | 4 | 32 | 8 | 0.12 | 0.5 | 0.5 | 2 | 1 |
| PSA-BAB-IMI-102864 | 381 | CF | Adult | the Netherlands | 0.06 | 2 | 2 | 4 | 0.5 | 8 | 0.25 | 1 | 16 | 0.12 | 1 | 0.5 | 2 | 4 |
| PSA-BAB-IMI-102865 | 1230 | CF | Adult | the Netherlands | 2 | 8 | 4 | 2 | 1 | 1 | 0.12 | 0.25 | 2 | 0.12 | 16 | 1 | 32 | 0.5 |

| | | | | | | | | | | | | | | | | | | |
|---|---|---|---|---|---|---|---|---|---|---|---|---|---|---|---|---|---|---|
| PSA-BAB-IMI-102866 | 406 | CF | Adult | the Netherlands | 0.5 | 4 | 1 | 1 | 1 | 0.5 | 1 | 16 | 0.5 | 4 | 16 | 2 | 32 | 0.5 |
| PSA-BAB-IMI-102867 | 27 | CF | Adult | the Netherlands | >16 | 0.5 | ≤0.25 | 8 | 0.5 | ≤0.25 | ≤0.06 | 0.25 | ≤0.25 | 0.5 | 4 | 1 | 16 | 1 |
| PSA-BAB-IMI-102868 | 871 | CF | Pediatric | the Netherlands | 0.06 | 4 | 2 | 1 | 1 | 8 | 0.25 | 1 | 8 | 0.12 | 4 | 0.5 | 8 | 1 |
| PSA-BAB-IMI-102869 | 108 | CF | Pediatric | the Netherlands | 1 | 8 | 0.5 | 1 | 1 | 2 | ≤0.06 | 0.25 | 0.5 | 0.5 | 2 | 0.5 | 4 | 1 |
| PSA-BAB-IMI-102870 | 260 | CF | Pediatric | the Netherlands | 8 | ≤0.12 | ≤0.25 | ≤0.25 | 0.5 | ≤0.25 | ≤0.06 | ≤0.12 | 0.5 | 0.25 | 8 | 2 | 32 | 1 |
| PSA-BAB-IMI-102871 | 609 | CF | Pediatric | the Netherlands | 0.25 | 32 | 64 | 16 | 4 | >256 | 16 | 32 | 128 | 4 | 32 | 2 | 64 | 1 |
| PSA-BAB-IMI-102872 | 111 | CF | Pediatric | the Netherlands | 0.12 | 1 | 1 | 2 | 1 | 8 | 0.12 | 2 | 4 | 0.5 | 2 | 0.5 | 2 | 1 |
| PSA-BAB-IMI-102873 | 252 | CF | Pediatric | the Netherlands | 0.12 | 8 | 2 | 4 | 2 | 8 | 0.12 | 0.5 | 16 | 0.5 | 4 | 1 | 16 | 1 |
| PSA-BAB-IMI-102874 | 1744 | CF | Pediatric | the Netherlands | 0.06 | 4 | 8 | 8 | 1 | 32 | 1 | 2 | 64 | 1 | 2 | 0.25 | 2 | 0.5 |
| PSA-BAB-IMI-102875 | 2495 | CF | Pediatric | the Netherlands | 0.03 | 4 | 2 | 2 | 1 | 8 | 0.12 | 2 | 16 | 0.5 | 4 | 1 | 32 | 1 |
| PSA-BAB-IMI-102876 | 667 | CF | Pediatric | the Netherlands | 0.06 | 2 | 2 | 2 | 0.5 | 8 | ≤0.06 | 2 | 8 | 0.25 | 1 | 0.5 | 2 | 1 |
| PSA-BAB-IMI-102877 | 108 | CF | Pediatric | the Netherlands | 0.25 | 8 | 4 | 2 | 2 | 8 | 1 | 8 | 8 | 1 | 16 | 8 | 64 | 1 |
| PSA-BAB-IMI-102878 | 1000 | CF | Pediatric | the Netherlands | 0.12 | 4 | 4 | 4 | 0.5 | 16 | 0.25 | 4 | 32 | 0.5 | 2 | 0.5 | 4 | 1 |
| PSA-BAB-IMI-102879 | 261 | CF | Pediatric | the Netherlands | 0.12 | 4 | 2 | 2 | 1 | 8 | 1 | 1 | 16 | 0.5 | 1 | 0.25 | 1 | 1 |
| PSA-BAB-IMI-102880 | 17 | CF | Pediatric | the Netherlands | 0.06 | 2 | 16 | 4 | 0.5 | 4 | 0.12 | 2 | 16 | 2 | 2 | 0.5 | 2 | 0.5 |
| PSA-BAB-IMI-102881 | 1226 | CF | Pediatric | the Netherlands | 0.06 | 2 | 4 | 4 | 0.5 | 8 | 0.12 | 2 | 16 | 0.12 | 2 | 1 | 4 | 1 |
| PSA-BAB-IMI-102882 | 198 | CF | Pediatric | the Netherlands | 0.12 | 8 | 1 | 2 | 1 | 1 | ≤0.06 | 1 | 0.5 | 1 | 32 | 4 | 64 | 0.5 |
| PSA-BAB-IMI-102891 | 319 | cF | Pediatric | the Netherlands | 0.25 | 2 | 0.5 | ≤0.25 | 0.5 | ≤0.25 | ≤0.06 | 0.5 | 0.5 | ≤0.03 | 4 | 0.5 | 8 | 0.5 |
| PSA-BAB-IMI-102892 | 1225 | CF | Adult | the Netherlands | 1 | 16 | 1 | 2 | 2 | 32 | 16 | 32 | 32 | 16 | 8 | 2 | 16 | 1 |
| PSA-BAB-IMI-102893 | 132 | CF | Adult | the Netherlands | 2 | 8 | 2 | 1 | 0.5 | 32 | 1 | 4 | 16 | 4 | 8 | 0.5 | 32 | 0.5 |
| PSA-BAB-IMI-102894 | 406 | CF | Adult | the Netherlands | 0.25 | 4 | 4 | ≤0.25 | 1 | 0.5 | 16 | 32 | 8 | ≤0.03 | 8 | 0.5 | 16 | 0.5 |
| PSA-BAB-IMI-102895 | 1315 | CF | Adult | the Netherlands | 0.12 | 128 | 8 | 2 | 2 | 2 | 32 | 128 | 64 | 16 | 8 | 1 | 16 | 0.5 |
| PSA-BAB-IMI-102896 | 406 | CF | Adult | the Netherlands | >16 | ≤0.12 | ≤0.25 | ≤0.25 | 0.25 | ≤0.25 | 0.25 | 2 | 0.5 | 0.5 | 16 | 0.25 | 64 | 0.5 |
| PSA-BAB-IMI-102897 | 406 | CF | Adult | the Netherlands | 0.5 | 2 | ≤0.25 | 1 | 0.5 | 0.5 | ≤0.06 | 1 | 1 | 2 | 8 | 2 | 32 | 0.5 |
| PSA-BAB-IMI-102898 | 439 | CF | Adult | the Netherlands | 2 | 16 | 64 | 32 | 0.5 | 4 | 32 | 32 | 128 | 16 | 8 | 4 | 32 | 0.5 |
| PSA-BAB-IMI-102899 | 385 | BE | Adult | Australia | 0.5 | 2 | 16 | ≤0.25 | 0.5 | 16 | ≤0.06 | 1 | 32 | 2 | 8 | 1 | 16 | 0.5 |
| PSA-BAB-IMI-102900 | 155 | CF | Adult | Australia | 0.25 | 4 | 8 | 1 | 1 | 16 | 1 | 2 | 8 | 1 | 8 | 2 | 16 | 2 |
| PSA-BAB-IMI-102901 | 155 | CF | Adult | Australia | 0.12 | 2 | 0.5 | 0.5 | 0.5 | 0.5 | ≤0.06 | 0.25 | 0.5 | 0.25 | 8 | 2 | 16 | 2 |
| PSA-BAB-IMI-102902 | 155 | CF | Adult | Australia | 0.12 | 8 | 8 | 4 | 1 | 4 | 1 | 4 | 32 | 2 | 16 | 4 | 16 | 1 |
| PSA-BAB-IMI-102903 | 244 | CF | Adult | Australia | 0.06 | 0.5 | 1 | 0.5 | 0.5 | ≤0.25 | 0.25 | 2 | 0.5 | 0.06 | 1 | 0.5 | 2 | 1 |
| PSA-BAB-IMI-102904 | 845 | COPD | Adult | Australia | 0.12 | 4 | 8 | 4 | 0.5 | 32 | 1 | 2 | 64 | 1 | 4 | 0.5 | 4 | 2 |
| PSA-BAB-IMI-102905 | 821 | COPD | Adult | Australia | 0.12 | 0.5 | 1 | 1 | 0.5 | 0.5 | ≤0.06 | 2 | 1 | 0.06 | 1 | 0.5 | 2 | 1 |
| PSA-BAB-IMI-102906 | 385 | COPD | Adult | Australia | 0.06 | 1 | 1 | 1 | 0.5 | 4 | 0.12 | 1 | 8 | 0.25 | 1 | 0.5 | 2 | 2 |
| PSA-BAB-IMI-102907 | 360 | COPD | Adult | Australia | 0.12 | 8 | 32 | 2 | 1 | 16 | ≤0.06 | 2 | 32 | 0.06 | 4 | 1 | 4 | 1 |
| PSA-BAB-IMI-102908 | 253 | COPD | Adult | Australia | 0.12 | 2 | 2 | 1 | 0.5 | 4 | 0.25 | 1 | 16 | 0.12 | 1 | 0.5 | 2 | 1 |
| PSA-BAB-IMI-102911 | 885 | NON-CF | Adult | Australia | 2 | >128 | 256 | 1 | 0.5 | >256 | 32 | 32 | >256 | 1 | 64 | 8 | 64 | 0.5 |
| PSA-BAB-IMI-102927 | 1872 | CF | Adult | Spain | ≤0.016 | 8 | 8 | 8 | 1 | 32 | 32 | 16 | 128 | 32 | 4 | 2 | 8 | 0.5 |
| PSA-BAB-IMI-102929 | 3179 | CF | Pediatric | the Netherlands | 0.12 | 2 | 4 | 1 | 1 | 1 | 0.12 | 0.25 | 1 | 1 | 32 | 2 | 64 | 0.5 |
| PSA-BAB-IMI-102930 | 1135 | CF | Pediatric | the Netherlands | 2 | 2 | 1 | ≤0.25 | 1 | 2 | 0.12 | 1 | 2 | 8 | 8 | 4 | 64 | 0.5 |
| PSA-BAB-IMI-102932 | 2211 | CF | Pediatric | the Netherlands | 1 | 1 | 1 | 2 | 1 | 8 | 2 | 16 | 8 | 0.12 | 1 | 0.25 | 2 | 1 |
| PSA-BAB-IMI-102933 | 3180 | CF | Pediatric | the Netherlands | 0.25 | 2 | 0.5 | 1 | 0.5 | 2 | 0.5 | 2 | 4 | 1 | 4 | 1 | 8 | 1 |
| PSA-BAB-IMI-102939 | 406 | CF | Pediatric | the Netherlands | 0.12 | 8 | 4 | 4 | 1 | 2 | 8 | 8 | 8 | 2 | 16 | 2 | 64 | 0.5 |
| PSA-BAB-IMI-102943 | 919 | BE | Adult | Australia | 0.06 | 0.5 | 16 | 0.5 | 0.5 | 16 | 4 | 1 | 32 | 2 | ≤0.12 | ≤0.12 | 0.12 | 1 |
| PSA-BAB-IMI-102944 | 807 | BE | Adult | Australia | 0.06 | 8 | 4 | 2 | 1 | 8 | 0.12 | 2 | 16 | 0.5 | 8 | 1 | 8 | 1 |
| PSA-BAB-IMI-102946 | 919 | BE | Adult | Australia | 0.12 | 0.25 | 32 | 0.5 | 1 | 1 | 2 | 4 | 32 | 1 | ≤0.12 | ≤0.12 | 0.12 | 0.5 |
| PSA-BAB-IMI-102949 | 406 | CF | Pediatric | the Netherlands | 8 | 16 | 16 | 2 | 1 | 2 | 16 | 64 | 8 | 16 | 32 | 16 | 64 | 1 |
| PSA-BAB-IMI-102953 | 525 | CF | Adult | the Netherlands | 1 | 2 | 1 | 1 | 0.25 | 2 | 0.12 | 2 | 4 | 1 | 16 | 8 | 32 | 4 |
| PSA-BAB-IMI-102954 | 521 | CF | Adult | the Netherlands | 0.5 | 32 | 128 | 2 | 2 | >256 | 4 | 32 | 128 | 1 | 32 | 4 | 32 | 1 |
| PSA-BAB-IMI-102955 | 365 | CF | Adult | the Netherlands | 0.06 | 16 | 8 | 8 | 2 | 4 | ≤0.06 | 1 | 2 | 16 | 2 | 0.5 | 2 | 0.5 |
| PSA-BAB-IMI-102957 | 620 | CF | Adult | the Netherlands | 0.06 | >128 | 64 | 2 | 4 | 2 | 2 | 2 | 4 | 16 | 4 | 2 | 16 | 1 |
| PSA-BAB-IMI-102958 | 514 | CF | Adult | the Netherlands | 1 | 32 | 128 | 16 | 4 | >256 | 8 | 32 | >256 | 2 | 32 | 4 | 32 | 1 |
| PSA-BAB-IMI-102959 | 1225 | CF | Adult | the Netherlands | 0.5 | 8 | 4 | 4 | 1 | 16 | 0.12 | 0.25 | 32 | 16 | 8 | 2 | 16 | 1 |
| PSA-BAB-IMI-102961 | 513 | CF | Adult | the Netherlands | 8 | 16 | 4 | 2 | 1 | 32 | 2 | 8 | 8 | 8 | 32 | 4 | 32 | 4 |
| PSA-BAB-IMI-102964 | 244 | CF | Pediatric | the Netherlands | 8 | 2 | 16 | ≤0.25 | ≤0.016 | 1 | 4 | 8 | 2 | 2 | ≤0.12 | 1 | 0.12 | 1 |
| PSA-BAB-IMI-102965 | 313 | NON-CF | | Northern Ireland | 1 | 8 | 32 | 32 | 2 | 16 | 0.25 | 1 | 32 | 2 | 16 | 2 | 16 | 0.5 |
| PSA-BAB-IMI-102967 | 252 | NON-CF | | Northern Ireland | 0.06 | 4 | 4 | 2 | 0.5 | 4 | 0.12 | 1 | 16 | 0.25 | 4 | 1 | 4 | 1 |
| PSA-BAB-IMI-102968 | 395 | NON-CF | | Northern Ireland | 1 | 16 | 32 | ≤0.25 | 0.25 | 0.5 | 1 | 8 | 16 | 0.25 | 8 | 2 | 4 | 0.5 |

| | | | | | | | | | | | | | | | | | | |
|---|---|---|---|---|---|---|---|---|---|---|---|---|---|---|---|---|---|---|
| PSA-BAB-IMI-102969 | 773 | NON-CF | | Northern Ireland | 0.06 | 4 | 8 | 2 | 0.25 | 32 | 0.5 | 4 | 8 | 0.12 | 2 | 0.5 | 4 | 1 |
| PSA-BAB-IMI-102970 | 2184 | NON-CF | | Northern Ireland | 0.06 | 2 | 4 | 2 | 1 | 8 | 0.12 | 2 | 16 | 4 | 4 | 1 | 4 | 1 |
| PSA-BAB-IMI-102974 | 179 | NON-CF | | Northern Ireland | 0.12 | 4 | 2 | 2 | 1 | 2 | 1 | 4 | 4 | 8 | 8 | 2 | 16 | 1 |
| PSA-BAB-IMI-102975 | 782 | NON-CF | | Northern Ireland | 0.06 | 0.5 | 1 | 1 | 0.5 | 0.5 | ≤0.06 | 1 | 0.5 | 0.5 | 2 | 1 | 4 | 0.5 |
| PSA-BAB-IMI-102976 | 612 | NON-CF | | Northern Ireland | 0.12 | 8 | 2 | 2 | 2 | 8 | ≤0.06 | 2 | 8 | 2 | 16 | 4 | 32 | 1 |
| PSA-BAB-IMI-102977 | 27 | NON-CF | | Northern Ireland | 0.12 | 8 | 2 | 2 | 1 | 1 | ≤0.06 | 1 | 1 | 1 | 8 | 2 | 16 | 1 |
| PSA-BAB-IMI-102981 | 146 | CF | Adult | Northern Ireland | 4 | 4 | 1 | ≤0.25 | 1 | ≤0.25 | 0.12 | 0.5 | ≤0.25 | 1 | 32 | 4 | 64 | 4 |
| PSA-BAB-IMI-103010 | 179 | CF | Pediatric | the Netherlands | 0.12 | 1 | 1 | 1 | 0.5 | 4 | ≤0.06 | 2 | 8 | 0.5 | 2 | 0.5 | 4 | 16 |
| PSA-BAB-IMI-103011 | 645 | CF | Pediatric | the Netherlands | 0.06 | 4 | 2 | 2 | 0.5 | 4 | 0.5 | 2 | 16 | 0.25 | 2 | 0.5 | 4 | 4 |
| PSA-BAB-IMI-103012 | 537 | CF | Pediatric | the Netherlands | 0.06 | 4 | 1 | 1 | 0.5 | 8 | 0.25 | 0.5 | 2 | 2 | 0.25 | ≤0.12 | 0.5 | 1 |
| PSA-BAB-IMI-103013 | 395 | CF | Pediatric | the Netherlands | 0.25 | 8 | 2 | 2 | 1 | 1 | 0.5 | 16 | 1 | 0.5 | 16 | 2 | 32 | 1 |
| PSA-BAB-IMI-103014 | 499 | CF | Pediatric | the Netherlands | 0.03 | 1 | 4 | 2 | 0.5 | 4 | 0.12 | 0.5 | 32 | 0.25 | 1 | 0.5 | 2 | 1 |
| PSA-BAB-IMI-103015 | 2048 | CF | Pediatric | the Netherlands | 0.12 | 2 | 1 | 2 | 0.5 | 4 | ≤0.06 | ≤0.12 | 4 | 0.5 | 2 | 0.5 | 4 | 0.5 |
| PSA-BAB-IMI-103016 | 1240 | CF | Pediatric | the Netherlands | 0.06 | 4 | 1 | 1 | 0.25 | 0.5 | 0.12 | 1 | 0.5 | 0.12 | 4 | 1 | 16 | 0.5 |
| PSA-BAB-IMI-103017 | 491 | CF | Pediatric | the Netherlands | 0.5 | 4 | 2 | 1 | 1 | 4 | 0.5 | 4 | 2 | 4 | 2 | 1 | 4 | 2 |
| PSA-BAB-IMI-103019 | 782 | NON-CF | | Northern Ireland | 0.06 | 0.5 | 2 | 2 | 1 | 0.5 | ≤0.06 | 1 | 1 | 0.12 | 2 | 0.5 | 4 | 1 |
| PSA-BAB-IMI-103021 | 1648 | NON-CF | | Northern Ireland | 0.25 | 8 | 4 | 2 | 1 | 2 | 0.5 | 4 | 8 | >32 | 4 | 0.5 | 8 | 1 |
| PSA-BAB-IMI-103022 | 252 | NON-CF | | Northern Ireland | 0.5 | 32 | 256 | 1 | 1 | 128 | 16 | 8 | >256 | 2 | 8 | 2 | 8 | 0.5 |
| PSA-BAB-IMI-103023 | 252 | NON-CF | | Northern Ireland | 0.25 | 8 | 32 | 4 | 0.5 | 128 | 0.12 | 1 | 32 | 0.5 | 8 | 4 | 16 | 0.5 |
| PSA-BAB-IMI-103024 | 260 | NON-CF | | Northern Ireland | >16 | 16 | 16 | 2 | 0.5 | 256 | 2 | 4 | 16 | 4 | 16 | 2 | 32 | >16 |
| PSA-BAB-IMI-103025 | 2451 | NON-CF | | Northern Ireland | 0.12 | 4 | 1 | 1 | 1 | 0.5 | ≤0.06 | 1 | 0.5 | 4 | 8 | 2 | 16 | 1 |
| PSA-BAB-IMI-103026 | 395 | NON-CF | | Northern Ireland | 0.06 | 4 | 2 | 0.5 | 0.5 | 32 | ≤0.06 | 0.5 | 2 | 1 | 8 | 1 | 8 | 0.5 |
| PSA-BAB-IMI-103027 | 241 | NON-CF | | Northern Ireland | 0.25 | 4 | 2 | 2 | 0.25 | 0.5 | ≤0.06 | 1 | 1 | 0.5 | 8 | 1 | 16 | 0.5 |
| PSA-BAB-IMI-103028 | 395 | NON-CF | | Northern Ireland | 0.12 | 8 | 2 | 2 | 2 | 4 | 4 | 16 | 2 | 4 | 2 | 0.25 | 4 | 0.5 |
| PSA-BAB-IMI-103029 | 560 | NON-CF | | Northern Ireland | 0.25 | 16 | 8 | 8 | 1 | 32 | 16 | 64 | 64 | 0.5 | 8 | 2 | 16 | 1 |
| PSA-BAB-IMI-103030 | 253 | BE | Adult | Northern Ireland | 0.03 | 2 | 1 | 1 | 0.5 | 2 | ≤0.06 | 0.5 | 2 | 0.5 | 0.25 | ≤0.12 | 2 | 1 |
| PSA-BAB-IMI-103031 | 3167 | CF | Adult | Northern Ireland | 0.06 | 2 | 2 | 0.5 | 0.25 | 2 | 1 | 16 | 0.5 | 1 | 1 | 0.25 | 4 | 16 |
| PSA-BAB-IMI-103032 | 253 | BE | Adult | Northern Ireland | 0.12 | 1 | 1 | 1 | 0.5 | 1 | ≤0.06 | 0.5 | 2 | 0.5 | 1 | ≤0.12 | 1 | 2 |
| PSA-BAB-IMI-103033 | 253 | BE | Adult | Northern Ireland | 0.06 | 2 | 1 | 1 | 0.25 | 2 | 0.12 | 0.5 | 32 | 0.5 | 0.5 | ≤0.12 | 1 | 1 |
| PSA-BAB-IMI-103034 | 253 | BE | Adult | Northern Ireland | 0.03 | 1 | 1 | 1 | 0.25 | 1 | 0.12 | 0.5 | 2 | 0.5 | 0.5 | 0.25 | 0.5 | 1 |
| PSA-BAB-IMI-103035 | 549 | BE | Adult | Northern Ireland | 0.12 | 2 | 2 | 4 | 0.5 | 8 | 2 | 8 | 32 | 0.25 | 0.5 | 0.5 | 2 | 1 |
| PSA-BAB-IMI-103036 | 549 | BE | Adult | Northern Ireland | 0.06 | 4 | 2 | 2 | 0.5 | 8 | 1 | 8 | 8 | 0.25 | 1 | ≤0.12 | 4 | 1 |
| PSA-BAB-IMI-103037 | 549 | BE | Adult | Northern Ireland | 0.12 | 2 | 8 | 4 | 0.5 | 4 | 2 | 8 | 16 | 0.25 | 2 | 0.5 | 2 | 1 |
| PSA-BAB-IMI-103038 | 253 | BE | Adult | Northern Ireland | 0.03 | 1 | 1 | 1 | 0.5 | 1 | ≤0.06 | 0.5 | 2 | 0.5 | 0.5 | 0.25 | 1 | 1 |
| PSA-BAB-IMI-103039 | 549 | BE | Adult | Northern Ireland | 0.12 | 2 | 2 | 2 | 0.5 | 4 | 2 | 8 | 16 | 0.12 | 1 | 0.5 | 8 | 1 |
| PSA-BAB-IMI-103040 | 549 | BE | Adult | Northern Ireland | 0.12 | 2 | 64 | 4 | 1 | 4 | >64 | 8 | >256 | 2 | 1 | 128 | 1 | >16 |
| PSA-BAB-IMI-103041 | 27 | BE | Adult | Northern Ireland | 0.06 | 0.5 | 1 | 1 | 0.5 | ≤0.25 | 32 | 4 | 8 | 1 | 2 | 4 | 4 | 1 |
| PSA-BAB-IMI-103043 | 17 | BE | Adult | Northern Ireland | 0.03 | 4 | 2 | 2 | 0.5 | 16 | 0.12 | 0.5 | 1 | 0.12 | 1 | 0.25 | 2 | 0.5 |
| PSA-BAB-IMI-103138 | 348 | CF | Pediatric | the Netherlands | 0.06 | 1 | 1 | 1 | 0.5 | 4 | 0.5 | 4 | 8 | 0.12 | 1 | 0.5 | 2 | 1 |
| PSA-BAB-IMI-103139 | 281 | CF | Pediatric | the Netherlands | 0.06 | 16 | 1 | 2 | 0.5 | 8 | 0.25 | 1 | 1 | 2 | 0.5 | 0.25 | 1 | 0.5 |
| PSA-BAB-IMI-103140 | 2629 | CF | Pediatric | the Netherlands | 0.06 | 0.25 | ≤0.25 | ≤0.25 | 0.5 | ≤0.25 | ≤0.06 | 1 | 1 | 0.06 | 0.5 | 0.25 | 1 | 1 |
| PSA-BAB-IMI-103143 | 612 | BE | Adult | Australia | 1 | 4 | 2 | 1 | 1 | 1 | 0.12 | 1 | 1 | 8 | 4 | 1 | 8 | 0.5 |
| PSA-BAB-IMI-103144 | 406 | CF | Adult | the Netherlands | 0.25 | 8 | 16 | 2 | 1 | 0.5 | 32 | 64 | 16 | 2 | 128 | 32 | 128 | 1 |
| PSA-BAB-IMI-103145 | 497 | CF | Adult | the Netherlands | 2 | 8 | 4 | 2 | 2 | 2 | 0.25 | 1 | 2 | >32 | >128 | 64 | >128 | 16 |
| PSA-BAB-IMI-103146 | 3164 | CF | Adult | the Netherlands | 0.25 | 128 | >256 | 8 | 32 | >256 | 32 | 64 | >256 | 32 | 32 | 64 | 32 | 4 |
| PSA-BAB-IMI-103147 | 1225 | CF | Adult | the Netherlands | 0.5 | 4 | 1 | 2 | 1 | 8 | 0.12 | 0.5 | 16 | 16 | 8 | 2 | 16 | 0.5 |
| PSA-BAB-IMI-103148 | 164 | CF | Adult | the Netherlands | 0.25 | 4 | 8 | 1 | 0.5 | 1 | 2 | 16 | 8 | 8 | 4 | 1 | 8 | 1 |
| PSA-BAB-IMI-103149 | 27 | NON-CF | | Northern Ireland | 0.12 | 8 | 8 | 2 | 1 | 2 | 0.12 | 1 | 4 | 2 | 8 | 1 | 16 | 0.5 |
| PSA-BAB-IMI-103150 | 1101 | NON-CF | | Northern Ireland | 0.03 | 16 | 8 | 0.5 | 1 | 128 | 2 | 16 | 16 | 0.5 | >128 | 64 | >128 | ≤0.25 |
| PSA-BAB-IMI-103152 | 836 | NON-CF | | Northern Ireland | 0.06 | 16 | 128 | 2 | 1 | 256 | 0.25 | 8 | 256 | 2 | 4 | 1 | 4 | 2 |
| PSA-BAB-IMI-103155 | 17 | CF | Adult | Northern Ireland | 0.25 | 8 | 2 | 2 | 1 | 2 | ≤0.06 | 1 | 1 | 0.25 | 16 | 2 | 64 | 0.5 |
| PSA-BAB-IMI-103157 | 549 | CF | Adult | Northern Ireland | 0.12 | 8 | 1 | 1 | 1 | 1 | ≤0.06 | 1 | 0.5 | 2 | 4 | 1 | 8 | 0.5 |
| PSA-BAB-IMI-103158 | 146 | CF | Adult | Northern Ireland | 8 | 4 | 1 | 1 | 2 | ≤0.25 | 1 | 32 | 0.5 | 1 | 64 | 8 | 128 | 8 |
| PSA-BAB-IMI-103159 | 1203 | CF | Adult | Northern Ireland | 0.12 | 2 | 1 | 1 | 0.25 | 0.5 | ≤0.06 | 1 | 0.5 | 2 | 4 | 1 | 16 | 1 |
| PSA-BAB-IMI-103160 | 1203 | CF | Adult | Northern Ireland | 0.5 | 4 | 2 | 0.5 | 0.25 | 4 | 0.25 | 2 | 0.5 | 2 | 8 | 2 | 8 | 1 |
| PSA-BAB-IMI-103161 | 1203 | CF | Adult | Northern Ireland | 1 | 4 | 1 | 1 | 0.5 | 1 | 0.25 | 2 | 0.5 | 2 | 4 | 1 | 8 | 0.5 |
| PSA-BAB-IMI-103162 | 1203 | CF | Adult | Northern Ireland | 1 | 4 | 0.5 | 0.5 | 0.25 | 0.5 | 0.12 | 2 | 0.5 | 2 | 2 | 0.5 | 4 | 0.5 |

| | | | | | | | | | | | | | | | | | | |
|---|---|---|---|---|---|---|---|---|---|---|---|---|---|---|---|---|---|---|
| PSA-BAB-IMI-103163 | 296 | CF | Adult | Northern Ireland | 0.5 | 1 | 1 | 0.5 | 0.25 | 0.5 | ≤0.06 | 1 | 0.5 | 8 | 0.5 | 0.25 | 2 | 2 |
| PSA-BAB-IMI-103164 | 569 | CF | Adult | Northern Ireland | 0.25 | 4 | 0.5 | 0.5 | 0.5 | 2 | 0.25 | 0.5 | 1 | 16 | 4 | 0.5 | 8 | 0.5 |
| PSA-BAB-IMI-103165 | 439 | CF | Adult | Northern Ireland | 0.25 | ≤0.12 | 2 | ≤0.25 | 0.12 | ≤0.25 | 0.12 | 1 | 1 | 8 | 2 | 2 | 2 | 0.5 |
| PSA-BAB-IMI-103166 | 3161 | CF | Pediatric | Northern Ireland | 0.5 | 0.5 | 1 | ≤0.25 | 1 | ≤0.25 | 0.12 | 0.5 | 0.5 | 0.5 | 4 | 2 | 32 | 1 |
| PSA-BAB-IMI-103167 | 111 | CF | Adult | Northern Ireland | 0.06 | 1 | 256 | 2 | 0.5 | 8 | 4 | 16 | >256 | 4 | 2 | 2 | 4 | 0.5 |
| PSA-BAB-IMI-103168 | 111 | CF | Adult | Northern Ireland | 0.06 | 4 | 1 | 2 | 1 | 4 | ≤0.06 | 2 | >256 | 0.12 | 2 | 0.5 | 8 | 1 |
| PSA-BAB-IMI-103169 | 146 | CF | Adult | Northern Ireland | 1 | 4 | 2 | 2 | 2 | 0.5 | 2 | 16 | 0.5 | 2 | 64 | 8 | 64 | 2 |
| PSA-BAB-IMI-103171 | 1712 | CF | Pediatric | Northern Ireland | 8 | 2 | 2 | 0.5 | 1 | ≤0.25 | 0.5 | 16 | 2 | 8 | 32 | 4 | 16 | 2 |
| PSA-BAB-IMI-103173 | 146 | CF | Adult | Northern Ireland | >16 | 0.25 | 2 | 0.5 | 2 | 2 | 0.5 | 4 | 256 | 2 | 8 | 1 | 16 | 2 |
| PSA-BAB-IMI-103174 | 146 | CF | Adult | Northern Ireland | 1 | 32 | 2 | 2 | 2 | 2 | 2 | 8 | 1 | 1 | 16 | 2 | 32 | 0.5 |
| PSA-BAB-IMI-103175 | 146 | CF | Adult | Northern Ireland | 1 | 16 | 1 | 2 | 4 | 2 | 4 | 8 | 32 | 2 | 32 | 4 | 64 | 16 |
| PSA-BAB-IMI-103176 | 146 | CF | Adult | Northern Ireland | 2 | 16 | 8 | 4 | 2 | 8 | 8 | 16 | 4 | 2 | 16 | 2 | 16 | 0.5 |
| PSA-BAB-IMI-103177 | 146 | CF | Adult | Northern Ireland | 0.12 | 128 | 256 | 8 | 1 | >256 | 0.5 | 2 | 128 | 1 | 16 | 4 | 64 | 1 |
| PSA-BAB-IMI-103178 | 146 | CF | Adult | Northern Ireland | 0.25 | 8 | 8 | 4 | 2 | 2 | 4 | 16 | 16 | 0.5 | 16 | 4 | 32 | 1 |
| PSA-BAB-IMI-103179 | 146 | CF | Adult | Northern Ireland | 0.12 | 32 | 64 | 4 | 2 | 128 | 4 | 32 | 128 | 1 | 16 | 4 | 16 | >16 |
| PSA-BAB-IMI-103180 | 389 | CF | Adult | the Netherlands | 0.5 | 16 | 8 | 4 | 2 | 32 | 4 | 0.25 | 64 | 2 | 16 | 4 | 16 | 1 |
| PSA-BAB-IMI-103181 | 27 | CF | Adult | the Netherlands | 0.12 | 0.5 | 0.5 | 0.5 | 0.25 | ≤0.25 | ≤0.06 | ≤0.12 | 0.5 | 0.12 | 1 | 0.25 | 4 | 0.5 |
| PSA-BAB-IMI-103182 | 155 | CF | Adult | the Netherlands | 0.06 | 2 | 2 | 1 | 0.5 | 4 | 4 | 32 | 16 | 0.12 | 0.5 | ≤0.12 | 2 | 1 |
| PSA-BAB-IMI-103183 | 389 | CF | Adult | the Netherlands | 0.5 | 8 | 4 | 4 | 1 | 16 | 0.5 | 0.5 | 64 | 4 | 16 | 4 | 32 | 1 |
| PSA-BAB-IMI-103184 | 245 | CF | Adult | the Netherlands | 0.06 | 2 | 2 | 4 | 0.5 | 8 | 0.5 | 4 | 16 | 2 | 2 | 0.5 | 2 | 1 |
| PSA-BAB-IMI-103185 | 245 | CF | Adult | the Netherlands | 0.06 | 4 | 1 | 2 | 0.5 | 4 | 0.5 | 4 | 256 | 2 | 0.5 | 0.5 | 1 | 1 |
| PSA-BAB-IMI-103186 | 245 | CF | Adult | the Netherlands | 0.12 | 8 | 8 | 8 | 1 | 64 | 2 | 2 | 64 | 1 | 1 | 0.5 | 2 | 2 |
| PSA-BAB-IMI-103187 | 245 | CF | Adult | the Netherlands | 0.06 | 8 | 2 | 2 | 1 | 4 | 0.5 | 1 | 4 | 2 | 1 | 0.25 | 2 | 1 |
| PSA-BAB-IMI-103221 | 148 | CF | Adult | Northern Ireland | 2 | 8 | 32 | 1 | 2 | 2 | 4 | 32 | 8 | 2 | 128 | 64 | >128 | 1 |
| PSA-BAB-IMI-103222 | 260 | CF | Pediatric | Northern Ireland | 0.12 | 16 | 4 | 1 | 0.25 | 16 | 0.12 | 2 | 1 | 0.25 | 8 | 16 | 4 | 1 |
| PSA-BAB-IMI-103223 | 1091 | CF | Adult | Northern Ireland | 0.12 | 32 | 8 | 8 | 2 | 16 | 8 | 16 | 32 | 4 | 32 | 4 | 64 | 0.5 |
| PSA-BAB-IMI-103224 | 146 | CF | Adult | Northern Ireland | 0.25 | 8 | 16 | 2 | 1 | 2 | 4 | 8 | 16 | 2 | 4 | 0.5 | 8 | 0.5 |
| PSA-BAB-IMI-103225 | 859 | CF | Adult | Northern Ireland | 1 | 4 | ≤0.25 | ≤0.25 | 2 | ≤0.25 | ≤0.06 | 1 | ≤0.25 | 16 | 32 | 32 | 32 | 2 |
| PSA-BAB-IMI-103226 | 146 | CF | Adult | Northern Ireland | 0.25 | 8 | 2 | 8 | 2 | 64 | 4 | 16 | 1 | 2 | 32 | 4 | 64 | 0.5 |
| PSA-BAB-IMI-103227 | 146 | CF | Adult | Northern Ireland | 0.25 | 64 | 16 | 16 | 4 | 2 | 2 | 8 | 2 | 4 | 8 | 2 | 16 | 0.5 |
| PSA-BAB-IMI-103228 | 146 | CF | Adult | Northern Ireland | 0.25 | 64 | 32 | 8 | 2 | 16 | 8 | 32 | 32 | 2 | 32 | 2 | 32 | 0.5 |
| PSA-BAB-IMI-103229 | 146 | CF | Adult | Northern Ireland | 0.12 | 32 | 64 | 8 | 2 | 128 | 2 | 16 | 128 | 1 | 16 | 4 | 16 | >16 |
| PSA-BAB-IMI-103230 | 146 | CF | Adult | Northern Ireland | 1 | 32 | 16 | 2 | 2 | 2 | 4 | 8 | 1 | 1 | 32 | 8 | 32 | 1 |
| PSA-BAB-IMI-103231 | 146 | CF | Adult | Northern Ireland | 2 | 128 | 256 | 16 | 8 | >256 | 8 | 16 | >256 | 2 | 64 | 16 | 64 | 1 |
| PSA-BAB-IMI-103232 | 146 | CF | Adult | Northern Ireland | 0.12 | >128 | 256 | 4 | 4 | >256 | 0.5 | 1 | 8 | 2 | 16 | 2 | 64 | 0.5 |
| PSA-BAB-IMI-103233 | 146 | CF | Adult | Northern Ireland | 0.25 | 128 | 128 | 8 | 16 | >256 | 16 | 32 | 256 | 4 | 16 | 4 | 32 | 0.5 |
| PSA-BAB-IMI-103234 | 1712 | CF | Adult | Northern Ireland | 0.5 | 128 | >256 | >256 | 32 | >256 | 32 | 32 | >256 | 4 | >128 | 32 | >128 | 0.5 |
| PSA-BAB-IMI-103235 | 1203 | CF | Adult | Northern Ireland | >16 | 2 | ≤0.25 | 0.5 | 0.12 | ≤0.25 | ≤0.06 | 0.5 | ≤0.25 | 2 | 16 | 2 | 16 | 0.5 |
| PSA-BAB-IMI-103236 | 17 | CF | Adult | Northern Ireland | 0.5 | 8 | 4 | 4 | 2 | 2 | ≤0.06 | 1 | 2 | 0.25 | 64 | 8 | 64 | 0.5 |
| PSA-BAB-IMI-103237 | 17 | CF | Adult | Northern Ireland | 0.25 | 8 | 1 | 2 | 1 | 1 | ≤0.06 | 1 | 1 | 0.5 | 8 | 2 | 16 | 1 |
| PSA-BAB-IMI-103238 | 146 | CF | Adult | Northern Ireland | 0.5 | >128 | 256 | 16 | 2 | >256 | 16 | 32 | 256 | 2 | 32 | 2 | 64 | 0.5 |
| PSA-BAB-IMI-103305 | 155 | NON-CF | | Northern Ireland | 0.06 | 8 | 16 | 1 | 0.5 | 64 | 0.25 | 4 | 64 | 2 | 2 | 0.5 | 1 | 0.5 |
| PSA-BAB-IMI-103306 | 146 | CF | Adult | Northern Ireland | 0.25 | >128 | 128 | 32 | 16 | 256 | 8 | 16 | 1 | 4 | 16 | 2 | 32 | 0.5 |
| PSA-BAB-IMI-103307 | 3174 | CF | Adult | Northern Ireland | 1 | 4 | 256 | 4 | 2 | 64 | 16 | 8 | 256 | 8 | 32 | 32 | 64 | 8 |
| PSA-BAB-IMI-103308 | 483 | CF | Adult | Northern Ireland | 0.5 | 1 | ≤0.25 | ≤0.25 | 2 | ≤0.25 | ≤0.06 | ≤0.12 | ≤0.25 | 1 | 16 | 8 | 32 | 0.5 |
| PSA-BAB-IMI-103309 | 17 | CF | Adult | Northern Ireland | 0.12 | 8 | 1 | 2 | 1 | 1 | ≤0.06 | 0.5 | 1 | 0.5 | 4 | 0.5 | 8 | 0.5 |
| PSA-BAB-IMI-103310 | 146 | CF | Adult | Northern Ireland | 1 | 2 | 0.5 | 1 | 0.25 | 1 | 0.12 | 0.25 | ≤0.25 | 1 | 2 | 0.25 | 4 | 0.5 |
| PSA-BAB-IMI-103311 | 146 | CF | Adult | Northern Ireland | 0.12 | 2 | 32 | ≤0.25 | 1 | 0.5 | 8 | 16 | 32 | 0.5 | 16 | 4 | 32 | 1 |
| PSA-BAB-IMI-103312 | 146 | CF | Adult | Northern Ireland | 0.5 | 8 | 0.5 | ≤0.25 | 2 | 0.5 | 0.5 | 4 | 0.5 | 1 | 16 | 4 | 32 | 0.5 |
| PSA-BAB-IMI-103313 | 146 | CF | Adult | Northern Ireland | 1 | 8 | 1 | 1 | 0.5 | 0.5 | 2 | 16 | 1 | 0.5 | 8 | 2 | 32 | 4 |
| PSA-BAB-IMI-103314 | 146 | CF | Adult | Northern Ireland | 0.25 | 8 | 1 | 1 | 0.5 | 1 | 0.5 | 4 | 0.5 | 1 | 16 | 2 | 32 | 0.5 |
| PSA-BAB-IMI-103315 | 146 | CF | Adult | Northern Ireland | 0.12 | 32 | 16 | 2 | 0.5 | 32 | 0.5 | 2 | 8 | 0.5 | 8 | 2 | 16 | 1 |
| PSA-BAB-IMI-103316 | 146 | CF | Adult | Northern Ireland | 0.25 | 8 | 1 | 1 | 1 | 1 | 0.5 | 4 | 1 | 0.5 | 8 | 2 | 32 | 1 |
| PSA-BAB-IMI-103317 | 3161 | CF | Adult | Northern Ireland | 0.12 | 4 | 0.5 | 1 | 0.5 | 1 | 0.25 | 4 | 0.5 | 2 | 1 | 0.25 | 2 | 0.5 |
| PSA-BAB-IMI-103318 | 296 | CF | Adult | Northern Ireland | 0.06 | 2 | 2 | 2 | 0.5 | 2 | 0.25 | 16 | 1 | 4 | 0.5 | 0.5 | 2 | 0.5 |
| PSA-BAB-IMI-103319 | 313 | CF | Adult | Northern Ireland | 0.12 | 32 | 64 | 2 | 4 | >256 | 0.25 | 4 | >256 | 4 | 64 | 16 | 128 | 1 |
| PSA-BAB-IMI-103320 | 569 | CF | Adult | Northern Ireland | 0.25 | >128 | 128 | >256 | >256 | >256 | 32 | 32 | >256 | 16 | 32 | 8 | 64 | 0.5 |

| | | | | | | | | | | | | | | | | | | |
|---|---|---|---|---|---|---|---|---|---|---|---|---|---|---|---|---|---|---|
| PSA-BAB-IMI-103321 | 829 | BE | Adult | the Netherlands | 0.5 | 8 | 32 | 2 | 1 | 128 | 1 | 8 | 4 | 2 | 8 | 2 | 16 | 1 |
| PSA-BAB-IMI-103323 | 1225 | CF | Adult | the Netherlands | 1 | 8 | 4 | 1 | 2 | 1 | ≤0.06 | 1 | 1 | 8 | 8 | 2 | 8 | 1 |
| PSA-BAB-IMI-103324 | 1225 | CF | Adult | the Netherlands | 0.5 | 8 | 1 | 1 | 0.5 | 1 | 0.12 | 4 | 1 | 8 | 4 | 4 | 4 | 0.5 |
| PSA-BAB-IMI-103325 | 260 | CF | Adult | the Netherlands | 0.12 | 8 | 2 | 2 | 1 | 8 | 0.12 | 0.5 | 8 | 0.12 | 8 | 8 | 16 | 1 |
| PSA-BAB-IMI-103326 | 1062 | CF | Adult | the Netherlands | 2 | 8 | 2 | 2 | 0.5 | 2 | 0.5 | 1 | 1 | 1 | 16 | 2 | 64 | 0.5 |
| PSA-BAB-IMI-103327 | 27 | CF | Adult | the Netherlands | 0.03 | 1 | 1 | 1 | 0.25 | 16 | ≤0.06 | 0.5 | 16 | 0.12 | 1 | 0.25 | 4 | 1 |
| PSA-BAB-IMI-103328 | 260 | CF | Adult | the Netherlands | 0.06 | 2 | 2 | 4 | 0.5 | 4 | 0.25 | 2 | 16 | 0.25 | 2 | 0.5 | 2 | 1 |
| PSA-BAB-IMI-103329 | 569 | CF | Adult | the Netherlands | >16 | 4 | 1 | 0.5 | 0.5 | 0.5 | ≤0.06 | 0.5 | 1 | 4 | 8 | 2 | 16 | 1 |
| PSA-BAB-IMI-103330 | 569 | CF | Adult | the Netherlands | 1 | 4 | 2 | 0.5 | 2 | 2 | ≤0.06 | ≤0.12 | 2 | 0.25 | 1 | 0.5 | 2 | 0.5 |
| PSA-BAB-IMI-103331 | 549 | CF | Adult | the Netherlands | 0.5 | 32 | 4 | 16 | 2 | 16 | 4 | 2 | 16 | 0.12 | 8 | 0.5 | 8 | 1 |
| PSA-BAB-IMI-103332 | 406 | CF | Adult | the Netherlands | 0.25 | 16 | 64 | 8 | 2 | >256 | 1 | 4 | 64 | 8 | 16 | 4 | 32 | 1 |
| PSA-BAB-IMI-103333 | 3169 | CF | Adult | the Netherlands | 2 | 16 | 16 | 4 | 0.5 | 128 | 8 | 16 | 8 | 2 | 2 | 0.5 | 8 | 0.5 |
| PSA-BAB-IMI-103334 | 381 | CF | Adult | the Netherlands | 0.06 | 4 | 8 | 2 | 0.5 | 8 | ≤0.06 | 1 | 32 | 0.25 | 2 | 0.5 | 8 | 0.5 |
| PSA-BAB-IMI-103335 | 2455 | CF | Adult | the Netherlands | 0.12 | 8 | 8 | 4 | 1 | 8 | 0.25 | 2 | 16 | 2 | 4 | 1 | 8 | 1 |
| PSA-BAB-IMI-103336 | 406 | CF | Adult | the Netherlands | 0.12 | 16 | 8 | 0.5 | 2 | 4 | 8 | 32 | 2 | 2 | 8 | 2 | 32 | 0.5 |
| PSA-BAB-IMI-103337 | 1226 | CF | Adult | the Netherlands | 8 | 2 | 0.5 | 0.5 | 1 | 1 | ≤0.06 | ≤0.12 | 0.5 | 4 | 64 | 16 | 128 | 0.5 |
| PSA-BAB-IMI-103338 | 1226 | CF | Adult | the Netherlands | 8 | 4 | 0.5 | 2 | 1 | 2 | ≤0.06 | 0.5 | 0.5 | 2 | 8 | 0.5 | 16 | ≤0.25 |
| PSA-BAB-IMI-103339 | 1226 | CF | Adult | the Netherlands | 4 | 2 | 0.5 | 0.5 | 0.5 | 1 | ≤0.06 | ≤0.12 | 0.5 | 2 | 64 | 16 | 64 | 0.5 |
| PSA-BAB-IMI-103340 | 553 | CF | Adult | the Netherlands | 0.06 | 4 | 2 | 1 | 0.5 | 2 | ≤0.06 | 0.25 | 1 | 4 | 0.5 | 0.25 | 1 | 0.5 |
| PSA-BAB-IMI-103341 | 553 | CF | Adult | the Netherlands | 0.06 | 4 | 16 | 1 | 0.5 | 2 | 0.25 | 2 | 4 | 8 | 0.5 | 4 | 2 | 0.5 |
| PSA-BAB-IMI-103342 | 667 | CF | Adult | the Netherlands | 0.25 | 32 | 64 | 2 | 2 | >256 | 0.25 | 2 | 64 | 4 | 16 | 8 | 32 | ≤0.25 |
| PSA-BAB-IMI-103343 | 3170 | CF | Adult | the Netherlands | 0.12 | 8 | 8 | 16 | 1 | 64 | 2 | 1 | 128 | 0.5 | 1 | 0.5 | 4 | 2 |
| PSA-BAB-IMI-103344 | 170 | CF | Adult | the Netherlands | 0.25 | 32 | 64 | 64 | 4 | 16 | 64 | 2 | 256 | 8 | 16 | 4 | 32 | 0.5 |
| PSA-BAB-IMI-103345 | 3163 | CF | Adult | the Netherlands | 0.06 | 2 | 2 | 2 | 1 | 4 | 0.12 | 1 | 8 | 0.5 | 2 | 1 | 4 | 4 |
| PSA-BAB-IMI-103346 | 2098 | CF | Adult | the Netherlands | 0.06 | 4 | 2 | 8 | 0.25 | 8 | 0.25 | 8 | 32 | 0.25 | 2 | 0.5 | 4 | 1 |
| PSA-BAB-IMI-103347 | 2455 | CF | Adult | the Netherlands | 0.06 | 8 | 4 | 4 | 1 | 16 | 0.25 | 2 | 16 | 0.5 | 4 | 1 | 8 | 0.5 |
| PSA-BAB-IMI-103428 | 274 | CF | Adult | the Netherlands | 0.12 | 16 | 32 | 2 | 2 | 128 | 0.5 | 8 | 32 | >32 | 16 | 4 | 32 | 1 |
| PSA-BAB-IMI-103431 | 1754 | CF | Pediatric | the Netherlands | 0.25 | 1 | 1 | 1 | 1 | 1 | 0.12 | 128 | 256 | 1 | 2 | 2 | 4 | 16 |
| PSA-BAB-IMI-103432 | 260 | CF | Pediatric | the Netherlands | 0.25 | 0.25 | 4 | 1 | 1 | 0.5 | ≤0.06 | 2 | 8 | 0.12 | 1 | 0.5 | 4 | 1 |
| PSA-BAB-IMI-103433 | 1158 | CF | Pediatric | the Netherlands | 0.12 | 8 | 4 | 4 | 1 | 8 | 0.25 | 2 | 32 | 0.25 | 2 | 1 | 4 | 4 |
| PSA-BAB-IMI-103434 | 569 | CF | Pediatric | the Netherlands | 0.06 | 1 | 2 | 2 | 0.5 | 4 | ≤0.06 | 2 | 16 | 0.25 | 1 | 0.5 | 2 | 1 |
| PSA-BAB-IMI-103435 | 231 | CF | Adult | the Netherlands | 0.12 | 4 | 1 | 1 | 1 | 1 | ≤0.06 | 1 | 0.5 | 0.5 | 4 | 2 | 16 | 1 |
| PSA-BAB-IMI-103436 | 3173 | CF | Adult | the Netherlands | 0.12 | 8 | 0.5 | ≤0.25 | 0.25 | 1 | ≤0.06 | 1 | 0.5 | 8 | 128 | 64 | >128 | >16 |
| PSA-BAB-IMI-103437 | 262 | CF | Adult | the Netherlands | 0.25 | 16 | 32 | 4 | 0.5 | >256 | 4 | 64 | 64 | 2 | 8 | 2 | 8 | 1 |
| PSA-BAB-IMI-103438 | 492 | CF | Adult | the Netherlands | >16 | 8 | 2 | 1 | 2 | 1 | 8 | 64 | 2 | 8 | >128 | 64 | >128 | 1 |
| PSA-BAB-IMI-103440 | 406 | CF | Adult | the Netherlands | 1 | 16 | 8 | 2 | 2 | 2 | 16 | 64 | 4 | 8 | 32 | 4 | 32 | 0.5 |
| PSA-BAB-IMI-103441 | 3172 | CF | Adult | the Netherlands | 0.12 | 32 | 64 | 2 | 1 | 256 | 16 | 32 | 256 | 8 | 8 | 2 | 16 | 2 |
| PSA-BAB-IMI-103442 | 1227 | CF | Adult | the Netherlands | 4 | 16 | 32 | ≤0.25 | 1 | ≤0.25 | 4 | 16 | >256 | 1 | 16 | 4 | 64 | 0.5 |
| PSA-BAB-IMI-103443 | 1754 | CF | Pediatric | the Netherlands | 0.12 | 2 | 2 | 2 | 0.5 | 4 | ≤0.06 | 1 | 16 | 0.25 | 2 | 1 | 4 | 0.5 |
| PSA-BAB-IMI-103444 | yy | CF | Adult | the Netherlands | 0.12 | 64 | >256 | 4 | 2 | 4 | 8 | 32 | >256 | 8 | 128 | 64 | >128 | 1 |
| PSA-BAB-IMI-103445 | 2455 | CF | Adult | the Netherlands | 0.12 | 4 | 1 | 1 | 1 | 2 | 0.12 | 1 | 4 | 0.25 | 2 | 0.25 | 8 | 1 |
| PSA-BAB-IMI-103447 | 17 | CF | Adult | the Netherlands | 0.06 | 4 | 2 | 1 | 0.5 | 4 | 0.25 | 1 | 4 | 4 | 2 | 0.5 | 2 | 1 |
| PSA-BAB-IMI-103518 | 2465 | CF | Adult | the Netherlands | >16 | 1 | 2 | 2 | 0.5 | 4 | ≤0.06 | 2 | 32 | 0.5 | 1 | 4 | 2 | >16 |
| PSA-BAB-IMI-103519 | 550 | CF | Adult | the Netherlands | 0.12 | 8 | 0.5 | 1 | 1 | 0.5 | ≤0.06 | 0.5 | 0.5 | 0.25 | 4 | 0.5 | 16 | 1 |
| PSA-BAB-IMI-103520 | 381 | CF | Adult | the Netherlands | 0.06 | 2 | 2 | 2 | 0.5 | 8 | ≤0.06 | 4 | 16 | 0.12 | 2 | 1 | 4 | 1 |
| PSA-BAB-IMI-103521 | 406 | CF | Adult | the Netherlands | 0.25 | 8 | 2 | 2 | 1 | 2 | ≤0.06 | 0.5 | 1 | 4 | 16 | 2 | 32 | 1 |
| PSA-BAB-IMI-103522 | 550 | CF | Adult | the Netherlands | 0.12 | 16 | 2 | 2 | 4 | 2 | 0.12 | 1 | 1 | 0.25 | 8 | 0.5 | 16 | 1 |
| PSA-BAB-IMI-103523 | 497 | CF | Adult | the Netherlands | 1 | 8 | 1 | 1 | 1 | 4 | 0.12 | 2 | 1 | 2 | 1 | 0.25 | 2 | 0.5 |
| PSA-BAB-IMI-103524 | 3171 | CF | Adult | the Netherlands | 0.25 | 4 | 1 | 1 | 0.25 | 4 | ≤0.06 | 1 | 0.5 | 2 | 8 | 1 | 8 | 1 |
| PSA-BAB-IMI-103525 | 633 | CF | Adult | the Netherlands | 0.12 | 1 | 1 | 2 | 0.5 | 8 | 0.12 | 0.25 | 8 | 0.12 | 1 | ≤0.12 | 2 | 1 |
| PSA-BAB-IMI-103526 | 2685 | CF | Pediatric | Spain | 0.06 | 2 | 0.5 | 2 | 0.5 | 4 | ≤0.06 | 1 | 0.5 | 4 | 1 | 4 | 2 | 0.5 |
| PSA-BAB-IMI-103527 | 260 | CF | Model | Northern Ireland | 0.25 | 4 | 2 | 2 | 1 | 8 | 1 | 8 | 16 | 4 | 16 | 2 | 16 | 2 |
| PSA-BAB-IMI-103528 | 260 | NON-CF | Adult | the Netherlands | 0.06 | 2 | 2 | 4 | 0.5 | 4 | 0.25 | 2 | 16 | 0.25 | 2 | 0.5 | 2 | 1 |
| PSA-BAB-IMI-103529 | 612 | NON-CF | | Northern Ireland | 0.06 | 2 | 2 | 2 | 0.5 | 8 | 0.12 | 0.5 | 8 | 0.5 | 2 | 0.5 | 4 | 1 |
| PSA-BAB-IMI-103533 | 17 | CF | Adult | the Netherlands | 0.12 | 4 | 4 | 1 | 1 | 1 | ≤0.06 | 1 | 256 | 2 | 4 | 2 | 8 | >16 |
| PSA-BAB-IMI-103535 | 492 | CF | Adult | the Netherlands | 0.25 | 1 | 64 | 1 | 0.25 | 1 | 4 | 32 | 32 | 8 | 8 | 2 | 32 | 2 |
| PSA-BAB-IMI-103539 | 1754 | CF | Pediatric | the Netherlands | 0.25 | 8 | 4 | 2 | 1 | 8 | ≤0.06 | 0.25 | 1 | 1 | 2 | 0.5 | 2 | 1 |

| | | | | | | | | | | | | | | | | | | |
|---|---|---|---|---|---|---|---|---|---|---|---|---|---|---|---|---|---|---|
| PSA-JSC-IMI-101961 | 491 | CF | Pediatric | the Netherlands | 0.06 | 4 | 2 | 2 | 1 | 4 | 1 | 4 | 8 | 8 | 4 | 0.5 | 4 | 1 |
| PSA-JSC-IMI-103632 | 2465 | CF | Adult | the Netherlands | >16 | 2 | 1 | 4 | 0.5 | 8 | ≤0.06 | 1 | 4 | 0.5 | 2 | 0.5 | 16 | 1 |
| PSA-JSC-IMI-103633 | 1233 | CF | Adult | the Netherlands | 0.5 | 128 | >256 | 32 | 4 | >256 | 2 | 4 | 256 | 1 | 64 | 16 | 128 | 0.5 |
| PSA-JSC-IMI-103634 | 170 | CF | Adult | the Netherlands | 0.12 | 64 | 128 | 8 | 8 | >256 | 8 | 32 | 256 | 8 | 8 | 2 | 16 | 0.5 |
| PSA-JSC-IMI-103635 | 406 | CF | Adult | the Netherlands | 2 | 4 | 1 | 2 | 2 | 0.5 | ≤0.06 | 0.5 | 1 | 2 | 32 | 4 | 32 | 0.5 |
| PSA-JSC-IMI-103636 | 564 | CF | Adult | Spain | 0.12 | 8 | 1 | 1 | 0.5 | 1 | ≤0.06 | 0.5 | 0.5 | 0.5 | 8 | 1 | 16 | 0.5 |
| PSA-JSC-IMI-103637 | 27 | CF | Pediatric | Spain | 0.5 | 1 | ≤0.25 | 0.5 | 0.12 | 1 | ≤0.06 | ≤0.12 | ≤0.25 | 0.25 | 0.5 | 0.5 | 1 | 0.5 |
| PSA-JSC-IMI-103638 | 676 | CF | Adult | Spain | 0.12 | 4 | 0.5 | 1 | 0.06 | ≤0.25 | 0.25 | 1 | ≤0.25 | 0.25 | 2 | ≤0.12 | 8 | ≤0.25 |
| PSA-JSC-IMI-103640 | 511 | CF | Adult | the Netherlands | 8 | >128 | 128 | 32 | 4 | >256 | 16 | 64 | 16 | 16 | >128 | 64 | >128 | >16 |
| PSA-JSC-IMI-103641 | 3175 | CF | Pediatric | the Netherlands | 0.03 | 2 | 1 | 2 | 0.5 | 4 | 0.25 | 2 | 8 | 2 | 0.5 | ≤0.12 | 0.5 | 0.5 |
| PSA-TIM-IMI-101097 | 316 | CF | Adult | the Netherlands | 0.06 | 4 | 1 | 2 | 0.5 | 8 | 0.12 | 2 | 8 | 8 | 2 | 0.5 | 4 | 1 |
| PSA-TIM-IMI-101098 | 500 | CF | Adult | the Netherlands | 0.06 | 2 | 1 | 1 | 0.5 | 4 | 0.12 | 2 | 0.5 | 8 | ≤0.12 | ≤0.12 | 0.25 | 1 |
| PSA-TIM-IMI-101099 | 406 | CF | Adult | the Netherlands | 4 | 4 | 1 | 1 | 0.5 | 0.5 | 8 | 32 | 2 | 2 | 4 | 1 | 8 | 1 |
| PSA-TIM-IMI-101100 | 395 | CF | Pediatric | the Netherlands | 16 | 2 | 0.5 | 1 | 0.12 | ≤0.25 | 0.25 | 2 | 1 | 0.06 | 32 | 8 | >128 | 2 |
| PSA-TIM-IMI-101101 | 390 | CF | Pediatric | the Netherlands | 0.12 | 16 | 256 | 8 | 4 | >256 | 16 | 32 | 128 | 1 | 8 | 8 | 32 | 0.5 |
| PSA-TIM-IMI-101102 | 348 | CF | Pediatric | the Netherlands | 0.12 | 4 | 4 | 2 | 0.25 | 64 | 4 | 8 | 8 | 1 | 2 | 0.5 | 4 | 1 |
| PSA-TIM-IMI-101103 | 274 | CF | Adult | the Netherlands | 0.25 | 4 | 1 | 1 | 1 | 1 | ≤0.06 | 1 | 0.5 | 4 | 4 | 1 | 8 | 0.5 |
| PSA-TIM-IMI-101104 | 267 | CF | Pediatric | the Netherlands | 0.06 | 1 | 2 | 2 | 0.5 | 4 | 1 | 4 | 8 | 0.12 | 0.5 | 0.5 | 1 | 1 |
| PSA-TIM-IMI-101105 | 266 | CF | Adult | the Netherlands | 0.06 | 1 | 1 | 2 | 0.5 | 4 | ≤0.06 | 1 | 4 | 0.25 | 2 | 1 | 4 | 1 |
| PSA-TIM-IMI-101106 | 244 | CF | Pediatric | the Netherlands | 0.12 | 2 | 2 | 2 | 0.5 | 8 | 1 | 2 | 8 | 0.12 | 2 | 1 | 4 | 1 |
| PSA-TIM-IMI-101107 | 261 | CF | Pediatric | the Netherlands | 0.12 | 8 | 4 | 4 | 2 | 64 | 1 | 2 | 32 | 0.5 | 8 | 2 | 32 | 1 |
| PSA-TIM-IMI-101108 | 260 | CF | Adult | the Netherlands | 0.5 | 8 | 2 | 4 | 1 | 32 | 0.5 | 2 | 16 | 2 | 16 | 2 | 16 | 1 |
| PSA-TIM-IMI-101109 | 27 | CF | Adult | the Netherlands | 0.25 | 16 | 4 | 1 | 0.5 | 64 | 16 | 32 | 16 | 0.25 | 4 | 2 | 16 | 0.5 |
| PSA-TIM-IMI-101110 | 262 | CF | Adult | the Netherlands | 0.12 | 8 | 1 | 2 | 1 | 1 | 0.12 | 1 | 0.5 | 0.5 | 16 | 2 | 16 | 0.5 |
| PSA-TIM-IMI-101111 | 273 | CF | Adult | the Netherlands | 0.12 | 8 | ≤0.25 | ≤0.25 | 0.12 | 16 | 0.5 | 8 | ≤0.25 | 1 | >128 | 4 | 2 | 0.5 |
| PSA-TIM-IMI-101113 | 9 | CF | Pediatric | the Netherlands | 0.25 | 8 | 2 | 2 | 1 | 2 | 0.12 | 4 | 8 | 0.25 | 8 | 1 | 16 | 1 |
| PSA-TIM-IMI-101114 | 701 | CF | Pediatric | the Netherlands | 0.12 | 4 | 4 | 8 | 0.5 | 16 | 0.12 | 1 | 64 | 0.5 | 2 | 0.5 | 2 | 1 |
| PSA-TIM-IMI-101115 | 492 | CF | Adult | the Netherlands | 0.12 | 0.25 | 1 | 1 | 0.25 | 1 | 0.5 | 1 | 4 | 4 | 2 | 0.25 | 4 | 0.5 |
| PSA-TIM-IMI-101116 | 488 | CF | Pediatric | the Netherlands | 0.25 | 8 | 4 | 8 | 1 | 32 | 4 | 1 | 16 | 2 | 8 | 2 | 16 | 1 |
| PSA-TIM-IMI-101117 | 485 | CF | Pediatric | the Netherlands | 0.12 | 4 | 0.5 | 1 | 0.5 | 2 | ≤0.06 | 0.25 | 0.5 | 8 | 0.5 | ≤0.12 | 0.25 | 1 |
| PSA-TIM-IMI-101118 | 484 | CF | Pediatric | the Netherlands | 0.5 | 16 | 2 | 2 | 1 | 1 | 2 | 2 | 1 | 2 | 32 | 4 | 32 | 1 |
| PSA-TIM-IMI-101119 | 308 | CF | Pediatric | the Netherlands | 0.06 | 0.25 | 0.5 | 0.5 | 0.5 | 0.5 | ≤0.06 | 1 | 0.5 | ≤0.03 | 2 | 4 | 2 | 0.5 |
| PSA-TIM-IMI-101120 | 406 | CF | Pediatric | the Netherlands | 0.12 | 8 | 4 | 2 | 2 | 1 | 0.5 | 2 | 16 | 8 | 16 | 4 | 32 | 1 |
| PSA-TIM-IMI-101121 | 499 | CF | Adult | the Netherlands | 0.25 | 8 | 32 | 4 | 0.5 | 256 | 16 | 32 | 32 | 1 | 4 | 2 | 8 | 1 |
| PSA-TIM-IMI-101122 | 498 | CF | Pediatric | the Netherlands | 0.06 | 1 | 2 | 2 | 1 | 1 | ≤0.06 | 1 | 1 | 1 | 4 | 1 | 16 | 1 |
| PSA-TIM-IMI-101123 | 497 | CF | Adult | the Netherlands | 0.03 | >128 | >256 | 8 | >256 | 128 | 1 | 1 | 256 | 8 | >128 | >128 | >128 | 0.5 |
| PSA-TIM-IMI-101124 | 496 | CF | Pediatric | the Netherlands | 0.12 | 4 | 1 | 2 | 0.5 | 0.5 | ≤0.06 | 2 | 1 | 0.25 | 4 | 1 | 8 | 1 |
| PSA-TIM-IMI-101125 | 244 | CF | Pediatric | the Netherlands | 0.06 | 2 | 2 | 2 | 0.5 | 8 | 1 | 2 | 8 | 0.12 | 4 | 0.5 | 2 | 1 |
| PSA-TIM-IMI-101126 | 198 | CF | Pediatric | the Netherlands | 0.12 | 2 | 2 | 4 | 0.5 | 8 | 1 | 16 | 16 | 0.25 | 4 | 2 | 32 | 1 |
| PSA-TIM-IMI-101127 | 179 | CF | Pediatric | the Netherlands | 0.25 | 8 | 2 | 1 | 0.5 | 1 | 0.25 | 4 | 1 | 4 | 128 | 8 | 128 | 1 |
| PSA-TIM-IMI-101128 | 175 | CF | Adult | the Netherlands | 0.12 | 0.5 | 2 | 2 | 1 | 2 | 0.12 | 2 | 2 | ≤0.03 | 0.5 | 0.25 | 1 | 1 |
| PSA-TIM-IMI-101129 | 170 | CF | Adult | the Netherlands | 0.5 | 32 | 128 | 16 | 2 | >256 | 0.5 | 4 | 256 | 8 | 8 | 2 | 32 | 0.5 |
| PSA-TIM-IMI-101130 | 155 | CF | Pediatric | the Netherlands | 0.12 | 8 | 1 | 1 | 1 | 0.5 | 0.12 | 4 | 0.5 | 0.25 | 2 | 0.5 | 4 | 0.5 |
| PSA-TIM-IMI-101131 | 132 | CF | Adult | the Netherlands | 0.06 | 2 | 2 | 2 | 0.5 | 8 | 0.25 | 2 | 16 | 0.25 | 4 | 0.5 | 2 | 1 |
| PSA-TIM-IMI-101132 | 108 | CF | Pediatric | the Netherlands | 0.06 | 4 | 2 | 8 | 1 | 16 | 0.25 | 1 | 16 | 0.06 | 4 | 1 | 8 | 0.5 |
| PSA-TIM-IMI-101133 | 27 | CF | Pediatric | the Netherlands | 0.25 | 8 | 2 | 2 | 1 | 1 | 0.5 | 2 | 8 | 4 | 8 | 1 | 16 | 1 |
| PSA-TIM-IMI-101134 | 17 | CF | Pediatric | the Netherlands | 0.12 | 8 | 4 | 4 | 1 | 8 | 0.25 | 1 | 32 | 1 | 2 | 0.5 | 8 | 1 |

BE= Bronchiectasis; CF = cystic fibrosis; NON-CF = Chronic pulmonary diseases, other than CF (not otherwise specified); COPD - Chronic Obstructive Pulmonary Disease

Ped = pediatric
