# Supplementary material for: Qualitative analysis of genomic mutations and antibiotic susceptibility testing of Pseudomonas aeruginosa isolates from chronic lung infections
Source: PLoS One. 2026 Mar 6;21(3):e0341613. doi: 10.1371/journal.pone.0341613 (PMC12965580; doi:10.1371/journal.pone.0341613)
Supplement: S3 Table — (PDF) [file pone.0341613.s003.pdf]

**S3 table 2.** Number of isolates/ST.

| ST | number |
|---|---|
| 9 | 1 |
| 17 | 11 |
| 27 | 18 |
| 41 | 2 |
| 108 | 4 |
| 111 | 3 |
| 116 | 1 |
| 132 | 3 |
| 146 | 28 |
| 148 | 1 |
| 155 | 10 |
| 164 | 2 |
| 170 | 6 |
| 175 | 4 |
| 179 | 6 |
| 189 | 1 |
| 198 | 6 |
| 231 | 1 |
| 235 | 1 |
| 238 | 1 |
| 241 | 1 |
| 242 | 1 |
| 244 | 5 |
| 245 | 5 |
| 252 | 6 |
| 253 | 11 |
| 254 | 1 |
| 257 | 1 |
| 260 | 9 |
| 261 | 2 |
| 262 | 3 |
| 266 | 1 |
| 267 | 3 |
| 270 | 1 |
| 273 | 1 |
| 274 | 7 |
| 281 | 1 |
| 296 | 4 |
| 299 | 2 |
| 308 | 1 |
| 313 | 4 |
| 316 | 2 |
| 319 | 2 |
| 348 | 4 |
| 357 | 1 |
| 360 | 2 |
| 365 | 1 |

| | |
|---|---|
| 379 | 2 |
| 381 | 4 |
| 385 | 4 |
| 386 | 1 |
| 389 | 2 |
| 390 | 1 |
| 395 | 10 |
| 406 | 30 |
| 439 | 2 |
| 480 | 3 |
| 483 | 1 |
| 484 | 1 |
| 485 | 1 |
| 487 | 1 |
| 488 | 1 |
| 491 | 3 |
| 492 | 3 |
| 496 | 2 |
| 497 | 5 |
| 498 | 1 |
| 499 | 3 |
| 500 | 2 |
| 505 | 1 |
| 506 | 4 |
| 508 | 12 |
| 511 | 1 |
| 513 | 1 |
| 514 | 2 |
| 521 | 1 |
| 525 | 1 |
| 537 | 2 |
| 549 | 8 |
| 550 | 3 |
| 553 | 2 |
| 560 | 2 |
| 564 | 2 |
| 569 | 7 |
| 575 | 3 |
| 605 | 1 |
| 609 | 1 |
| 612 | 3 |
| 617 | 1 |
| 620 | 2 |
| 633 | 1 |
| 640 | 1 |
| 645 | 3 |
| 664 | 1 |
| 667 | 2 |
| 676 | 2 |
| 701 | 2 |

| | |
|---|---|
| 702 | 1 |
| 708 | 1 |
| 760 | 1 |
| 773 | 1 |
| 775 | 2 |
| 782 | 2 |
| 807 | 1 |
| 821 | 1 |
| 829 | 1 |
| 836 | 1 |
| 845 | 1 |
| 859 | 1 |
| 865 | 1 |
| 871 | 2 |
| 883 | 1 |
| 885 | 2 |
| 908 | 1 |
| 918 | 1 |
| 919 | 2 |
| 1000 | 2 |
| 1046 | 1 |
| 1050 | 1 |
| 1058 | 1 |
| 1062 | 1 |
| 1068 | 1 |
| 1091 | 1 |
| 1101 | 1 |
| 1109 | 1 |
| 1135 | 1 |
| 1158 | 1 |
| 1180 | 1 |
| 1194 | 2 |
| 1203 | 5 |
| 1225 | 8 |
| 1226 | 4 |
| 1227 | 1 |
| 1228 | 2 |
| 1230 | 1 |
| 1231 | 2 |
| 1233 | 1 |
| 1238 | 2 |
| 1240 | 1 |
| 1251 | 1 |
| 1312 | 1 |
| 1315 | 1 |
| 1329 | 1 |
| 1342 | 1 |
| 1392 | 1 |
| 1635 | 1 |
| 1641 | 1 |

| | |
|---|---|
| 1648 | 1 |
| 1662 | 1 |
| 1712 | 2 |
| 1744 | 1 |
| 1748 | 1 |
| 1754 | 3 |
| 1811 | 1 |
| 1872 | 2 |
| 1874 | 3 |
| 1876 | 1 |
| 1883 | 2 |
| 1884 | 2 |
| 1886 | 1 |
| 1894 | 1 |
| 1907 | 1 |
| 1908 | 1 |
| 1909 | 1 |
| 1912 | 1 |
| 2048 | 1 |
| 2049 | 1 |
| 2098 | 1 |
| 2184 | 1 |
| 2211 | 3 |
| 2363 | 1 |
| 2409 | 1 |
| 2451 | 1 |
| 2455 | 3 |
| 2465 | 2 |
| 2495 | 1 |
| 2629 | 1 |
| 2685 | 1 |
| 2744 | 1 |
| 3006 | 2 |
| 3158 | 1 |
| 3159 | 1 |
| 3160 | 1 |
| 3161 | 2 |
| 3163 | 1 |
| 3164 | 1 |
| 3165 | 1 |
| 3166 | 1 |
| 3167 | 1 |
| 3168 | 1 |
| 3169 | 1 |
| 3170 | 1 |
| 3171 | 1 |
| 3172 | 1 |
| 3173 | 1 |
| 3174 | 1 |
| 3175 | 1 |

| | |
|---|---|
| 3176 | 1 |
| 3177 | 1 |
| 3178 | 1 |
| 3179 | 1 |
| 3180 | 1 |
| 3181 | 2 |
| 3183 | 1 |
| xx* | 2 |
| yy | 1 |

*xx and yy have a partial allele
