# Supplementary material for: Qualitative analysis of genomic mutations and antibiotic susceptibility testing of Pseudomonas aeruginosa isolates from chronic lung infections
Source: PLoS One. 2026 Mar 6;21(3):e0341613. doi: 10.1371/journal.pone.0341613 (PMC12965580; doi:10.1371/journal.pone.0341613)
Supplement: S4 Table — (PDF) [file pone.0341613.s004.pdf]

S4 table. OXA-type ß-lactamases.

		imipenem		meropenem		piperacillin/ tazobactam	
**OXA-type**	**amino acid changes compared to closest type**	**S (number/%)**	**R (number/%)**	**S (number/%)**	**R (number/%)**	**S (number/%)**	**R (number/%)**
50	Q17K	1/0.3	0/0.0	1/0.2	0/0.0	1/0.3	0/0.0
50		68/17.0	21/21.6	79/17.8	10/18.5	68/17.0	21/21.6
50	G196S	2/0.5	1/1.0	3/0.7	0/0.0	2/0.5	1/1.0
50	N115D	1/0.3	0/0.0	1/0.2	0/0.0	1/0.3	0/0.0
50	F6L,F168L	27/6.8	6/6.2	18/4.1	15/27.8	27/6.8	6/6.2
50	deletion after G206	1/0.3	0/0.0	1/0.2	0/0.0	1/0.3	0/0.0
50	F6L	9/2.3	2/2.1	11/2.5	0/0.0	8/2.0	3/3.1
395	S24T,D28N	1/0.3	0/0.0	1/0.2	0/0.0	1/0.3	0/0.0
395	V70M	1/0.3	0/0.0	1/0.2	0/0.0	1/0.3	0/0.0
395	K112E,A143T	1/0.3	1/1.0	1/0.2	1/1.9	1/0.3	1/1.0
395	A189T,K112E	3/0.8	0/0.0	3/0.7	0/0.0	3/0.8	0/0.0
395	D109E,N134D	3/0.8	0/0.0	3/0.7	0/0.0	3/0.8	0/0.0
395	K112E,A187T	1/0.3	0/0.0	1/0.2	0/0.0	1/0.3	0/0.0
395	A8V,D109E,N134D	3/0.8	1/1.0	3/0.7	1/1.9	3/0.8	1/1.0
395		6/1,5	0/0.0	6/1.4	0/0.0	6/1.5	0/0.0
395	K112E,E164D,A181T	2/0.5	1/1.0	3/0.7	0/0.0	2/0.5	1/1.0
395	31 amino acid changes	3/0.8	0/0.0	3/0.7	0/0.0	3/0.8	0/0.0
396		32/8.0	8/8.2	38/8.6	2/3.7	32/8.0	8/8.2
396	out-of-frame after L233	1/0.3	0/0.0	1/0.2	0/0.0	1/0.3	0/0.0
396	A100T	1/0.3	0/0.0	1/0.2	0/0.0	1/0.3	0/0.0
486	R46K	1/0.3	0/0.0	1/0.2	0/0.0	1/0.3	0/0.0
486		44/11.0	15/15.5	50/11.3	9/16.7	44/11	15/15.5
486	L170I	11/2.8	2/2.1	13/2.9	0/0.0	11/2.8	2/2.1
486	D109E	0/0.0	1/1.0	1/0.2	0/0.0	1/0.3	0/0.0
488		20/0.05	3/3.1	21/4.7	2/3.7	20/5.0	3/3.1
494	T16A,Q25R,K159N	6/1.5	0/0.0	6/1.4	0/0.0	6/1.5	0/0.0
494	T166P	0/0.0	1/1.0	1/0.2	0/0.0	0/0.0	1/1.0
494		59/14.8	10/10.3	65/14.7	4/7.4	59/14.8	10/10.3
494	H202R	1/0.3	0/0.0	1/0.2	0/0.0	1/0.3	0/0.0
494	G251D	1/0.3	0/0.0	1/0.2	0/0.0	1/0.3	0/0.0
846	R83K	4/1.0	2/2.1	6/1.4	0/0.0	4/1.0	2/2.1
846	A8T	1/0.3	0/0.0	1/0.2	0/0.0	1/0.3	0/0.0
846	I111V	1/0.3	0/0.0	1/0.2	0/0.0	1/0.3	0/0.0
846		3/0.8	0/0.0	3/0.7	0/0.0	3/0.8	0/0.0
846	A189T	0/0.0	1/1.0	1/0.2	0/0.0	0/0.0	1/1.0
846	H15P,T16A	2/0.5	0/0.0	2/0.5	0/0.0	2/0.5	0/0.0
847		28/7.0	8/8.2	28/6.3	8/14.8	28/7.0	8/8.2
851	R49L	4/1.0	1/1.0	5/1.1	0/0.0	4/1.0	1/1.0
851	D109E	1/0.3	0/0.0	1/0.2	0/0.0	1/0.3	0/0.0
901		3/0.8	1/1.0	4/0.9	0/0.0	3/0.8	1/1.0
902		6/1.5	2/2.1	7/1.6	1/1.9	6/1.5	2/2.1
902	K256T	0/0.0	1/1.0	1/0.2	0/0.0	0/0.0	1/1.0
904		23/5.8	6/6.2	29/6.5	0/0.0	23/5.8	6/6.2
905		8/2.0	1/1.0	9/2.0	0/0.0	9/2.3	0/0.0
905	P117L	1/0.3	0/0.0	1/0.2	0/0.0	1/0.3	0/0.0
914		4/1.0	0/0.0	4/0.9	0/0.0	4/1.0	0/0.0
937	R188S	1/0.3	0/0.0	1/0.2	0/0.0	1/0.3	0/0.0
937	F6L,K112E	1/0.3	0/0.0	1/0.2	0/0.0	1/0.3	0/0.0
